# ﻿Revision of the Neotropical water scavenger beetle genus *Novochares* Girón & Short (Coleoptera, Hydrophilidae, Acidocerinae)

**DOI:** 10.3897/zookeys.1171.104142

**Published:** 2023-07-20

**Authors:** Andrew Edward Z. Short, Jennifer C. Girón

**Affiliations:** 1 Department of Entomology & Nematology, University of Florida, Gainesville, FL, 32611, USA University of Florida Gainesville United States of America; 2 Natural Science Research Laboratory, Museum of Texas Tech University, Lubbock, TX 79409, USA Natural Science Research Laboratory, Museum of Texas Tech University Lubbock United States of America

**Keywords:** Aquatic beetles, integrative taxonomy, Neotropical Region, new species

## Abstract

The water scavenger beetle genus *Novochares* Girón & Short, 2021 is revised using a combination of adult morphological and DNA sequence data. Thirty-eight new species are described: *Novocharesaperito***sp. nov.** (Bolivia), *N.baca***sp. nov.** (Brazil, Ecuador, Peru, Suriname), *N.bidens***sp. nov.** (Brazil), *N.bisinuatus***sp. nov.** (Brazil), *N.clavieri***sp. nov.** (Brazil, French Guiana, Peru), *N.danta***sp. nov.** (Venezuela), *N.dentatus***sp. nov.** (Ecuador, Venezuela), *N.dicranospathus***sp. nov.** (Peru), *N.duo***sp. nov.** (Brazil, French Guiana, Guyana, Suriname, Venezuela), *N.fernandezae***sp. nov.** (Brazil, Peru, Venezuela), *N.florifer***sp. nov.** (Brazil), *N.furcatus***sp. nov.** (Brazil), *N.garciai***sp. nov.** (Venezuela), *N.garfo***sp. nov.** (Brazil), *N.geminus***sp. nov.** (Brazil), *N.kawsay***sp. nov.** (Ecuador, Peru), *N.latus***sp. nov.** (Brazil), *N.minor***sp. nov.** (Peru, Suriname, Venezuela), *N.mojenos***sp. nov.** (Bolivia), *N.mura***sp. nov.** (Brazil), *N.orchis***sp. nov.** (Brazil, French Guiana, Suriname), *N.pastinum***sp. nov.** (Ecuador), *N.pertusus***sp. nov.** (Brazil), *N.piaroa***sp. nov.** (Venezuela), *N.pilatus***sp. nov.** (Venezuela), *N.pume***sp. nov.** (Venezuela), *N.punctatostriatus***sp. nov.** (Brazil, French Guiana, Guyana, Peru, Suriname), *N.quadrispinus***sp. nov.** (Brazil, Guyana, Suriname), *N.spangleri***sp. nov.** (Peru), *N.tambopatense***sp. nov.** (Peru), *N.tenedor***sp. nov.** (Guyana, Venezuela), *N.triangularis***sp. nov.** (Bolivia, Brazil, Paraguay), *N.tridentis***sp. nov.** (Brazil), *N.trifurcatus***sp. nov.** (Peru), *N.unguis***sp. nov.** (Bolivia, Peru), *N.xingu***sp. nov.** (Brazil), and *N.yanomami***sp. nov.** (Venezuela), *N.yora***sp. nov.** (Peru). One new synonym is proposed: *N.carmona* (Short, 2005) **syn. nov.** was determined to be a junior subjective synonym of *N.chaquensis* (Fernández, 1982). *Novocharesinornatus* (d’Orchymont, 1926) is considered incertae sedis. Updated distributions and new records are provided for most previously described species in the genus. *Novocharessallaei* (Sharp, 1882) is considered native to the USA (Florida) and not an introduced species as previously suggested. *Novochares* now contains 52 species and spans the entire Neotropical region from Mexico to Argentina, including the Caribbean islands.

## Abstract (es)

El género de escarabajos acuáticos detritívoros *Novochares* Girón & Short, 2021, se revisa usando una combinación de datos morfológicos de los adultos y secuencias de ADN. Se describen 38 especies nuevas: *Novocharesaperito***sp. nov.** (Bolivia), *N.baca***sp. nov.** (Brasil, Ecuador, Perú, Surinam), *N.bidens***sp. nov.** (Brasil), *N.bisinuatus***sp. nov.** (Brasil), *N.clavieri***sp. nov.** (Brasil, Guyana Francesa, Perú), *N.danta***sp. nov.** (Venezuela), *N.dentatus***sp. nov.** (Ecuador, Venezuela), *N.dicranospathus***sp. nov.** (Perú), *N.duo***sp. nov.** (Brasil, Guyana, Guyana Francesa, Surinam, Venezuela), *N.fernandezae***sp. nov.** (Brasil, Perú, Venezuela), *N.florifer***sp. nov.** (Brasil), *N.furcatus***sp. nov.** (Brasil), *N.garciai***sp. nov.** (Venezuela), *N.garfo***sp. nov.** (Brasil), *N.geminus***sp. nov.** (Brasil), *N.kawsay***sp. nov.** (Ecuador, Perú), *N.latus***sp. nov.** (Brasil), *N.minor***sp. nov.** (Perú, Surinam, Venezuela), *N.mojenos***sp. nov.** (Bolivia), *N.mura***sp. nov.** (Brasil), *N.orchis***sp. nov.** (Brasil, Guyana Francesa, Surinam), *N.pastinum***sp. nov.** (Ecuador), *N.pertusus***sp. nov.** (Brasil), *N.piaroa***sp. nov.** (Venezuela), *N.pilatus***sp. nov.** (Venezuela), *N.pume***sp. nov.** (Venezuela), *N.punctatostriatus***sp. nov.** (Brasil, Guyana, Guyana Francesa, Perú, Surinam), *N.quadrispinus***sp. nov.** (Brasil, Guyana, Surinam), *N.spangleri***sp. nov.** (Perú), *N.tambopatense***sp. nov.** (Perú), *N.tenedor***sp. nov.** (Guyana, Venezuela), *N.triangularis***sp. nov.** (Bolivia, Brasil, Paraguay), *N.tridentis***sp. nov.** (Brasil), *N.trifurcatus***sp. nov.** (Perú), *N.unguis***sp. nov.** (Bolivia, Perú), *N.xingu***sp. nov.** (Brasil), and *N.yanomami***sp. nov.** (Venezuela), *N.yora***sp. nov.** (Perú). Se propone una nueva sinonimia: *N.carmona* (Short, 2005) **syn. nov.** se determinó como sinónimo subjetivo posterior de *N.chaquensis* (Fernández, 1982). *Novocharesinornatus* (d’Orchymont, 1926) se considera incertae sedis. Se proveen distribuciones actualizadas y nuevos registros para la mayoría de las especies descritas previamente en el género. *Novocharessallaei* (Sharp, 1882) es considerada nativa en los Estados Unidos de América (Florida) y no como una especie potencialmente introducida como se afirmaba previamente. *Novochares* ahora contiene 52 especies y se extiende a lo largo de toda la región neotropical, desde México hasta Argentina, incluyendo las islas del Caribe.

## ﻿Introduction

The last twenty years have seen considerable advances in our knowledge of the water scavenger beetle subfamily Acidocerinae. Much of this knowledge was summarized in two recent studies: a molecular phylogeny of the subfamily (Short et al. 2021) and a comprehensive monograph and catalog of species (Girón and Short 2021). These studies found that the New World species that had been placed in the nominate subgenus of *Helochares* Mulsant, 1844 in fact formed an unrelated clade which we described as *Novochares* Girón & Short, 2021. *Novochares* currently contains 15 described species and spans the entire Neotropical region, occurring from Mexico to Argentina, as well as in the Caribbean Islands. One species (*N.sallaei* (Sharp, 1882)) is known from Florida where it was suggested to be introduced (Young 1954; Epler 2010), but a review of the data suggests it is most likely native to the United States. Among genera of Neotropical Acidocerinae, *Novochares* is the most widely distributed as well as the most commonly collected. The taxonomic history of the genus extends back more than 220 years, when *N.abbreviatus* was described by Fabricius (1801). A few additional species were described over the subsequent 180 years, usually one or two at a time, with samples limited to a few localities within a particular country. It was not until the 1980s that any attempt was made to integrate information on the group, when Fernández (1982a, 1982b, 1989) published a series of studies focusing on southern South America that consolidated our knowledge of the group and described six new species, nearly doubling the species known at that time. Since then, only two additional species have been described: *N.carmona* (Short, 2005) from Costa Rica and *N.atlanticus* (Clarkson & Ferreira Jr, 2014) from São Paulo, Brazil.

As typical of many other genera of Acidocerinae, the external morphology of the lineage is relatively homogenous, with few discrete characters to easily separate species; usually these are only sufficient to sort specimens into a species group, but not enough for species identification. Fortunately, the aedeagus of *Novochares* is extremely complex and variable, exhibiting an extraordinary array of shapes and forms. This allows the aedeagus to be used as a relatively easy and straightforward diagnostic tool if males are available. Without males, few species can be identified with certainty, and identification of unassociated females is strongly discouraged. As we gathered material for this revision, we were shocked by the diversity in aedeagal forms and the number of putative species encountered, which was also noticed by Paul Spangler from the specimens he collected across South America in the 1970s (P. Spangler notes). We quickly realized the genus was significantly under-described and that tackling the taxonomy and systematics of *Novochares* was only possible with access to a large number of specimens covering its entire distributional range. To help us circumscribe species and understand the intraspecific limits of aedeagal variation, we employed an integrative approach that combined morphology with DNA sequence data from two genes: the mitochondrial gene COI, and the nuclear ribosomal gene 28S. Here we combine morphological and molecular data to (1) define species groups within *Novochares*, (2) redescribe the 15 previously described species, and (3) describe 38 new species. In addition, we clarify the status and define morphological features to clearly distinguish *N.abbreviatus* (Fabricius, 1801) and *N.oculatus* (Sharp, 1882), which have been historically confused, and discuss the native status of *N.sallaei* in the United States.

## ﻿Materials and methods

### ﻿Depositories of examined material

**CAS**California Academy of Sciences, San Francisco, CA (C. Grinter)

**FMNH**Field Museum of Natural History, Chicago, IL (C. Maier)

**CBDG**Center for the study of Biological Diversity, University of Guyana, Georgetown (G. Maharaj)

**CMNH** Carnegie Museum of Natural History

**DZRJ**Departamento de Zoologia, Universidade Federal do Rio de Janeiro (N. Ferreira Jr.)

**INPA**Instituto Nacional de Pesquisas da Amazônia, Manaus, Brazil (N. Hamada)

**IRNSB** Institute royal des Sciences naturelles de Belgique

**MALUZ**Museo de Artrópodos de la Universidad del Zulia, Maracaibo, Venezuela (J. Camacho, M. García)

**MACN**Museo Argentino de Ciencias Naturales, Buenos Aires, Argentina

**MCZ**Museum of Comparative Zoology, Cambridge, MA (C. Maier)

**MHNSM**Museo de Historia Natural de la Universidad Nacional Mayor de San Marcos (M. Alvarado)

**MIZA**Museo del Instituto de Zoología Agrícola, Maracay, Venezuela (L. Joly)

**MLP** Museo de Ciencias Naturales de La Plata

**MNHN**Muséum national d’Histoire naturelle, Paris, France (A. Mantilleri)

**NHMUK**United Kingdom, London, The Natural History Museum [formerly British Museum (Natural History BMNH; M. Barclay, C. Taylor)

**NZCS**National Zoological Collection of Suriname, Paramaribo (P. Ouboter, V. Kadosoe)

**PERC**Entomologica Research Collection, Purdue University, West Lafayette, IN.

**PUCE**Pontificia Universidad Católica del Ecuador, Quito, Ecuador (C. Kiel)

**SCC** Collection of Simon Clavier, Kourou, French Guiana

**SEMC**Snow Entomological Collection, University of Kansas, Lawrence, KS (A. Short)

**TTU-Z** Invertebrate Zoology Collection, Natural Science Research Laboratory, Museum of Texas Tech University, Lubbock, TX (J. Girón)

**USNM**U.S. National Museum of Natural History, Smithsonian Institution, Washington, DC (C. Micheli).

**WSU**M.T. James Entomological Collection, Washington State University, Pullman, WA (R. Zack)

### ﻿Morphological methods

More than 2000 specimens were examined. Specimen dissection and examination follows Girón and Short (2017). Descriptive sequence and morphological terminology follow Girón and Short (2021), especially when referring to the male genitalia. Species descriptions are given by species group and in alphabetical order, whereas in the genitalia images, species are grouped by similarity for ease of comparison. Maps were created using SimpleMappr (Shorthouse 2010). Type labels are cited verbatim in quotation marks.

### ﻿Molecular methods

Representatives of as many morphospecies as we could find suitable frozen tissue samples were included in the analysis. Additionally, for widespread or putatively variable species, we included multiple representatives for a total of 97 *Novochares* samples. Total genomic DNA extractions were performed on whole beetles using a DNeasy tissue kit (Qiagen, Alameda, CA). Vouchers (Table 1) are deposited at the University of Kansas (Lawrence, KS, USA) unless otherwise indicated in the material examined sections. We amplified the mitochondrial gene COI and the nuclear ribosomal gene 28S using the primers and PCR protocols provided in Short and Fikáček (2013). DNA sequences were assembled and edited in Geneious 8.0.5 (Biomatters, http://www.geneious.com), which was also used to calculate uncorrected pairwise distances for the gene COI. All sequences are deposited in GenBank (Table 1). We used the IQ-Tree webserver (Nguyen et al. 2015) to conduct a maximum likelihood analysis on the combined dataset, with each gene in a separate partition. The optimal model of substitution for each partition was selected using the Auto function in IQ-TREE. To assess nodal support, we performed 1000 ultrafast bootstrap replicates (Minh et al. 2013). We used a representative of the genus *Sindolus* to root the tree, as this was identified as the sister group to *Novochares* by Short et al. (2021).

**Table 1.** List of DNA vouchers and GenBank accessions.

Species	Voucher	Locality	COI	28S
*N.abbreviatus*	SLE1207	Venezuela: Sucre	OQ918469	N/A
*N.abbreviatus*	SLE1208	Venezuela: Delta Amacuro	OQ918470	OQ919169
*N.abbreviatus*	SLE1210	Suriname: Sipaliwini	OQ918471	N/A
*N.abbreviatus*	SLE1215	Suriname: Sipaliwini	OQ918472	OQ919169
*N.abbreviatus*	SLE1221	Guyana: Region 8	OQ918473	OQ919170
*N.abbreviatus*	SLE1237	Venezuela: Zulia	OQ918474	OQ919171
*N.abbreviatus*	SLE1240	Venezuela: Cojedes	OQ918475	OQ919172
*N.abbreviatus*	SLE2083	Brazil: Pará	OQ918476	OQ919173
*N.abbreviatus*	SLE2086	Brazil: Mato Grosso do Sul	OQ918477	OQ919174
*N.abbreviatus*	SLE2087	Brazil: Goiás	OQ918478	OQ919175
*N.abbreviatus*	SLE2152	Peru: Loreto	OQ918479	OQ919176
*N.abbreviatus*	SLE1180	Costa Rica: Guanacaste	MW351395	MW351051
*N.abbreviatus*	SLE1217	Venezuela: Barinas	MW351401	MW351057
*N.abbreviatus* group	SLE1193	Venezuela: Amazonas	OQ918520	N/A
*N.abbreviatus* group	SLE1203	Venezuela: Amazonas	OQ918521	OQ919216
*N.abbreviatus* group	SLE1271	Brazil: Amazonas	OQ918522	N/A
*N.abbreviatus* group	SLE1837	Brazil: Mato Grosso do Sul	OQ918523	OQ919217
*N.abbreviatus* group	SLE2006	Brazil: Rondônia	OQ918524	OQ919218
*N.abbreviatus* group	SLE2099	Brazil: Roraima	OQ918526	OQ919220
*N.abbreviatus* group	SLE2468	Peru: Madre de Dios	OQ918528	OQ919222
*N.abbreviatus* group	SLE1197	Venezuela: Guárico	MW351399	MW351055
*N.baca*	SLE1513	Brazil: Pará	OQ918480	OQ919177
*N.baca*	SLE1617	Brazil: Pará	OQ918481	OQ919178
*N.baca*	SLE2081	Brazil: Pará	OQ918482	OQ919179
*N.baca*	SLE2137	Peru: Madre de Dios	OQ918483	OQ919180
*N.baca*	SLE1162	Suriname: Sipaliwini	MW351394	MW351050
*N.clavieri*	SLE1514	Brazil: Pará	OQ918484	OQ919181
*N.clavieri*	SLE2085	Brazil: Amapá	OQ918485	OQ919182
*N.clavieri*	SLE2420	French Guiana	OQ918486	OQ919183
*N.cochlearis*	SLE1175	Venezuela: Monagas	OQ918487	OQ919184
*N.cochlearis*	SLE1628	Brazil: Bahia	OQ918488	OQ919185
*N.cochlearis*	SLE1922	Brazil: Amapá	OQ918489	OQ919186
*N.cochlearis*	SLE2080	Brazil: Minas Gerais	OQ918490	OQ919187
*N.cochlearis*	SLE2412	French Guiana	OQ918491	N/A
*N.cochlearis*	SLE1196	Venezuela: Guárico	MW351398	MW351054
*N.coya*	SLE1218	Guyana: Region 8	MW351402	MW351058
*N.coya*	SLE2463	Peru: Madre de Dios	OQ918492	OQ919188
*N.danta*	SLE1399	Venezuela: Amazonas	OQ918493	OQ919189
*N.dentatus*	SLE1188	Ecuador: Pastaza	MW351396	MW351052
*N.dentatus*	SLE1199	Venezuela: Amazonas	MW351400	MW351056
*N.duo*	SLE1209	Suriname: Sipaliwini	N/A	OQ919190
*N.duo*	SLE1211	Suriname: Sipaliwini	N/A	OQ919191
*N.duo*	SLE1242	Guyana: Region 8	MW351405	MW351061
*N.duo*	SLE1906	Brazil: Amazonas	OQ918494	OQ919192
*N.fernandezae*	SLE1992	Brazil: Amazonas	N/A	OQ919193
*N.fernandezae*	SLE1099	Peru: Madre de Dios	MW351368	MW351025
*N.florifer*	SLE1991	Brazil: Amazonas	OQ918495	OQ919194
*N.furcatus*	SLE1931	Brazil: Amazonas	OQ918496	OQ919195
*N.furcatus*	SLE2103	Brazil: Mato Grosso do Sul	OQ918497	OQ919196
*N.furcatus*	SLE1263	Brazil: Amazonas	MW351376	MW351032
*N.garfo*	SLE2003	Brazil: Rondônia	OQ918498	OQ919197
*N.garfo*	SLE2097	Brazil: Rondônia	OQ918499	OQ919198
*N.geminus*	SLE2092	Brazil: Mato Grosso do Sul	OQ918500	OQ919199
*N.guadelupensis*	SLE1803	Suriname: Sipaliwini	OQ918501	OQ919200
*N.guadelupensis*	SLE2117	Suriname: Sipaliwini	OQ918502	OQ919201
*N.guadelupensis*	SLE2421	French Guiana	OQ918503	OQ919202
*N.guadelupensis*	SLE1200	Guyana: Region 9	MW351350	MW351008
*N.kawsay*	SLE2467	Peru: Madre de Dios	OQ918504	OQ919203
*N.latus*	SLE2039	Brazil: Rondônia	OQ918505	OQ919204
*N.minor*	SLE2143	Peru: Loreto	OQ918506	OQ919205
*N.minor*	SLE535	Suriname: Para	MW351360	MW351018
*N.mura*	SLE1973	Brazil: Amazonas	OQ918507	OQ919206
*N.orchis*	SLE1851	Brazil: Amapá	OQ918508	OQ919207
*N.orchis*	SLE2415	French Guiana	OQ918509	N/A
*N.orchis*	SLE1214	Suriname: Sipaliwini	MW351375	MW351031
*N.piaroa*	SLE1194	Venezuela: Amazonas	N/A	OQ919208
*N.pilatus*	SLE1204	Venezuela: Bolívar	OQ918510	OQ919209
*N.pilatus*	SLE1241	Venezuela: Barinas	MW351404	MW351060
*N.punctatostriatus*	SLE1098	Peru: Madre de Dios	MW351393	MW351049
*N.punctatostriatus*	SLE1191	Bolivia: Santa Cruz	MW351397	MW351053
*N.punctatostriatus*	SLE1802	Suriname: Sipaliwini	OQ918513	OQ919212
*N.punctatostriatus*	SLE1969	Brazil: Amazonas	OQ918514	N/A
*N.punctatostriatus*	SLE2037	Brazil: Rondônia	OQ918515	N/A
*N.punctatostriatus*	SLE2090	Brazil: Rondônia	OQ918516	OQ919213
*N.punctatostriatus*	SLE2094	Brazil: Amapá	OQ918517	OQ919214
*N.punctatostriatus*	SLE2471	Peru: Madre de Dios	OQ918518	OQ919215
*N.punctatostriatus*	SLE452	Suriname: Sipaliwini	OQ918511	OQ919210
*N.punctatostriatus*	SLE515	French Guiana	OQ918512	OQ919211
*N.quadrispinus*	SLE537	Suriname: Sipaliwini	OQ918519	N/A
*N.quadrispinus*	SLE536	Suriname: Sipaliwini	MW351361	MW351019
*N.sallaei*	SLE1212	Guatemala	MW351355	MW351013
*N.* sp.	SLE2043	Bolivia: Villa Tunari	OQ918525	OQ919219
*N.* sp.	SLE2145	Peru: Loreto	OQ918527	OQ919221
*N.spangleri*	SLE2472	Peru: Madre de Dios	OQ918529	OQ919223
*N.tectiformis*	SLE1172	Suriname: Sipaliwini	OQ918530	OQ919224
*N.tectiformis*	SLE1905	Brazil: Amazonas	OQ918531	OQ919225
*N.tectiformis*	SLE1981	Brazil: Amazonas	OQ918532	OQ919226
*N.tectiformis*	SLE2089	Brazil: Rondônia	OQ918533	OQ919227
*N.tectiformis*	SLE2093	Brazil: Mato Grosso do Sul	OQ918534	OQ919228
*N.tectiformis*	SLE2095	Brazil: Mato Grosso do Sul	OQ918535	OQ919229
*N.tectiformis*	SLE1220	Guyana: Region 9	MW351403	MW351059
*N.tectiformis*	SLE448	Suriname: Sipaliwini	MW351357	MW351015
*N.tenedor*	SLE1219	Guyana: Region 8	OQ918536	OQ919230
*N.tenedor*	SLE1205	Venezuela	MW351374	MW351030
*N.trifurcatus*	SLE2147	Peru: Loreto	OQ918537	OQ919231
*N.unguis*	SLE2136	Peru: Madre de Dios	OQ918538	OQ919232
*N.unguis*	SLE2460	Peru: Madre de Dios	OQ918539	OQ919233
*Sindolus* sp.	SLE1239	Venezuela: Cojedes	OQ918540	OQ919234

## ﻿Results

**Figure 1.**
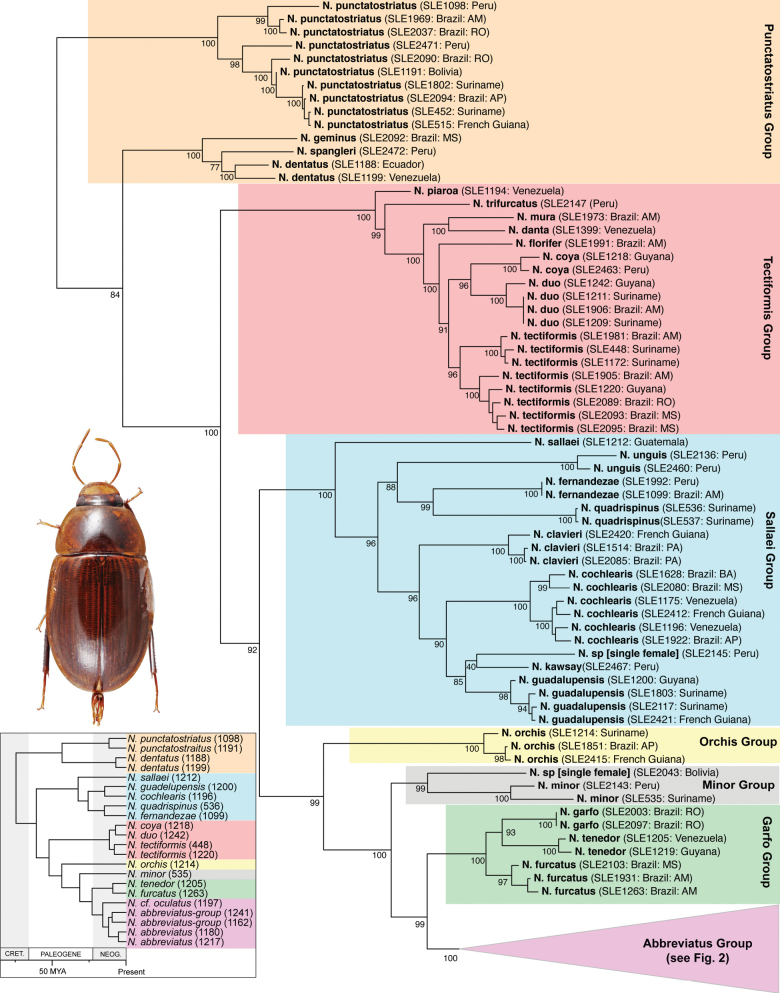
Phylogeny of *Novochares* spp. Part 1: *punctatostriatus*, *tectiformis*, *sallaei*, *orchis*, *minor*, and *garfo* species groups. Inferred from COI and 28S combined sequence data. Numbers next to taxon names are extraction numbers (see Table 1). The *Novochares* portion of the phylogeny of Acidocerinae based on five gene fragments as presented in Short et al. (2021) is reproduced in the lower left corner for comparative purposes.

**Figure 2.**
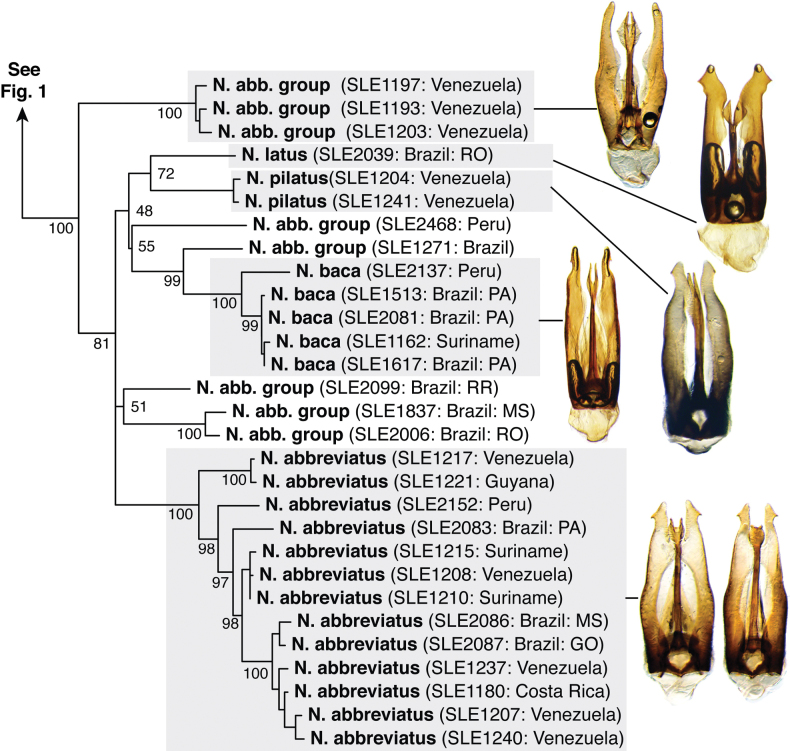
Phylogeny of *Novochares* spp. Part 2: *abbreviatus* species group. Inferred from COI and 28S combined sequence data. Numbers next to taxon names are extraction numbers (see Table 1).

The maximum likelihood analysis of the two-gene dataset resulted in a well-resolved and fairly well-supported tree (Figs 1, 2). The tree was largely concordant with relationships recovered in the 5-gene study of the Acidocerinae (Short et al. 2021), though that study contained a much smaller (but representative) number of *Novochares* (see Fig. 1, insert). Based on this analysis, and because the genus is so large and unwieldy, we have partitioned *Novochares* into eight species groups. These groups serve as a way to organize and in some cases recognize the diversity of forms found within the genus. They are meant to serve as a phylogenetically-informed identification aid. Seven of the eight species groups that we had molecular data for are strongly supported as being monophyletic in both Short et al. (2021) and this study. The most early diverging lineage (the *punctatostriatus* species group) was resolved as monophyletic in Short et al. (2021) but as a pair of sequentially branching (paraphyletic) lineages in our two-gene analysis. We prefer to treat them as a single species-group as there are a number of rather unique characters that can be used to assist in diagnosing the group.

In our integrated review of DNA data and morphology, we found broad agreement between genetic distance and morphological divergence. With a few exceptions, the maximum intraspecific pairwise genetic distance in COI was less than 5.0%, and the minimum interspecific difference was greater than 6.0%. Three species included multiple terminals with a maximum pairwise genetic distance in COI greater than 5.0%: *N.cochlearis* (Fernández, 1982) (5.1%), *N.tectiformis* (Fernández, 1982) (5.4%), and *N.punctatostriatus* sp. nov. (8.9%). All three of these species have very broad ranges in South America, all extending from Suriname and Guyana south to central (Rondônia) or south central (Mato Grosso du Sul) Brazil and Bolivia. In the case of *N.cochlearis* and *N.tectiformis*, there is modest variation in the shape of the aedeagus across their ranges (Figs 18M–P, 25G–L) but we were unable to detect a clear pattern or division that corresponds to the molecular data we had available. Consequently, we view these as widespread, variable species. In the case of *N.punctatostriatus*, the genetic variation is extreme and also appears to be potentially geographically structured. We believe what we are calling *N.punctatostriatus* is almost certainly a species complex of two or more species. However, we were unable to find any clear, consistent morphological character to use to break up this genetic lineage into more than one species. It is important to note that very large geographic distances did not correlate to high genetic distance in other widespread species; for example, the very widespread *N.abbreviatus* only varied 3.9% from Costa Rica to southern Brazil.

Three species pairs were separated from their closest congener by a genetic distance of less than 6.0%: *N.spangleri* sp. nov./*N.dentatus* sp. nov. (5.8%), *N.coya* (Fernández, 1982)/*N.duo* sp. nov. (5.4%), and *N.latus* sp. nov./ *N.pilatus* sp. nov. (5.0%). In all three of these cases, there are clear (and in some cases very substantial) morphological differences in the form of the aedeagus which allowed us to easily separate and diagnose each species.

Within the *abbreviatus* species group, which is the most common and widespread lineage of *Novochares*, there was an exceptionally large range of genetic variation among specimens and lineages that seemingly had only subtle variations in the genitalia. At the same time, there were species (e.g., *N.abbreviatus*) that also exhibit intraspecific variation in the same characters. This made establishing species boundaries more of a challenge. As species of this group are already externally indistinguishable, we did not want to describe species based on either DNA alone or overlapping morphological variation, which would cause chaos in applying these species names in the future. We also recognize that even with the many hundreds of specimens we examined, we still lacked material from large swaths of South America that will no doubt add yet more variation both within and between the species we have chosen to recognize. We opted to be relatively conservative, and only delineated in this group species that were genetically distinct and clearly morphologically differentiated. This leaves several lineages and aedeagal forms that likely represent additional new species to future studies.

Two terminals in the tree (Fig. 1; SLE2043 from Bolivia in the *minor* species group and SLE2145 from Peru in the *sallaei* species group) are unassociated single female specimens. We believe they likely represent additional new species, but we cannot exclude the possibility that they are females of other described species for which suitable molecular material was not available.

### ﻿List of species

The full list of species currently described in *Novochares*, along with their general distributions is presented in Table 2.

**Table 2.** Checklist of *Novochares* species organized by species group, with their known distributions. Asterisks (*) denote new country records for previously described species. Question marks (?) indicate localities that need verification, pending DNA data availability.

***Novocharesabbreviatus* species group**	
1. *Novocharesabbreviatus* (Fabricius, 1801)	Argentina, Bolivia, Brazil (Espírito Santo, Mato Grosso do Sul, Pará, Pernambuco, Piauí, Rio Grande do Norte, Roraima, São Paulo), Colombia, Costa Rica, Cuba, Dominica*, Dominican Republic*, French Guiana, Guadeloupe*, Guyana*, Nicaragua*, Panama, Paraguay, Peru*, Puerto Rico*, Suriname, St. Thomas*, Trinidad and Tobago*, Venezuela
2. *Novocharesbaca* sp. nov.	Brazil (Pará), Ecuador, Peru, Suriname
3. *Novochareslatus* sp. nov.	Brazil (Rondônia)
4. *Novocharesoculatus* (Sharp, 1882)	Belize*, Colombia*, Costa Rica, Guatemala, Mexico, Panama. Argentina?, Brazil?, Paraguay? the Antilles? (Grenada?, St. Vincent?)
5. *Novocharespallipes* (Brullé, 1841)	Argentina, Uruguay
6. *Novocharespilatus* sp. nov.	Venezuela
***Novocharesaperito* species group**	
7. *Novocharesaperito* sp. nov.	Bolivia
***Novocharesgarfo* species group**	
8. *Novocharesbidens* sp. nov.	Brazil (Mato Grosso)
9. *Novocharesfurcatus* sp. nov.	Brazil (Mato Grosso do Sul, Rondônia)
10. *Novocharesgarfo* sp. nov.	Brazil (Amazonas, Mato Grosso do Sul, Pará, Roraima), Bolivia
11. *Novocharestenedor* sp. nov.	Guyana, Venezuela
***Novocharesminor* species group**	
12. *Novocharesminor* sp. nov.	Peru, Suriname, Venezuela
***Novocharesorchis* species group**	
13. *Novocharesorchis* sp. nov.	Brazil (Amapá, Amazonas, Rondônia), French Guiana, Suriname
***Novocharespunctatostriatus* species group**	
14. *Novocharesdentatus* sp. nov.	Ecuador, Venezuela
15. *Novocharesgeminus* sp. nov.	Brazil (Mato Grosso do Sul)
16. *Novocharespertusus* sp. nov.	Brazil (Goiás)
17. *Novocharespunctatostriatus* sp. nov.	Brazil (Amapá, Amazonas, Rondônia, São Paulo), French Guiana, Guyana, Peru, Suriname
18. *Novocharesspangleri* sp. nov.	Peru
19. *Novocharestriangularis* sp. nov.	Bolivia, Brazil (Goiás, Minas Gerias, São Paulo), Paraguay
***Novocharessallaei* species group**	
20. *Novocharesatratus* (Bruch, 1915)	Argentina, Brazil (Bahia, Espírito Santo, Mato Grosso do Sul, Minas Gerais, Rio de Janeiro), Paraguay
21. *Novocharesbisinuatus* sp. nov.	Brazil (Goiás)
22. *Novochareschaquensis* (Fernández, 1982)	Argentina, Bolivia*, Brazil (Mato Grosso, Mato Grosso do Sul, São Paulo), Colombia*, Costa Rica*, Ecuador*, Guyana*, Panama*, Peru*, Trinidad and Tobago*, Venezuela*
*N.carmona* (Short, 2005), syn. nov.	
23. *Novocharesclavieri* sp. nov.	Brazil (Amapá, Pará), French Guiana, Peru
24. *Novocharescochlearis* (Fernández, 1982)	Argentina, Bolivia*, Brazil* (Amapá, Bahia, Minas Gerais, Roraima, São Paulo), French Guiana*, Guyana*, Paraguay, Suriname*, Trinidad and Tobago*, Venezuela*
25. *Novocharesdicranospathus* sp. nov.	Peru
26. *Novocharesfernandezae* sp. nov.	Brazil (Amazonas), Peru, Venezuela
27. *Novocharesgarciai* sp. nov.	Venezuela
28. *Novocharesguadelupensis* (d’Orchymont, 1926)	Brazil* (Pará, Roraima), French Guiana*, Guadeloupe, Guyana*, Peru*, Puerto Rico*, Suriname*, Venezuela*
29. *Novochareskawsay* sp. nov.	Ecuador, Peru
30. *Novocharespastinum* sp. nov.	Ecuador
31. *Novocharespichilingue* (Fernández, 1989)	Ecuador
32. *Novocharesquadrispinus* sp. nov.	Brazil (Para), Guyana, Suriname
33. *Novocharessallaei* (Sharp, 1882)	Belize, Costa Rica, Guatemala*, Mexico, USA (Florida)
34. *Novocharestridentis* sp. nov.	Brazil (Goiás)
35. *Novocharesunguis* sp. nov.	Bolivia, Peru
36. *Novocharesyanomami* sp. nov.	Venezuela
***Novocharestectiformis* species group**	
37. *Novocharesatlanticus* (Clarkson & Ferreira Jr, 2014)	Brazil (São Paulo, Rio de Janeiro)
38. *Novocharesbolivianus* (Fernández, 1989)	Bolivia
39. *Novocharescoya* (Fernández, 1982)	Bolivia, French Guiana*, Guyana*, Peru*, Suriname*, Trinidad and Tobago*, Venezuela*
40. *Novocharesdanta* sp. nov.	Venezuela
41. *Novocharesduo* sp. nov.	Brazil (Amazonas, Pará), French Guiana, Guyana, Suriname, Venezuela
42. *Novocharesflorifer* sp. nov.	Brazil (Amazonas)
43. *Novocharesmojenos* sp. nov.	Bolivia
44. *Novocharesmura* sp. nov.	Brazil (Amazonas)
45. *Novocharespiaroa* sp. nov.	Venezuela
46. *Novocharespume* sp. nov.	Venezuela
47. *Novocharestambopatense* sp. nov.	Peru
48. *Novocharestectiformis* (Fernández, 1982)	Argentina, Bolivia*, Brazil (Amapá, Amazonas, Mato Grosso do Sul, Paraná, Rondônia, São Paulo), Guyana*, Ecuador*, French Guiana*, Paraguay, Suriname*, Venezuela
49. *Novocharestrifurcatus* sp. nov.	Peru
50. *Novocharesxingu* sp. nov.	Brazil (Pará)
51. *Novocharesyora* sp. nov.	Peru
**Incertae sedis**	
52. *Novocharesinornatus* (d’Orchymont, 1926)	Brazil (São Paulo), French Guiana

### ﻿Characters of taxonomic importance

With the exception of species in the *punctatostriatus* species group, members of *Novochares* are extremely uniform in their external morphology and most species are virtually indistinguishable from each other without dissecting male specimens. Therefore, for the most part, diagnostic features in this genus are limited to features of the aedeagus, and overall, there are no external characteristics that help distinguish species, but rather only species groups. In Table 3, a few features that can be used for species group recognition are provided.

**Table 3.** Summary of diagnostic features of *Novochares* species groups.

Species group	Body length in mm	Dorsal coloration	Maxillary palps / head width ratio	Posterior elevation of mesoventrite
*abbreviatus*	5.5–8.1	pale brown to yellowish brown	1.1–1.6	broadly and somewhat triangularly elevated with low medial longitudinal ridge extending anteriorly
*aperito*	4.9	orange	1.1	transversely elevated
*garfo*	4.9–6.5	pale brown (orange to yellowish)	1.3	broadly elevated, somewhat transverse, often with glabrous longitudinal ridge extending anteriorly
*minor*	4.2–5.4	very dark brown	1.1	broadly and somewhat transversely elevated, with medial longitudinal glabrous patch extending anteriorly
*orchis*	7.3–9.3	dark brown and sheeny	1.3	elevated as a triangular pyramid, with medial longitudinal ridge extending anteriorly
*punctatostriatus*	4.7–8.8	dark brown	0.8–1.2	transverse, usually blunt and low
*sallaei*	4.7–8.0	brown to dark brown or reddish brown	0.8–1.8	weakly and/or broadly elevated, with low or weak medial longitudinal ridge extending anteriorly
*tectiformis*	6.2–9.5	brown to dark brown, sometimes sheeny	1.1– 1.6	usually elevated as a triangular pyramid, with posterior face somewhat bisinuate or concave and medial longitudinal ridge extending anteriorly

**Body size.***Novochares* specimens range in size from 4.2 to 9.5 mm in length. Species groups can be somewhat categorized into size groups, with the *abbreviatus*, *aperito*, *garfo*, *punctatostriatus*, and *sallaei* species groups ranging between 4.7 and 8.0 mm, and most species in the *orchis* and the *tectiformis* species groups ranging between 6.2 and 9.0 mm. The *minor* species group contains the smallest *Novochares*, ranging between 4.2 and 5.4 mm.

**Coloration.** Usually uniformly dark brown, sometimes orange or pale brown, often with paler margins (e.g., Fig. 17). Paler coloration on specimens is usually an indicator of teneral stages. Nevertheless, the *abbreviatus* and *garfo* species groups tend to exhibit paler colorations, even in fully sclerotized specimens (Fig. 7A, B, D). Some species, especially in the *orchis* and the *tectiformis* species groups have a characteristic opalescent sheen on their dark brown surface (e.g., Fig. 24A, B).

**Punctation.** The ground punctation in *Novochares* ranges from very shallow to moderately marked. In the *punctatostriatus* species group, the elytral serial punctures become distinct along the lateral and posterior regions of the elytra, but the disc region lacks serial punctures (e.g., Fig. 14A–C). In species with no evident serial punctures, the fifth elytral series is usually noticeable by the presence of a weak row of scarce systematic punctures (e.g., Fig. 14D). Systematic punctures are often only weakly marked, hence they are noticeable only by the presence of setae; they may be more visible along the lateral and posterior regions of the elytra.

**Posterior elevation of mesoventrite.** This elevation is usually simply and broadly bulging (Fig. 6B); sometimes the bulge is somewhat pyramidal and posteriorly impressed; usually this impression is posteriorly evenly curved, and sometimes there is a weak, medial, longitudinal ridge posterior to this impression, making the whole elevation resemble a human nose. Usually, the bulge extends anteriorly as a low, shiny, and glabrous longitudinal ridge (Fig. 6A, B). In general, species in the *abbreviatus*, *aperito*, *garfo*, *minor*, *punctatostriatus*, and *sallaei* species groups have a relatively simple and somewhat transverse posterior elevation of the mesoventrite, whereas species in the *orchis* and *tectiformis* species groups have the pyramidal and posteriorly impressed posterior elevation of the mesoventrite.

**Aedeagus.** The aedeagal form in *Novochares* fits the category of ‘divided aedeagus’ proposed by Girón and Short (2021): parameres separated from each other for most of their lengths; median lobe divided in dorsal and ventral plates; dorsal plate of median lobe usually apically bifurcated; basal piece shorter than parameres (the apparent asymmetries shown in aedeagal figures are caused by the dissection or preservation method and do not constitute diagnostic features); gonopore usually clearly visible, variable in positioning along median lobe. The range of shapes of the dorsal plate of the median lobe and the apical region of the parameres in *Novochares* are the most variable recorded for Acidocerinae to date.

### ﻿Taxonomy

#### ﻿ Novochares

Taxon classificationAnimaliaColeopteraHydrophilidae

Girón & Short, 2021

13CE84D4-AE73-5174-B575-6F1D070C514E

Novochares Girón & Short, 2021: 87.

##### Type species.

*Helocharestectiformis* Fernández, 1982 by original designation.

##### Diagnosis.

**Figure 3.**
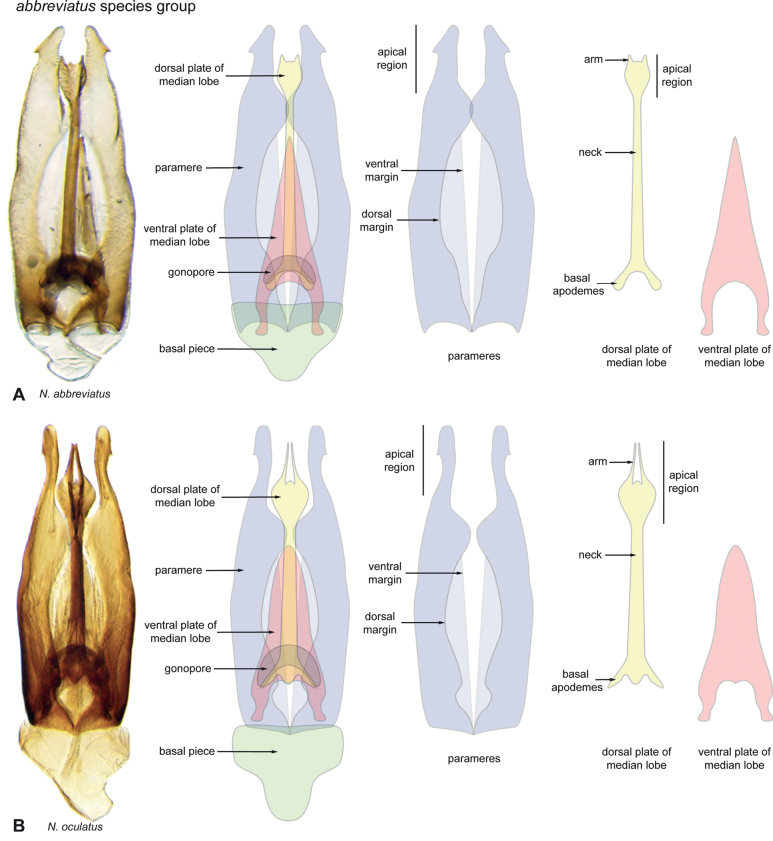
Aedeagi of the *Novocharesabbreviatus* species group along with schematic representations of the whole, the parameres (in blue), and the dorsal (in yellow) and ventral (in red) plates of the median lobe **A***N.abbreviatus***B***N.oculatus*.

**Figure 4.**
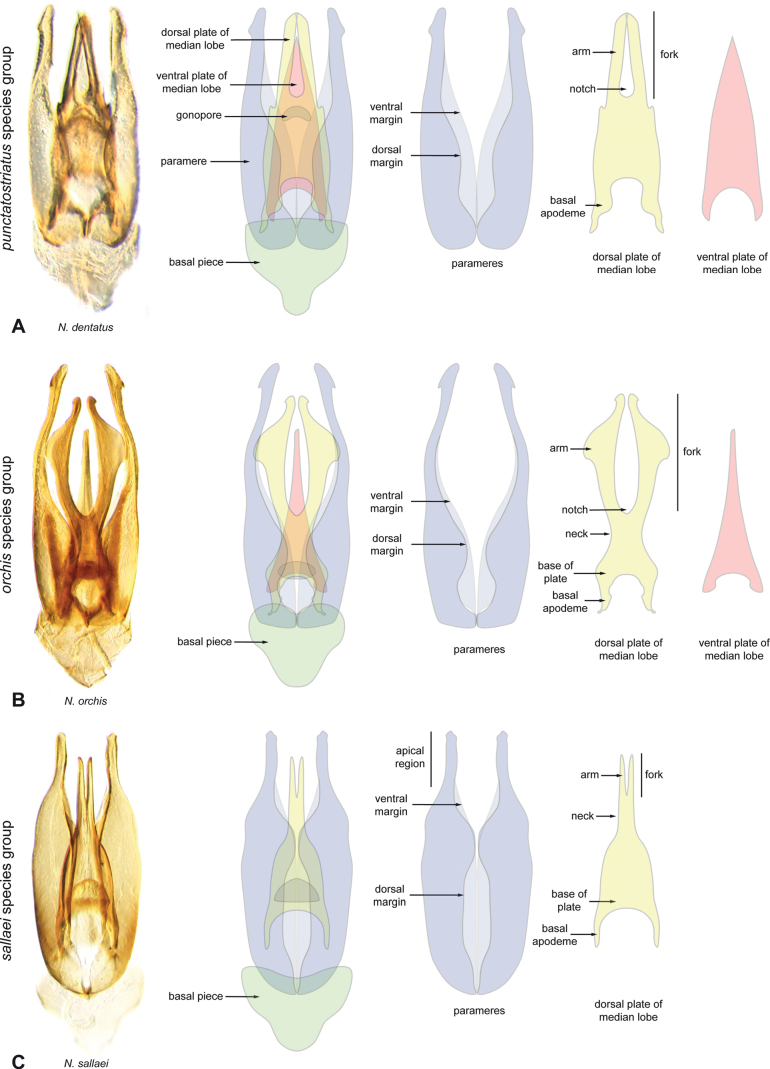
Aedeagi of the *Novocharespunctatostriatus*, *sallaei*, and *orchis* species groups along with schematic representations of the whole, the parameres (in blue), and the dorsal (in yellow) and ventral (in red) plates of the median lobe **A***N.dentatus***B***N.orchis***C***N.sallaei*.

**Figure 5.**
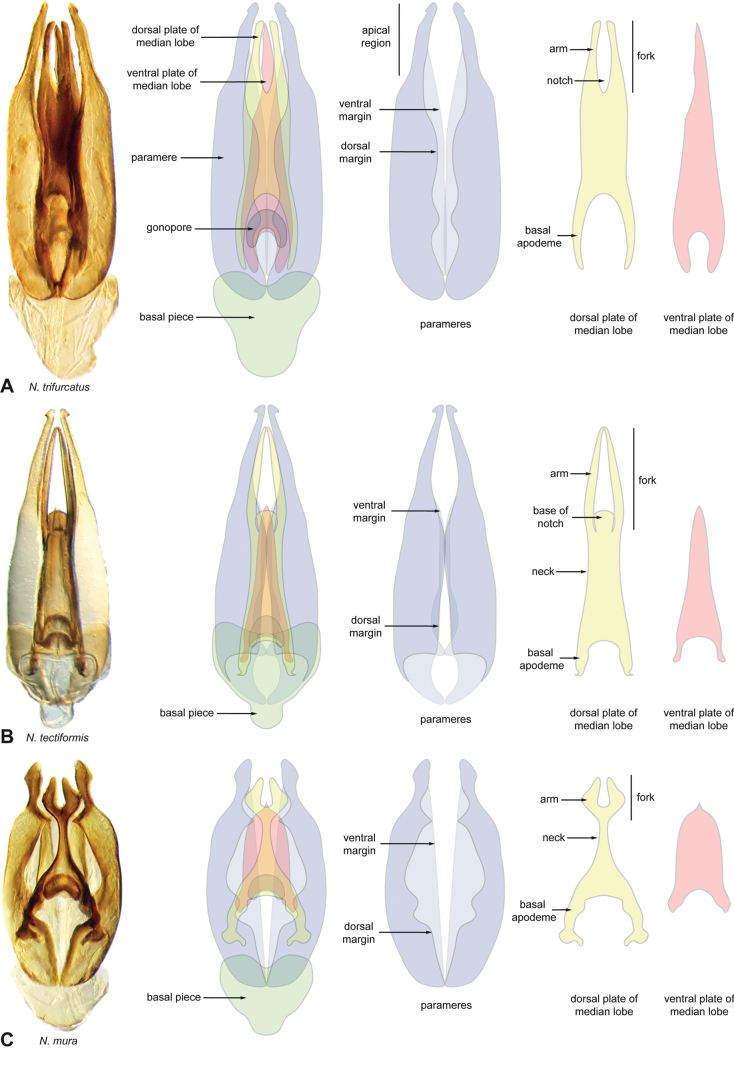
Aedeagi of the *Novocharestectiformis* species group along with schematic representations of the whole, the parameres (in blue), and the dorsal (in yellow) and ventral (in red) plates of the median lobe **A***N.trifurcatus***B***N.tectiformis***C***N.mura*.

**Figure 6.**
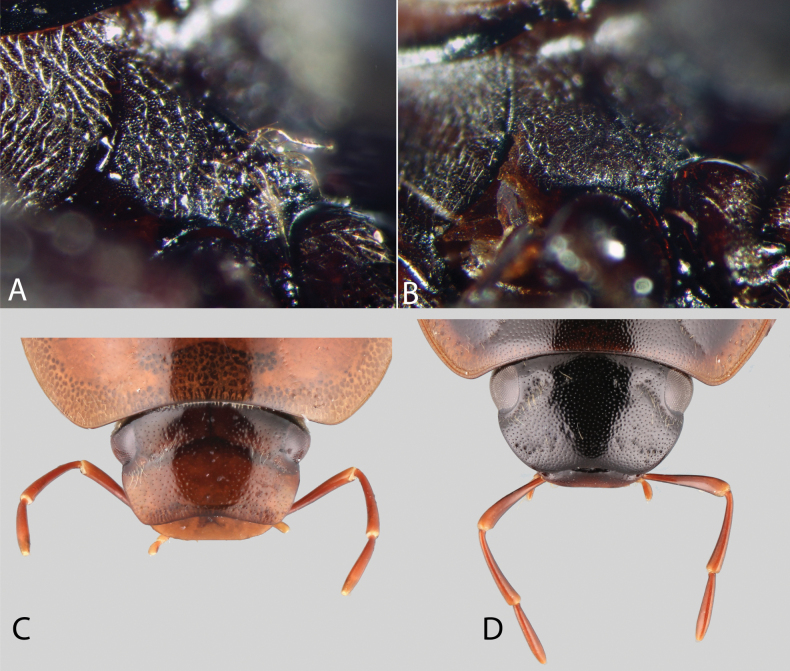
Morphological features of *Novochares* spp. **A, B** mesoventrite **C, D** dorsal view of the head **A***N.tectiformis***B***N.cochlearis***C***Aulonocharesligulatus***D***N.pertusus*.

**Figure 7.**
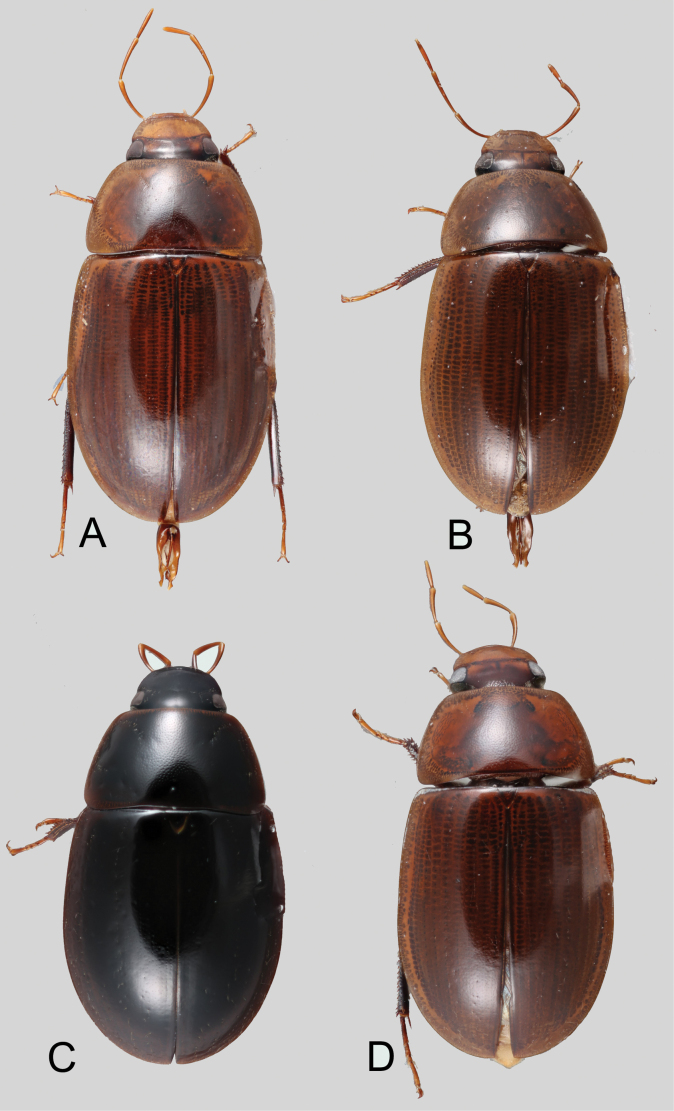
Dorsal habitus of *Novochares* spp. **A***N.abbreviatus***B***N.pilatus***C***N.minor***D***N.tenedor*.

(Slightly modified from Girón and Short 2021). Body length 4.2–9.5 mm. Body shape oval in dorsal view (Figs 7, 14, 17, 24); slightly to moderately convex in lateral view, with dorsal outline nearly flat along anterior 1/2 of elytra, or somewhat evenly curved. Coloration usually uniformly dark brown (Figs 14, 17, 24), sometimes orange or pale brown (Fig. 7), often paler along margins (e.g., Fig. 14A–C); opalescent sheen frequent in larger and darker species; ground punctation from very shallow (e.g., Fig. 17D) to moderately marked (e.g., Fig. 14A). Shape of head trapezoid (Fig. 6D). Eyes relatively large, not emarginated anteriorly, usually projected from outline of head (Fig. 6D). Clypeus trapezoid, with anterior margin broadly and roundly emarginate (Fig. 6D); membranous preclypeal area sometimes visible. Labrum fully exposed. Mentum with lateral longitudinal crenulations, lateral oblique ridges, and transverse crenulations along antero-medial area. Antennae with nine antennomeres; cupule strongly asymmetric, with rounded outline; antennomere 9 slightly to 2× longer than antennomere 7. Maxillary palps slender, moderately long, 0.8 (*N.pastinum* sp. nov.) to 1.8× (*N.yanomami* sp. nov.) the width of head; inner margin of maxillary palpomere 2 weakly and evenly curved to nearly straight, outer margin evenly curved or curved along apical 1/2; maxillary palpomere 3 slightly longer than 4. Prosternum flat to broadly and weakly convex. Elytra without sutural striae, with ground punctures usually shallowly marked; usually at least one row of systematic punctures visible along midline of each elytron (e.g., Figs 7C, 14D, 24C, D), often with visible rows along lateral and posterior regions of elytra; serial punctures sometimes visible along posterior 1/2 of elytra. Posterior elevation of mesoventrite, usually simply bulging (Fig. 6B), sometimes bulge impressed posteriorly (Fig. 6A); bulge often extending anteriorly as low, shiny, and glabrous longitudinal ridge (Fig. 6A, B); anapleural sutures concave, separated at anterior margin by distance 0.6–0.9× the width of anterior margin of mesepisternum. Metaventrite with medial glabrous patch, sometimes very narrow and extending along entire length of metaventrite. Protibiae with spines of anterior row extremely reduced to tiny appressed denticles. Metafemora with tibial grooves well developed; hydrofuge pubescence covering basal 6/7 of anterior surface. Tarsomeres 1–4 with long, thick, and rather dense setae on ventral face, sometimes with only rows of short spines on metatarsomeres 2–4; metatarsomere 2 as long or slightly longer than 5 and as 3 and 4 combined. Fifth abdominal ventrite apically emarginate, with fringe of stout setae. Aedeagus divided (e.g., Figs 3–5); parameres separated from each other for most of their length; median lobe divided in dorsal and ventral plates; dorsal plate usually strongly sclerotized and elongated, often bifurcated, or otherwise shaped along apical region; ventral plate sometimes reduced, usually simple and of variable length; basal piece 0.3× or less than length of parameres, usually clearly noticeable; gonopore usually clearly visible.

##### Differential diagnosis.

*Novochares* includes medium sized, pale brown to nearly black species that are somewhat dorsoventrally compressed and highly polished (smooth, and often shiny) to the naked eye. Across the Americas the most similar genus is *Aulonochares* Girón & Short, 2019, from which it can be differentiated by the shape of the head [trapezoid in *Novochares* (posterior margin of clypeus nearly twice as wide as anterior margin; Fig. 6D), subquadrate in *Aulonochares* (posterior margin of clypeus only nearly 1.3× wider than anterior margin; Fig. 6C)], and the sculpture of the mentum (variously strigate in *Novochares*, punctate in *Aulonochares*). Some members of *Helochares* of the Americas may resemble *Novochares* in their external features, but the aedeagal form is completely different (tubular in *Helochares*, see fig. 7 in Short and Girón 2018; divided in *Novochares*, e.g., Figs 3–5).

##### Distribution.

**Nearctic**: U.S.A. (Florida). **Neotropical**: Argentina, Belize, Bolivia, Brazil (Amapá*, Amazonas, Espírito Santo, Goiás*, Mato Grosso, Mato Grosso do Sul, Minas Gerais, Pará*, Paraíba, Pernambuco, Piauí, Rio de Janeiro, Rondônia*, Roraima*, São Paulo), Colombia, Costa Rica, Cuba, Dominican Republic*, Ecuador, French Guiana, Guatemala, Lesser Antilles (Grenada, Guadeloupe, St. Vincent), Mexico, Panama, Paraguay, Peru*, Puerto Rico, Suriname, Trinidad and Tobago*, Uruguay, Venezuela (new country or state records indicated with an asterisk).

##### Habitat.

The genus occupies a broad range of aquatic habitats, including both lentic and lotic situations. We are not aware of any seepage specialists. The most commonly collected species (e.g., those in the *N.abbreviatus* species group) are typically associated with open swamps and marshes where they may also be attracted to lights, sometimes in large numbers. Many species are also found in forested pools and swamps that contain abundant detritus. They may also be abundant in detrital pools in drying streambeds or along the margins of slow moving or quiet streams. It is also important to note that a number of *Novochares* species can co-occur in the same habitat at the same time.

##### Immature stages.

The females of most if not all species in the genus carry their egg case around under their abdomen, a behavior also seen in other *Helochares*-group genera (such as the Neotropical *Aulonochares*, *Radicitus*, and *Sindolus*; Girón and Short 2021). Only the immature stages (eggs, larvae, pupae) of *N.pallipes* have been described to date (Fernández 1983).

###### ﻿*Novocharesabbreviatus* species group

**Figure 8.**
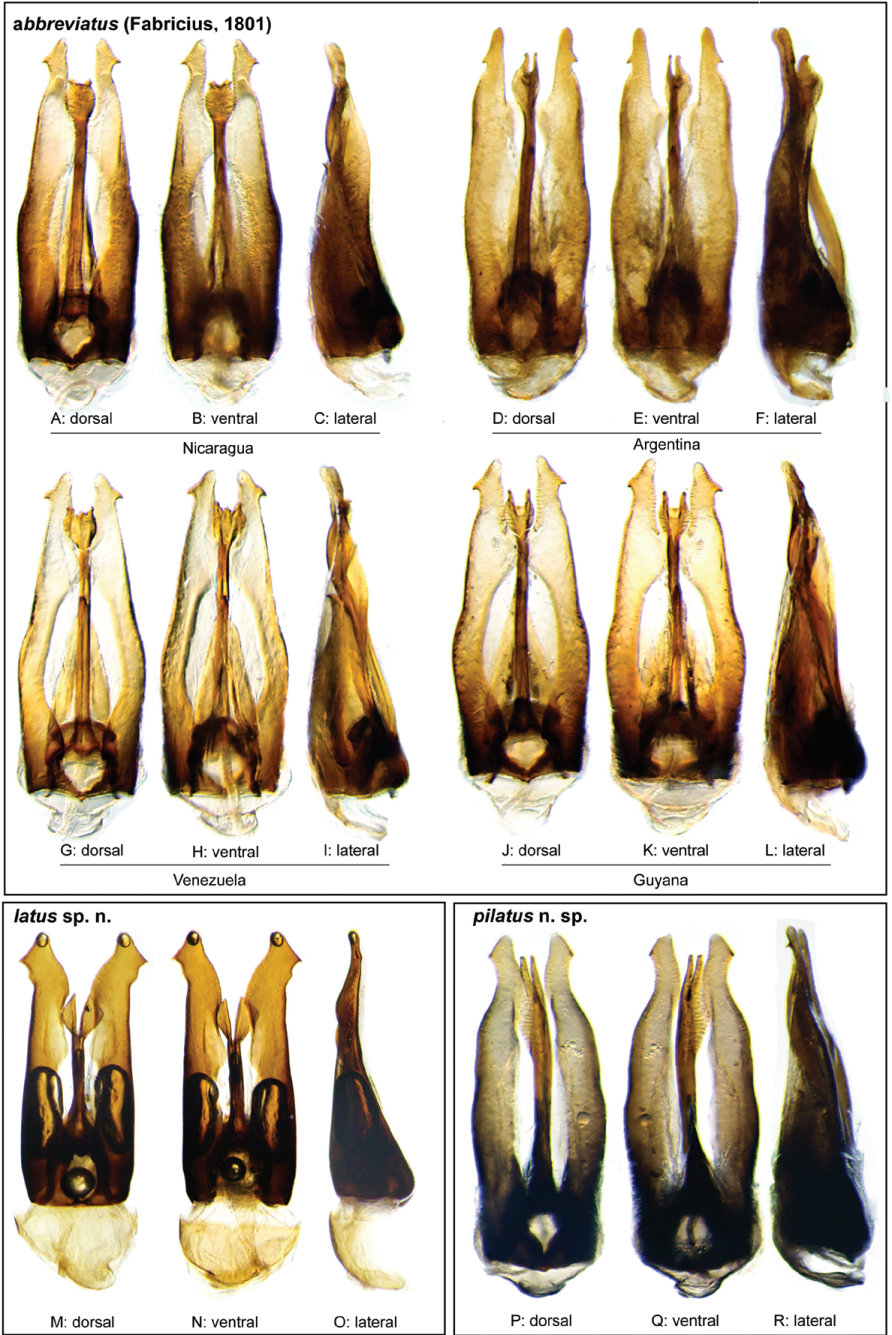
Aedeagi of the *Novocharesabbreviatus* species group **A–L***N.abbreviatus***A–C** Nicaragua **D–F** Argentina **G–I** Venezuela **J–L** Guyana **M–O***N.latus***P–R***N.pilatus***A, D, G, J, M, P** dorsal view **B, E, H, K, N, Q** ventral view **C, F, I, L, O, R** lateral view.

**Figure 9.**
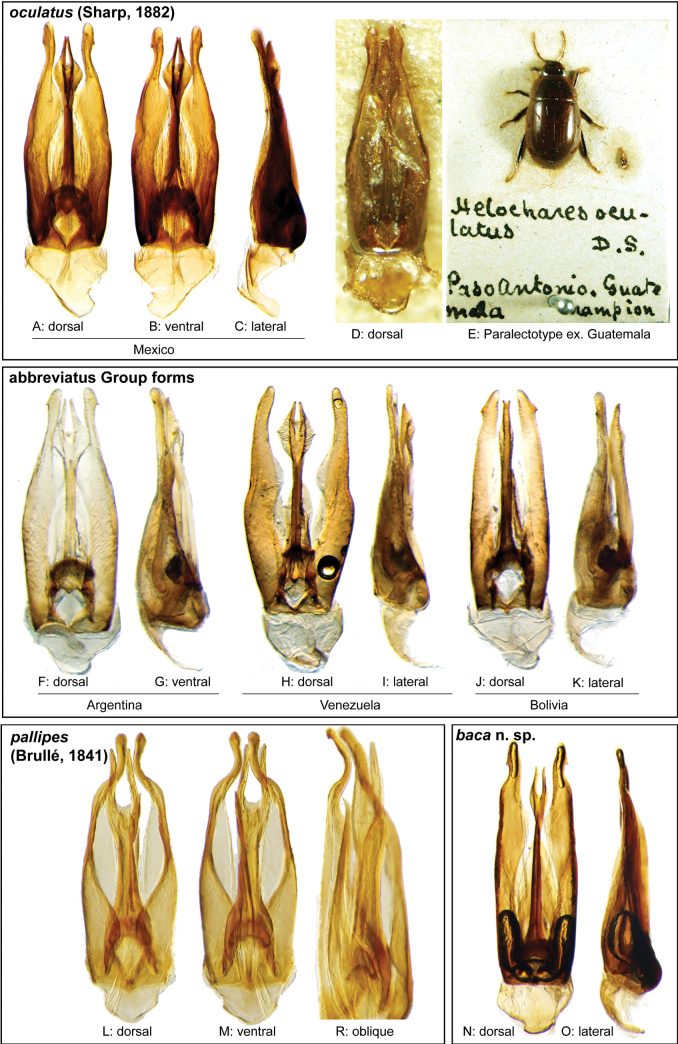
Aedeagi and habitus of the *Novocharesabbreviatus* species group **A–E***N.oculatus*: **A–C** aedeagus, Mexico **D, E** paralectotype from Guatemala **D** aedeagus **E** habitus **F–K** miscellaneous forms of aedeagi in the *abbreviatus* species group **F, G** Argentina **H, I** Venezuela **J, K** Bolivia **L, M, R***N.pallipes***N, O***N.baca***A, D, F, H, J, L, N** dorsal view **B, M** ventral view **C, G, I, K, O** lateral view **R** oblique view.

**Species group diagnosis.** Body length 5.5–8.1 mm. ***Coloration***: Dorsal surfaces pale brown to yellowish brown (Fig. 7A, B), with paler (yellowish to orange) clypeus and margins of pronotum and elytra. ***Head***: Maxillary palps 1.1–1.6× width of head, uniformly yellow or orange to brown in color (Fig. 7A, B). ***Thorax***: Ground punctation on pronotum and elytra relatively dense and very shallowly impressed. Elytra without rows of serial punctures, each with very faint rows (one dorsal and two or three lateral) of scarce and weakly marked systematic punctures. Prosternum medially weakly and broadly convex. Posterior elevation of mesoventrite broadly and somewhat triangularly elevated, sometimes posteriorly transversely impressed, with low medial longitudinal ridge extending anteriorly. ***Abdomen***: Apical emargination of fifth ventrite shallow to moderately deep and broad, U-shaped. ***Aedeagus***: (Figs 3, 8, 9) Overall shape sub-rectangular to sub-rhomboid, 2.5–3.0× longer than wide, joint basal margins of parameres truncate (e.g., Fig. 8M) to medially pointed (e.g., Fig. 8A, G); outer margin and apical region of each paramere variable, usually rounded at apex and laterally pointed; parameres longer than median lobe; parameres with apical region uniformly sclerotized and dorso-ventrally flattened; dorsal inner margin of each paramere usually medially broadly emarginate (see Fig. 5 in blue); dorsal plate of median lobe (in dorsal view) with basal apodemes usually reaching base of parameres; dorsal plate of median lobe usually with long and narrow neck (see Fig. 5 in yellow), variably expanded at apical region, usually with distal arms variable in shape and length; gonopore sitting proximal to base of median lobe; ventral plate of median lobe (in ventral view, see Fig. 5 in red) somewhat triangular, moderately sclerotized; dorsal surface of ventral plate of median lobe slightly concave (lateral regions curved dorsally); basal piece nearly (see Fig. 5 in green) 0.25–0.30× length of a paramere. In lateral view, aedeagus triangular, nearly straight at base, with ventral outline of parameres 3.0–3.6× longer than greatest width near base (e.g., Fig. 8C, F, I, L, O).

**Composition.** This species group contains three previously described species: *Novocharesabbreviatus* (Fabricius, 1801), *N.oculatus* (Sharp, 1882), and *N.pallipes* (Brullé, 1841) and three new species: *N.baca* sp. nov., *N.latus* sp. nov., and *N.pilatus* sp. nov.

**Remarks.** This is a widespread and certainly problematic species group. The external morphology of all the known species is annoyingly uniform, and the characteristics of the male genitalia, especially the apical region of the dorsal plate of the median lobe, when looking at long series of specimens from across the distributional ranges of *N.abbreviatus* and *N.oculatus*, exhibit gradual changes that blur the limits between both species. More thorough sampling and molecular data, perhaps in the context of international collaboration across the species group range, are needed to tackle the systematics and taxonomy of this species group.

###### ﻿The taxonomy and nomenclature of the *N.abbreviatus* group: the past

Fabricius (1801) described *Hydrophilusabbreviatus* (now *N.abbreviatus*) from “America meridonali”, the oldest species of what is now included in *Novochares*. Brullé (1841) described *Philhydruspallipes* (now *N.pallipes*) from Uruguay. Later, Sharp (1882) described *Helocharesoculatus* from Guatemala. The descriptions of all three of these species are very brief and no consideration is given to the aedeagus (as was typical at the time). Following the publication of these descriptions, there does not seem to be any question that subsequent authors considered them to be very closely related species, if not all actually conspecific.

d’Orchymont (1926) considered *Helocharesoculatus* Sharp, 1882 as a synonym of *Philhydruspallidus* Castelnau, 1840, citing mostly similarities in dorsal coloration and elytral punctation. Ten years later, d’Orchymont (1936) examined a unique female specimen from the Schestedt-Lund collection, labeled as “*H.abbreviatus* ex. Ins. Amer.” which he treated as the Fabrician type specimen, and attributed the locality to be in the Antilles (“il est donc des Antilles”; d’Orchymont 1936: 10). The existence of a single Fabrician specimen is corroborated by Zimsen (1964: 66), who also lists a single specimen housed in the Copenhagen collection, and by Spangler (1981) who also examined it himself. After examining this type, d’Orchymont (1936) considered *Philhydruspallidus* Castelnau, 1840 as a synonym of *H.abbreviatus*, though he indicates this interpretation is based on a specimen of *P.pallidus* determined by Régimbart. Because d’Orchymont had already synonymized *H.oculatus* Sharp with *H.pallidus*, *H.oculatus* now became a synonym of *H.abbreviatus*. It is worth noting that the Castelnau collection is considered lost and possibly destroyed (Evenhuis 2012), so the original identity of *H.pallidus* will likely never be known.

A few years later, d’Orchymont (1939) revisited the identity of *H.abbreviatus* after having examined the aedeagus in a variety of specimens. He noticed some male specimens from Mexico, Panama, and Brazil (Mato Grosso do Sul) have a larger tip of the dorsal plate of the median lobe and less acute teeth on the paramere apices (he illustrated both forms; fig. 4 in d’Orchymont 1939). He speculated that these specimens with the larger, broader apex of the dorsal plate of the median lobe may be the *H.oculatus* of Sharp. He suggests that the dissection of Sharp’s type specimens should confirm his hypothesis, though at that time it was not known if the types were male or female. It is also of note that d’Orchymont observed that both “forms” of the genitalia (i.e., both the *abbreviatus* form and the *oculatus* form) co-occur at a variety of localities. d’Orchymont (1939: 259) goes on to comment on the situation of the identity of *H.abbreviatus* (translated from French): “To justify these attributions [of the names *H.abbreviatus* and *H.oculatus*] let us recall that there is a doubt as to the origin (South America or the Antilles?) of the type of Fabricius and that it is a female. There is therefore no inconvenience in considering definitively as typical the most widespread form in South America, like the one showing the aedeagus of figure 4.” [which illustrates the modern concept of *H.abbreviatus*]

The same year that d’Orchymont was lamenting the confusion around *H.abbreviatus*, Balfour-Browne (1939) described *Helocharesrufobrunneus* from the Antilles, specifically from Grenada and St. Vincent. He said that his new species was “very similar to *H.abbreviatus* in color” but can be separated by the elytral punctation and a few other minor characters. Although he illustrates the aedeagus of *H.rufobrunneus*, which is a very good match to the “true” *H.abbreviatus* of d’Orchymont (1939), he remarks that the genitalia of the two species are “quite distinct” and refers to the apex of the median lobe being deeply forked and expanded in *H.abbreviatus*. Based on this later comment about the aedeagus, we believe that what Balfour-Browne considered to be *H.abbreviatus* was in fact *H.oculatus*, and therefore he inadvertently redescribed *H.abbreviatus*.

While summarizing aquatic beetles from Cuba, Spangler (1981) provided a detailed narrative on the status of *H.abbreviatus* and *H.rufobrunneus*, as he had examined the types of both species. He pointed out that the attribution of the type specimen of *H.abbreviatus* coming from the Antilles comes from details provided by Zimsen (1964). Specifically, that Smidt (the provider of the type specimen of *H.abbreviatus* to Fabricius) was a customhouse officer in St. Croix who collected insects while stationed there. He considered this to be important because as the Fabricius type was female, in theory it could represent a senior synonym of *H.oculatus*, further confounding the names. However, he had not seen any specimens from the Caribbean Islands with the *H.oculatus* form of the genitalia, and so concluded this was improbable. He concluded that *H.rufobrunneus* was a junior synonym of *H.abbreviatus*.

Subsequently, Fernández (1982a) confirmed that indeed the aedeagus of a syntype of *H.oculatus* matched the form that d’Orchymont (1939) had speculated was the true *H.oculatus* (see. Fig. 9D, E). She designated a lectotype, removed *H.oculatus* from synonymy with *H.abbreviatus*, and reinstated it as a distinct species. Unaware that Spangler had synonymized *H.rufobrunneus* with *H.abbreviatus* the year before, she also independently concluded that these two species were synonyms after examining the type of the former and proposed their synonymy.

The identity of the only other previously described species in this group, *N.pallipes* (Brullé), has been much less controversial. Its modern identity has been constant since d’Orchymont (1939, fig. 3) examined the type specimen and illustrated the genitalia, which is fairly distinct by *Novochares* standards.

###### ﻿The taxonomy and nomenclature of the *N.abbreviatus* species group: the present and future

Soon after we began working on this revision, it became clear that this group of species would be particularly difficult to resolve–something that was not at all a surprise given the confusion it has posed in the past. Many of the challenges in this group are not unique within *Novochares*: The lack of external characters, the presence of extremely widespread species, and subtle variation in aedeagal forms are found in many species groups within the genus. However, each of these problems is extreme within the *abbreviatus* species group, combined with the fact that this is by far the most commonly found species group in collections. At least one species of *Novochares* probably inhabits most open marshlands and pond margin habitats from Mexico to Argentina. Despite being only a few of more than 50 species, it probably represents more than half of the specimens in collections, and in Mesoamerica and the Caribbean Islands, the overwhelming majority of specimens we observed are in this group.

We found there to be many subtle forms of variation in the aedeagus, much more so than we observed in other species groups. When we applied molecular data to try to resolve this issue, we found that *Novocharesabbreviatus* truly is a very widespread species, with specimens from disparate localities such as Costa Rica and southern Brazil neatly grouping together, and that there are many distinct genetic lineages even within our very modest sampling (given the range and commonality of the group; see Fig. 2) and the differences in the aedeagal morphology can be small (Figs 8, 9). Despite all the material and data at our disposal, there remained uncertainty about the boundaries between some of these lineages/putative species, and if we were not confident in telling them apart, we know that others would struggle even more. We were able to generally circumscribe the previously named species and have chosen to describe three relatively distinct lineages as new species that are supported by both morphology and DNA data.

#### ﻿ Novochares abbreviatus

Taxon classificationAnimaliaColeopteraHydrophilidae

(Fabricius, 1801)

DC80CB0C-D118-55FA-BFC3-D3D647E97124

**Figure 10.**
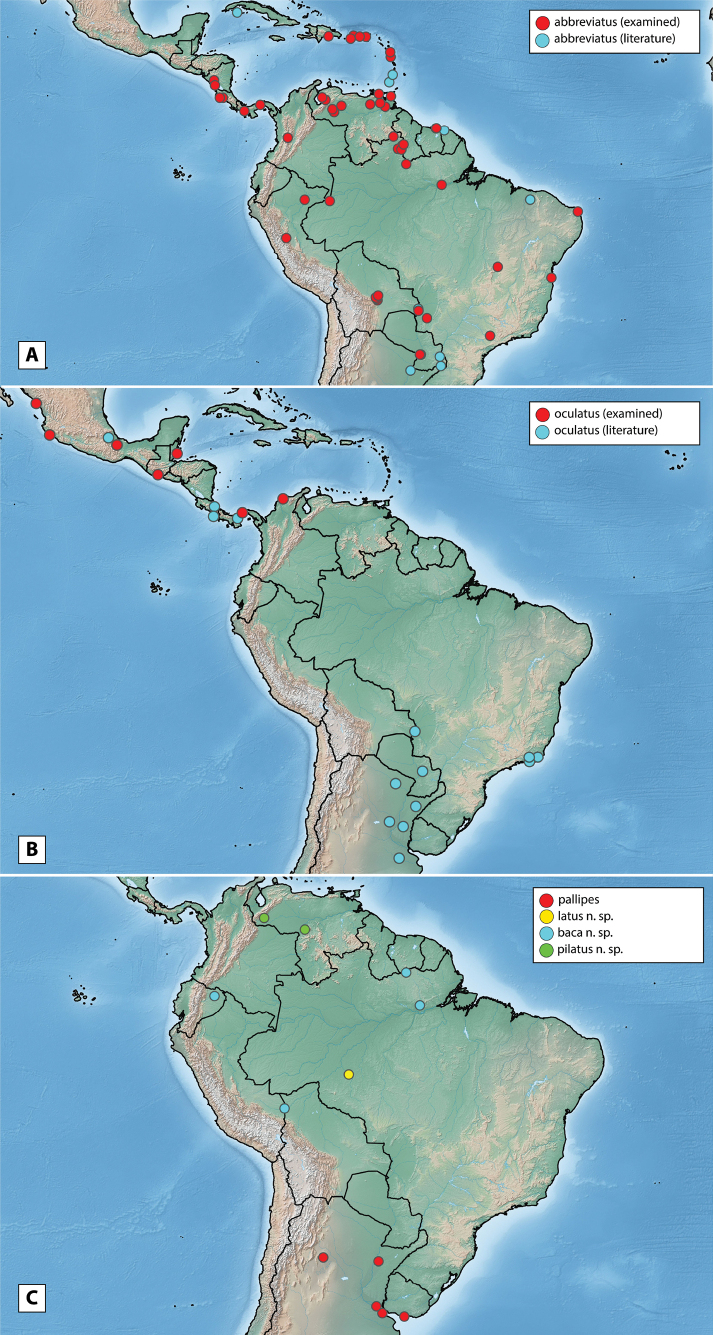
Distribution of *Novocharesabbreviatus* species group **A***N.abbreviatus*: examined specimens (red) and literature/unconfirmed records (blue) **B***N.oculatus*: examined specimens (red) and literature/unconfirmed records (blue) **C***N.pallipes* (red), *N.latus* (yellow), *N.baca* (blue), *N.pilatus* (green).

Figs 2 , 3A , 7A , 8A–L , 10A

Hydrophilus abbreviatus Fabricius, 1801: 251 - [America meridionali]; Zimsen 1964: 66 [lists type specimens].
Helochares (s. str.) abbreviatus (Fabricius, 1801); d’Orchymont 1939: 258 [taxonomic treatment]; d’Orchymont 1943: 55 [faunistic treatment]; Fernández 1982a: 32 [taxonomic treatment]; Fernández 1989: 148 [in key]; Hansen 1999: 159 [catalog]; Short 2005: 215 [new record];Clarkson and Ferreira Jr. 2014 : 400 [faunistic treatment]; Silva et al. 2018: 9 [faunistic treatment].
Helochares abbreviatus (Fabricius, 1801); Gonzalez-Rodriguez et al. 2017: 606 [checklist].
Philhydrus pallidus Castelnau, 1840: 53 - Brazil (secondary homonym of Hydrophiluspallidus Rossi, 1792); d’Orchymont 1936: 10 [synonymy].
Philhydrus pallidus Castelnau, 1840; Gemminger and Harold 1868: 482 [checklist].
Helochares pallidus (Castelnau, 1840); Fleutiaux and Sallé 1889: 376 [checklist].
Enochrus (Lumetus) pallidus (Castelnau, 1840); Zaitzev 1908: 388 [checklist].
Helochares (Hydrobaticus) rufobrunneus Balfour-Browne, 1939: 293. - Lesser Antilles, Grenada, Balthazar; Spangler 1981: 158 [synonymy].
Novochares abbreviatus (Fabricius, 1801); Girón and Short 2021: 203.

##### Material examined

**(98 exs.). Bolivia: Santa Cruz**: Ayacucho, 13–14.v.1969 leg. P. & P. Spangler (1, USNM); Santa Cruz, 11–12.v.1969, leg. P. & P. Spangler (1, USNM); 60 mi N. Santa Cruz, Saavedra Experiment Station, 3–5.i.1960, leg. Robert Cumming (1, USNM); Potrerillos del Guenda, Preserva Natura, 17°40'S, 63°27'W, 370 m, 17–22.x.2007, lights, leg. Cline & Wappes, BOL1Cline07 007 (1, SEMC); 3.7 km SSE Buena Vista, Hotel Flora y Fauna, 23–30.iv.2004, lights, leg. A.R. Cline (4, SEMC, TTU-Z), same data but 1–12.v.2004 (4, SEMC). **Brazil: Bahia**: 15 km E. Itabuna, 3.vii.1963, leg. P. & P. Spangler (2, USNM). **Goiás**: Divinópolis de Goiás, 2 km SE on GO-447, Roadside ponds, BR18-0222-02A (1, SEMC, DNA voucher, SLE2087). **Pará**: Murumuru, 1.5 km E, Muddy shallow marsh along road, BR18-0205-02A (1, SEMC, DNA voucher SLE 2083). **Mato Grosso do Sul**: Corumba (ca. 29 km SE) on BR-262, 25.vi.2018, leg. Hamada et al., drying marsh, BR18-0625-02A (1, SEMC); Miranda (ca. 9.5 km SW) on MS-339, Marsh area alongside stream, BR18-0626-03A (2, SEMC, including DNA voucher SLE2086). **Rio Grande do Norte**: Ceara-Mirim, 6–7.vii.1969, leg. P. & P. Spangler (1, USNM). **Roraima**: BR-401, ca. 6 km SW of Bonfim, 3°21.615'N, 59°53.361'W, 100 m, 12.i.2018, leg. Short, Benetti & Santana, large marsh with abundant vegetation, BR18-0112-02A (1, SEMC); BR-401, ca. 26 km NE of Boa Vista, 2°56.191'N, 60°28.017'W, 92 m, 12.i.2018, leg. Short, pooled up morichal, BR18-0112-06B (1, SEMC); Caroebe, Rio Jatapu, nr. Usina de Jatapu, 00°50.939'N, 59°18.262'W, 145 m, 17.i.2018, leg. A. Short, marginal pools of river, BR18-0117-02A (1, SEMC). **São Paulo**: Piracicaba, 12.xii.1965, leg. C.A. Triplehorn (1 male, USNM); same data but 6.x.1965 (1, USNM). **Colombia: Amazonas**: Leticia, 12–15.iii.1969, leg. P. & P. Spangler (1, USNM). **Costa Rica: Guanacaste**: nr. Carmona, laguna de crocodile, 34 m, 16.i.2003, leg. Short, Roughly, & Porras, HG light (3, SEMC); Rio Animas, River w/ volcanic rock bottom, small cascades, small isolated detrital backwaters, AS-04-040 (1, SEMC, DNA Voucher SLE1180). **Puntarenas**: Puntarenas, 22.vii.1965, leg. P.J. Spangler (1, USNM). **Dominica**: Cabrit Swamp, 10–13.v.1965, leg. D.R. Davis (1, USNM); Postsmouth, 19–21.x.1966 (1, USNM). **Dominican Republic**: La Toma N of San Cristobal, 9–10.vi.1969, leg. Flint & Gomez (1, USNM). **Guadeloupe**: Pointe-a-Pitre, 1936, Henri Stehle (1, USNM). **Guyana**: Good Hope (7 mi. NW, on road to Karasabai), 26.iv.1995, leg. Spangler & Perry (1, USNM); **Region 9**: Tributary of the Takatu River, NW of Kusad Mts., 2°50.563'N, 59°59.113'W, 109 m, 24.x.2013, leg. Short, Isaacs, & Salisbury, vegetated creek margins, GY13-1024-02B (1, CBDG); nr. Kusad Mts., 2°49.793'N, 59°48.361'W, 123 m, 25.x.2013, leg. Short, Isaacs, & Salisbury, large vegetated marsh, GY13-1025-01A (1, SEMC, DNA Voucher SLE1221); Pirara Ranch & River, 3°32.1'N, 59°40.5'W, 23–27.iv.1995, leg. O.S. Flint (1, USNM). **Nicaragua**: 13 mi N. Sn. Benito, 11.vii.1965, leg. P.J. Spangler (1, USNM); Rivas, Reserva Silvestri Domitila, 5–9.vi.2005, 400 ft., lights, W.D. Shepard (2, SEMC). **Panama: Chiriqui Province**, Las Lajas (8 km S.), 10 m, 5.vi.1983, leg. P.J. Spangler, roadside ditch (1, USNM). **Panama Province**: Pond at Panama Canal, Explosive Depot, 31.viii.2006, leg. W.D. Shepard & D. Post (3, SEMC). **Paraguay: Central Department**: San Bernardino, 10.iv.1980, leg. Spangler, Culzoni, & Wood (1, USNM); same locality but 22.vi.1969, leg. P. & P. Spangler; Aregua, 26–27.iv.1980, leg. P.J. Spangler (1, USNM). **Peru: Huanuco**: Tingo María, 19–24.iv.1969, leg. P. & P. Spangler (1, USNM); **Loreto**: SW Iquitos, Aguajal next to Iquitos-Nauta highway, Margins and saturated leaves of aguajal (palm swamp) PE20-0119-01A (1, SEMC, DNA voucher SLE2152). **Puerto Rico**: San Germán, 23.xii.1962, leg. P. & P Spangler (1, USNM); nr. La Cueva del Indio, 14.i.1963, leg. P.J. Spangler (1, USNM); Hwy 3, km 32.6 nr. Palmer, 10.i.1963 (1, USNM). **Suriname: Commewinje**: East-West Highway, ca. 6 km E. of Suriname River, 5°47.464’N, 55°06.730’W, leg. Short, SR13-0809-01A (3, NSCS, SEMC). **Marowijne**: East-West Highway, 15 Km E. Commewijne River, 4.iii.2012, leg. Short & Kadosoe, sandy/marshy roadside swale, SR12-0304-04 (1, SEMC, DNA voucher SLE1210). **Saramacca**: 1 km E. Sidiredjo, 5.iii.2012, leg. Short & Kadosoe, roadside swale, SR12-0305-02A (1, SEMC, DNA voucher 1215). **Trinidad And Tobago**: Trinidad, Debe, 17.vii.1969, leg. P. & P. Spangler (1, USNM). **Venezuela: Anzoátegui**: El Tigre, N of, river along highway, 9°5.808'N, 64°19.445'W, 236 m, 3.ii.2010, leg. García, shaded margins without vegetation, VZ10-0203-03A (1, SEMC); same data except leg. Short, vegetated backwater margins, VZ10-0203-03B (1, SEMC). **Apure**: Bruzual, edge of town, 8°2.534’N, 69°20.530’W, 83 m, 18.i.2009, leg. Short, Camacho, Miller, large marsh VZ09-0118-04X (1, SEMC). **Barinas**: Obispo, 25.ii.1969, leg. P. & P. Spangler (1, USNM); Libertad, E of, along side gravel road, 8°25.773’N, 69°35.202’W, 106 m, 19.i.2009, leg. Short, Camacho, Miller, forested canal, VZ09-0119-01X (2, SEMC); Ciudad Bolivia, approx. 13 km SE, large Hacienda, 8°19.394'N, 70°28.238'W, 173 m, 25.i.2012, leg. Short, Arias, & Gustafson, marsh, VZ12-0125-02A (1, SEMC, DNA voucher SLE1217). **Bolívar**: Gran Sabana, N. Santa Elena, Rio Guara at Rt. 10, 4°37.362’N, 61°5.679’W, 876 m, 17.vii.2010, leg. Short, Tellez, & Arias, marshy area, VZ10-0717-02A (2, SEMC). **Cojedes**: El Baul, 5 km S, 21.i.2012, leg. Short, Arias, & Gustafson, large marsh, VZ12-0121-03A (1, SEMC, DNA Voucher SLE1240). **Delta Amacuro**: Between Tucupita & Los Guires, 9°10.504’N, 61°54.610’W, 8 m, 3.ii.2010, leg. Short & García, marsh by road, VZ10-0203-01A (1, SEMC, DNA Voucher SLE1208); between Tucupita & Temblador, small pond along road, 8°46.439'N, 62°14.306'W, 19 m, 2.ii.2010, leg. Short, García, Joly, margins of vegetated pond, VZ10-0203-02A (1, SEMC). **Guárico**: San Fernando, 12.ii1969, leg. P.&P. Spangler (1, USNM). **Monagas**: S of Maturin, morichal at road crossing, 9°16.398'N, 62°56.246'W, 22 m, 2.ii.2010, leg. Short, García, & Joly, morichal margin, VZ10-0202-02A (2, SEMC). **Sucre**: El Pilar, approx. 5 km SE, 10°31.419'N, 63°7.070'W, 2 m, 29.i.2010, leg. Short & Garcia, marsh/swamp along road, VZ10-0129-04A (17, MIZA, SEMC, TTU-Z, including DNA voucher SLE1207). **Trujillo**: Sabana Grande, Rio Jirijara, 9°42.307'N, 70°32.570'W, 199 m, 29.i.2012, leg. Short, Arias, Gustafson, small muddy pool in river floodplain, VZ12-0129-02A (1, SEMC). **Zulia**: Puente del Zulia, lagoon on finca, 17.i.2012, leg. Short et al., large lagoon, V12-0127-01A (1, SEMC, DNA voucher SLE1237); Sabana de Machango, 10°2.581'N, 71°0.428'W, 35 m, 29.i.2012, leg. Short, Arias, & Gustafson, margin of artificial pond, VZ12-0129-03A (1, SEMC). **Virgin Islands**: St. Thomas, 20.i.1963, leg. P.J. Spangler (2, USNM).

##### Differential diagnosis.

The defining feature of this species is the shape of the apical region of the dorsal plate of the median lobe, which bears a small rounded “cup” at its apex with two or three small teeth along its distal margin (Fig. 8A–L). However, only specimens that very closely match the illustrated forms should be considered *N.abbreviatus*. Specimens that possess expanded apical “cups” or long distal arms are likely other species such as *N.oculatus* (Fig. 9A, B), *N.baca* (Fig. 9N), or other still undefined lineages.

##### Description.

Body length 5.5–7.0 mm. ***Coloration***: Dorsal surfaces pale brown to yellowish brown, with paler (yellowish) clypeus and margins of pronotum and elytra (Fig. 7A). ***Head***: Maxillary palps 1.2–1.6× width of head, uniformly orange to brown in color (Fig. 7A). ***Thorax***: Elytra without rows of serial punctures, each with very faint rows (one dorsal and two or three lateral) of scarce and weakly marked systematic punctures. Prosternum medially weakly and broadly convex. Posterior elevation of mesoventrite broadly and somewhat triangularly elevated, sometimes posteriorly transversely impressed, with low medial longitudinal ridge extending anteriorly. ***Abdomen***: Apical emargination of fifth ventrite shallow to moderately deep and broad. ***Aedeagus***: (Figs 3A, 8A–L) Outer margin and apical region of each paramere rounded at apex and laterally pointed to hook-shaped; dorsal plate of median lobe (in dorsal view) with long and narrow neck, ovally expanded at apical region, with two or three very short distal arms; ventral plate of median lobe triangular, strongly sclerotized.

##### Distribution.

Argentina, Bolivia, Brazil (Espírito Santo, Mato Grosso do Sul, Pará, Pernambuco, Piauí, Rio Grande do Norte, Roraima, São Paulo), Colombia, Costa Rica, Cuba, Dominica (new record), Dominican Republic (new record), French Guiana, Guadeloupe (new record), Guyana (new record), Nicaragua (new record), Panama, Paraguay, Peru (new record), Puerto Rico (new record), Suriname, St. Thomas (new record), Trinidad and Tobago (new record), Venezuela (Fig. 10A).

##### Habitat.

This species is a common element of the lentic water beetle fauna throughout much of the Neotropics. It is most frequently collected in open marshes, swamps, pond margins, or along the margins of larger rivers.

##### Remarks.

The body length measurements presented here correspond to confirmed males for the species. See extensive discussion and remarks under the species group for further history and information that relates to this name and taxon.

Among the thirteen specimens we sequenced from Costa Rica to southern Brazil, the maximum intraspecific pairwise divergence in COI was a relatively meager 3.7%, and along with relatively uniform morphology, this supports the conclusion that this species is extremely widespread throughout the Neotropical region as the literature suggests.

#### ﻿ Novochares baca sp. nov.

Taxon classificationAnimaliaColeopteraHydrophilidae

0340522E-D59D-52BF-86A9-E8CBC1FAD742

https://zoobank.org/F8E6139D-B193-4397-8E45-5C9605E0975D

Figs 9N, O , 10C

##### Type material.

***Holotype* (male)**: “PERU: Madre de Dios: Tambopata/ -12.54034 S, -69.00074 W, 190m/ Kawsay Biological Station, 4.vi.2022/ Palm swamp; lots of detritus/ PE22-0604-01B, leg. Short et al.” (MHNSM). ***Paratypes* (9 exs.): Brazil: Pará**: ca. 25 km E of Alenquer, -1.96253, -54.50458, 44 m, 4.ii.2018, Short & Benetti, Palm swamp, lots of detritus, BR18-0204-01A (1, SEMC, DNA Voucher SLE1513); Vale do Paraíso, ca. 55 km N. Alenquer, -1.49292, -54.51566, 150 m, leg. Short & Benetti, seeps & pools by waterfall, BR18-0203-01D (1, INPA, DNA Voucher SLE1617); same data except pool with rocks and detritus on trail, BR18-0203-01E (1, SEMC, DNA Voucher SLE2081). **Ecuador: Sucumbíos**: Sacha Lodge, 0.5°S, 76.5°W, 270 m, 10–12.xi.1994, leg. Hibbs, ex. Malaise (1, SEMC). **Peru: Madre de Dios**: Same data as holotype (4, SEMC, MHNSM). **Suriname: Sipaliwini**: Sipaliwini Savannah Nature Reserve, North of Basecamp, 2°00.656'N, 55°59.070'W, 275 m, 1.iv.2017, leg. Short, grassy pools near river, SR17-0401-01A (1, NZCS).

##### Differential diagnosis.

The relatively long and straight outer margins of the parameres and the very long and narrow fork of the apex of the dorsal plate of the median lobe (Fig. 9N) help to distinguish this species from others in the species group.

##### Description.

Body length 6.0–6.2 mm. ***Coloration***: Dorsal surfaces pale brown to yellowish brown, with paler (yellowish) clypeus and margins of pronotum and elytra. ***Head***: Maxillary palps only slightly longer than width of head, uniformly yellow in color. ***Thorax***: Elytra without rows of serial punctures, each with very faint rows (one dorsal and two or three lateral) of scarce and weakly marked systematic punctures. Posterior elevation of mesoventrite broadly and somewhat triangularly elevated, weakly posteriorly transversely impressed, with low medial longitudinal ridge extending anteriorly. ***Abdomen***: Apical emargination of fifth ventrite moderately deep and broad. ***Aedeagus***: (Fig. 9N, O) Outer margin and apical region of each paramere rounded at apex and laterally pointed; dorsal plate of median lobe with long and narrow neck, ovally expanded at apical region, with two narrow, elongated, laminate (dorsoventrally oriented), distal arms, basally separated by width of arm; arms as long as base of fork; ventral plate of median lobe triangular, strongly sclerotized, apically broadly angulate, reaching mid-length of dorsal plate.

##### Etymology.

This species is named after Stephen Baca, long-time member of the Short Lab, who supported the lab and its members by providing assistance during fieldwork, lab work, and overall being a great friend, colleague, and mentor. The last name Baca, when read, sounds similar to the Spanish word *vaca*, cow in English; the distal region of the dorsal plate of the median lobe somewhat resembles the head of a cow with horns.

##### Distribution.

Known from a few but widely separated localities in Brazil (Pará), Ecuador, Peru, and Suriname (Fig. 10C).

##### Habitat.

This species has been collected in palm swamps, forested pools alongside rivers and waterfalls, and in the case of the Suriname locality, in a grassy pool alongside a river in an open savanna.

#### ﻿ Novochares latus sp. nov.

Taxon classificationAnimaliaColeopteraHydrophilidae

45C6EDA8-18D0-53B4-B776-53D8AD607D76

https://zoobank.org/46DBC9A5-59AE-4EFF-B673-CB36732AA3AE

Figs 8M–O , 10C

##### Type material.

***Holotype* (male)**: “BRAZIL: Rondônia/ -8.92368, -62.12491; 82 m/ Tabajara (c. 7.5 km W) on RO-133/ 8.vii.2018. leg. Short; river/ w/sandy bottom and rocks/ BR18-0708-04A”, “DNA Voucher/ Extraction #/ SLE-2039” (INPA). ***Paratypes*: (4 exs.): Brazil: Rondônia**: same data as holotype (4, INPA, SEMC).

##### Differential diagnosis.

This taxon has one of the most distinctive aedeagal forms within the species group. The extremely broad, parallel sided parameres with a ‘birdhead’ form at the apex, paired with a uniquely short and deeply cleft apical region of the dorsal plate of the median lobe (Fig. 8M–O) make this species easy to recognize; we did not see any other variations that were similar to this one.

##### Description.

Body length 7.2–8.1 mm. ***Coloration***: Dorsal surfaces pale brown, sometimes with weakly paler (yellowish) clypeus and margins of pronotum and elytra. ***Head***: Maxillary palps nearly 1.3× width of head, uniformly yellow to orange in color. ***Thorax***: Elytra without rows of serial punctures, each with very faint rows (one dorsal and two or three lateral) of scarce and weakly marked systematic punctures. Prosternum medially broadly convex. Posterior elevation of mesoventrite broadly and somewhat triangularly elevated, transversely weakly impressed posteriorly, with low medial longitudinal ridge extending anteriorly. ***Abdomen***: Apical emargination of fifth ventrite moderately deep and relatively narrow. ***Aedeagus***: (Fig. 8M–O) Overall shape sub-rectangular; apical region of each paramere relatively broad, rounded at apex, with outer margin laterally pointed; dorsal plate of median lobe with long and narrow neck, bifurcated at apical region; each arm of the fork somewhat triangular, relatively broad, and short; ventral plate of median lobe triangular, strongly sclerotized, reaching to second 1/3 of dorsal plate.

##### Etymology.

*Latus* (L.) meaning broad, referring to the shape of each arm of the apical region of the dorsal plate of the median lobe.

##### Distribution.

Only known from the type locality in Brazil (Fig. 10C).

##### Habitat.

The single collection of this species was from the sandy margin of a forested creek with some detritus.

#### ﻿ Novochares oculatus

Taxon classificationAnimaliaColeopteraHydrophilidae

(Sharp, 1882)

3A838842-620D-58D3-BDA9-4E5F64A8D010

Figs 3B , 9A–E , 10B

Helochares oculatus Sharp, 1882: 74.
Helochares (s. str.) oculatus Sharp, 1882; Fernández 1982a: 31 [specific rank confirmed; not syn. of pallidus Castelnau, as in d’Orchymont 1926: 232; not. syn. of abbreviatus Fabricius, as in d’Orchymont 1936: 10; lectotype designated]; Fernández 1989: 148 [in key]; Hansen 1999: 162 [catalog].
Novochares oculatus (Sharp, 1882); Girón and Short 2021: 205.

##### Type material examined.

***Paralectotype* (male)**: “Helochares ocu-/latus D.S./ Paso Antonio. Guate/mala Champion [on card with specimen]”, “Sharp Coll./ 1905.-313”, “Paso Antonio,/ 400 ft./ Champion.”, “B.C.A. Col. I. 2./ Helochares/ oculatus,/ Sharp.”, “Brit.Mus./ USNM-1966/ EXCHANGE” (USNM; Fig. 9E).

##### Additional material examined

**(8 exs.). Belize**: Stann Creek, Sitte Point, Possum Point Biological Station, 24.iv.1987, leg. P.J. Spangler (1, USNM). **Colombia: Magdalena**: 8 km E Barranquilla, 19.iii.1969, leg. P. & P. Spangler (1, USNM). **Mexico: Jalisco**: Barra de Navidad, 23.iii.1971, leg. J.R. Zimmerman (1, USNM). **Oaxaca**: 31 km S Tuxtepec, Bethania, Ao. Chopan, 24.v.1981, blacklight, leg. P.J. Spangler (1, USNM); **Sinaloa**: Mazatlan, 17.vii.1963, leg. P.J. Spangler (1, USNM). **Panama**: Canal Zone, Barro Colorado Island, vi.1939, leg. J. Zetek (2, USNM), same locality but 29.v.1940, at light (1, USNM).

##### Differential diagnosis.

The precise morphological boundaries of this taxon are still uncertain. Its primary characteristic within the species group is its relatively large and oval distal cup of the dorsal plate of the median lobe, which bears two long arms (Fig. 9A, B). However, there is much variation and we recommend a relatively conservative approach in making confirmed species identifications at this time (see remarks for further discussion).

##### Description.

Body length 6.0–6.5 mm. ***Coloration***: Dorsal surfaces pale brown to orange-brown, sometimes with weakly paler (orange) clypeus and margins of pronotum and elytra. ***Head***: Maxillary palps nearly 1.3× width of head, uniformly yellow to orange in color. ***Thorax***: Elytra without rows of serial punctures, faint rows (one dorsal and two or three lateral) of scarce and weakly marked systematic punctures barely noticeable. Prosternum medially broadly convex. Posterior elevation of mesoventrite broadly elevated, with low medial longitudinal ridge extending anteriorly. ***Abdomen***: Apical emargination of fifth ventrite moderately deep and relatively narrow. ***Aedeagus***: (Figs 3B, 9A–D) Outer margin and apical region of each paramere rounded at apex and laterally pointed to hook-shaped; dorsal plate of median lobe with long and narrow neck, obliquely explanate at apical region, somewhat diamond-shaped and laterally rounded, with two laminate and slender distal arms, narrowly separated at base, apically converging, and slightly dorsally pointing; arms nearly as long as base of fork; ventral plate of median lobe triangular, moderately sclerotized.

##### Distribution.

Belize (new record), Colombia (new record), Costa Rica, Guatemala, Mexico (confirmed record), Panama (Fig. 10B). All records from southern South America and the Caribbean need further review (Argentina, Brazil, Paraguay, and the Antilles [Grenada, St. Vincent]).

##### Remarks.

The identity of this species has been wrapped up in confusion with *N.abbreviatus* almost immediately after it was described (see above discussion of the *abbreviatus* group nomenclature). We examined the dissected male lectotype and a dissected male paralectotype male deposited in the USNM (Fig. 9D, E; it had been exchanged between USNM and the British Museum in the 1960s). We saw a number of other specimens that had an *oculatus*-like aedeagus but with a range of variation around the precise shape and size of the apical region of the dorsal plate of the median lobe, as well as the shape of the paramere apices (Fig. 9F, H). We did not have good sampling of these forms for molecular data, so were not able to use an integrated approach to explore this variation. Consequently, we have taken a very conservative approach and identified only male specimens which had genitalia that very closely matched the type material as *N.oculatus*. The remaining material will need to wait until more data, especially genetic data, is available. There are a number of published records of *N.oculatus* from Argentina, Paraguay, and southern Brazil (Fernández 1982a; Clarkson and Ferreira Jr 2014), and while it is very much possible that *N.oculatus* (like *N.abbreviatus*) is a very widespread Neotropical species, these reports should be treated as unconfirmed until molecular data from those localities is available.

#### ﻿ Novochares pallipes

Taxon classificationAnimaliaColeopteraHydrophilidae

(Brullé, 1841)

59C78BC2-26FB-5524-9D86-4CFFEF6979A5

Figs 9L–R , 10C

Hydrophilus (Philhydrus) pallipes Brullé, 1841: 58 - Uruguay, Montevideo.
Philhydrus pallipes (Brullé, 1841); Lacordaire 1854: 457.
Helochares pallipes (Brullé, 1841); Bedel 1881: XCIV.
Helochares (s. str.) pallipes (Brullé, 1841); Fernández 1983: 444; Fernández 1989: 148 [in key].
Novochares pallipes (Brullé, 1841); Girón and Short 2021: 205.

##### Material examined

**(14 exs.). Argentina: Buenos Aires**: Martinez, xii.1953 (2, CAS); San Isidro, xii.1955 (6, CAS, SEMC); Martinez, xii.1957 (2, USNM); Choya, “Sta. del Estero”, 10.iii.1962 (1, CAS). **Entre Ríos**: Río Paraná Ibicuy, Pto. Ibicuy, 10.xii.1979, leg. C.M. & S. Flint, Jr. (3, USNM).

##### Differential diagnosis.

The strongly curved parameres and the extremely large distinctive fork of the dorsal plate of the median lobe (Fig. 9L–R) serve to separate this species fairly easily from other members of the species group.

##### Description.

Body length 7.2–7.8 mm. ***Coloration***: Dorsal surfaces pale brown to orange-brown, sometimes with weakly paler (orange) clypeus and margins of pronotum and elytra. ***Head***: Maxillary palps nearly 1.3× width of head, uniformly yellow to orange in color. ***Thorax***: Elytra without rows of serial punctures, faint rows (one dorsal and two or three lateral) of scarce and weakly marked systematic punctures barely noticeable. Prosternum medially broadly convex. Posterior elevation of mesoventrite broadly elevated, with low medial longitudinal ridge extending anteriorly. ***Abdomen***: Apical emargination of fifth ventrite moderately deep and relatively narrow. ***Aedeagus***: (Fig. 9L–R) Apical region of each paramere medially and dorsally curved; outer margin of apical region of paramere rounded, latero-ventrally weakly pointed; dorsal plate of median lobe with long and relatively broad neck, widened at apical region; neck with paired longitudinal, laminar elevations along second 1/2; distal arms laminate, dorso-ventrally oriented, 1/2 as long as neck of dorsal plate of median lobe; ventral plate of median lobe narrow and triangular, moderately sclerotized, reaching to beyond base of fork of dorsal plate of median lobe.

##### Distribution.

Argentina, Uruguay (Fig. 10C). Prior records from Brazil and Paraguay were erroneously reported and are removed from the known distribution of this species.

##### Remarks.

There has been some confusion about the distribution of this species in the literature. We have only been able to confirm the presence of this species in Argentina and Uruguay. Literature reports of this species occurring in Mato Grosso do Sul (Corumbá) and Paraguay (Río Alto Paraná) appear to be derived from the original description of Helochares(s. str.)parhedrus. This species was briefly synonymized with *N.pallipes* but later discovered to actually be a synonym of *H.atratus* (Fernández, 1982). However, because for a short time the distributional records of *N.pallipes* and *H.parhedrus* were fused, this muddied the literature. Fernández (1983) appears to have accidentally repeated the fused distribution which then carried into later papers. The record of this species from the state of Minas Gerais, Brazil derives from d’Orchymont (1926). However, later d’Orchymont (1939) noted that these records were erroneous and referred them to *H.parhedrus*, which later was synonymized with *N.atratus*. We are not aware of any verified Brazilian records of *H.pallipes*.

#### ﻿ Novochares pilatus sp. nov.

Taxon classificationAnimaliaColeopteraHydrophilidae

CD969E23-9C1B-5673-9CBD-B3D53703FFC1

https://zoobank.org/7978A7D9-46F5-43ED-8BE0-BE0717644FCB

Figs 8P–R , 10C

##### Type material.

***Holotype* (male)**: “VENEZUELA: Bolivar State/ 6°35.617'N, 66°49.238'W, 80m/ Los Pijiguaos; outcrop/morichal/ 12.i.2009; leg. Miller & Short/ V09-0112-01C; detrital pools (MIZA). ***Paratypes*: (68 exs.): Venezuela: Barinas**: East of Santa Barbara, Rio Santa Barbara, 7°50.028'N, 71°11.188'W, 177 m, 25.i.2012, leg. Short, Arias, & Gustafson, big side pool of river, VZ12-0126-01B (1, SEMC, DNA Voucher SLE1241). **Bolívar**: same data as holotype (67, MIA, SEMC, TTU-Z, including DNA Voucher SLE1204).

##### Differential diagnosis.

This species is distinguished by a relatively extended and narrow expansion at the apex of the dorsal plate of the median lobe, which is also set with two modest arms (Fig. 8P, Q).

##### Description.

Body length 6.0–7.4 mm. ***Coloration***: Dorsal surfaces pale brown to yellowish brown, with paler (yellowish) clypeus and margins of pronotum and elytra. ***Head***: Maxillary palps 1.4–1.5× longer than width of head, uniformly yellow to orange in color. ***Thorax***: Elytra without rows of serial punctures, each with very faint rows (one dorsal and two or three lateral) of scarce and weakly marked systematic punctures. Prosternum only weakly convex. Posterior elevation of mesoventrite broadly and somewhat triangularly elevated, posteriorly weakly transversely impressed, with low medial longitudinal ridge extending anteriorly. ***Abdomen***: Apical emargination of fifth ventrite moderately deep and broad. ***Aedeagus***: (Fig. 8P–R) Outer margin and apical region of each paramere rounded at apex and laterally pointed; dorsal plate of median lobe with long and narrow neck, ovally expanded at apical region, with two narrow, elongated, laminate (dorsoventrally oriented), contiguous distal arms; arms as long as 1/2 length of base of fork; ventral plate of median lobe triangular, strongly sclerotized, apically pointed, reaching mid-length of dorsal plate.

##### Etymology.

*Pilatus* (L.) meaning large. In reference to the relatively large size of the aedeagus when compared with other species in the *abbreviatus* species group.

##### Distribution.

This species is found in several localities in central Venezuela (Fig. 10C).

##### Habitat.

The large main series of this species was collected in shallow detrital pools that were along the edge of a morichal that ran alongside a granite outcrop. Another specimen was collected in the side pools of a large river.

###### ﻿*Novocharesaperito* species group

**Figure 11.**
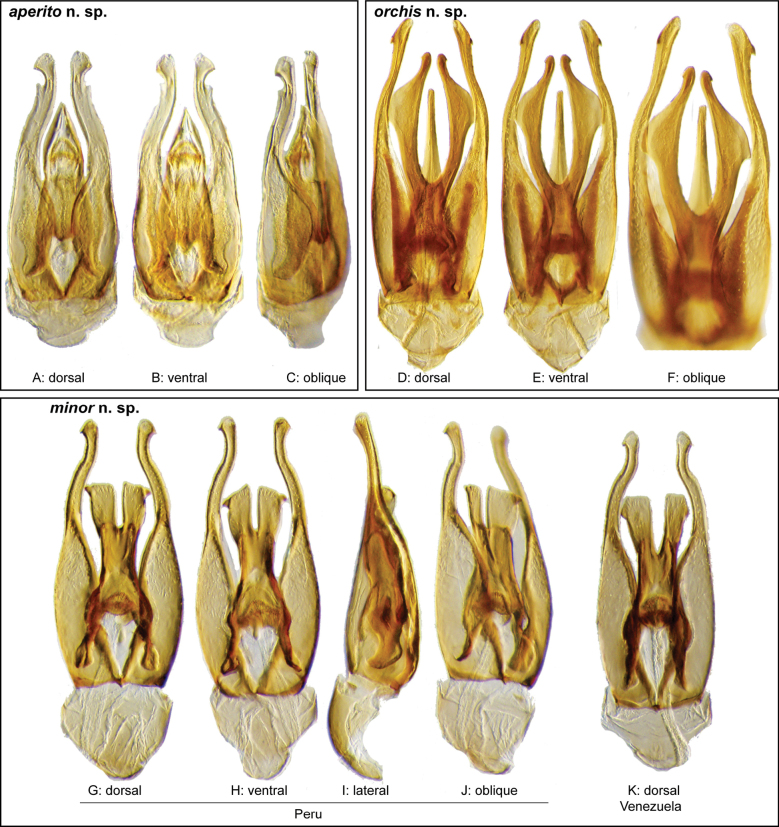
Aedeagi of *Novochares* spp. **A–C***N.aperito***D–F***N.orchis***G–K***N.minor***G–J** Peru **K** Venezuela **A, D, G, K** dorsal view **B, E, H** ventral view **C, F, J** oblique view **I** lateral view.

**Species group diagnosis.** Body length 4.9 mm. ***Coloration***: Dorsal surfaces orange, with paler clypeus and margins of pronotum. ***Head***: Maxillary palps slightly longer than width of head, uniformly orange in color. ***Thorax***: Ground punctation on pronotum and elytra relatively dense and very shallowly impressed. Elytra without rows of serial punctures, each with very faint rows of weakly marked systematic punctures on lateral 1/2. Prosternum weakly medially convex. Posterior elevation of mesoventrite transversely elevated. ***Abdomen***: Apical emargination of fifth ventrite relatively deep, U-shaped. ***Aedeagus***: (Fig. 11A–C) Overall shape pear-like, joint basal margins of parameres truncate; apical region of each paramere somewhat hook-like; parameres longer than median lobe, with apex rounded; dorsal inner margin of each paramere sinuate; dorsal plate of median lobe (in dorsal view) with neck weakly defined, with arms weakly defined; gonopore placed near apex of dorsal plate of median lobe; ventral plate of median lobe (in ventral view) membranous; basal piece nearly 0.3× length of a paramere. In lateral view, aedeagus somewhat triangular, straight at base, with ventral outline of parameres nearly 3× longer than greatest width near base.

**Composition.** This group is composed of a single known species from Bolivia (known from a single male) with a very unusual and distinctive genitalia: *N.aperito* sp. nov.

#### ﻿ Novochares aperito sp. nov.

Taxon classificationAnimaliaColeopteraHydrophilidae

8911FCCD-3262-5A14-9B31-EFFFE605F6A0

https://zoobank.org/0DE84FFA-2FB4-44C6-AC75-2B80606B148E

Figs 11A–C , 13A

##### Type material.

***Holotype* (male)**: “BOLIVIA: Santa Cruz Dept./ Potrerillos del Guenda,/ Preserva Natural, 17°40'S, 63°27'W, 370m, 12–13-X-2007/ ex. BL/MV, A.R Cline & J.E./ Wappes BOL1Cline07 004.5” (SEMC).

##### Differential diagnosis.

See species group diagnosis.

##### Description.

Body length 4.9 mm. ***Coloration***: Dorsal surfaces orange, with paler clypeus and margins of pronotum. ***Head***: Maxillary palps slightly longer than width of head, uniformly orange in color. ***Thorax***: Ground punctation on pronotum and elytra relatively dense and very shallowly impressed. Elytra without rows of serial punctures, each with very faint rows of weakly marked systematic punctures on lateral 1/2. Prosternum weakly medially convex. Posterior elevation of mesoventrite transversely elevated. ***Abdomen***: Apical emargination of fifth ventrite relatively deep, U-shaped. ***Aedeagus***: (Fig. 11A–C) 2.3× longer than wide, with outer lateral margins of parameres slightly convex along basal 1/2, then weakly sinuate up to apical region; apical region of each paramere rounded, with outer margin somewhat hook-like; at closest point, dorsal inner margins of parameres separated by distance 0.33× greatest width of a paramere; dorsal plate of median lobe with neck 0.7× as broad as base; arms of dorsal plate of median lobe not clearly indicated; apical region of dorsal plate of median lobe dorsally concave, apically pointed; ventral plate of median lobe membranous, neck of dorsal plate; basal piece 0.3× length of a paramere. In lateral view, aedeagus straight at base, with ventral outline of parameres 3× longer than greatest width near base; dorsal outline of aedeagus in lateral view slightly convex along basal 1/4, then oblique to apical region; ventral outline of aedeagus in lateral view straight.

##### Etymology.

*Aperito* (L.) meaning to open, referring to the distinctive shape of the apical region of the parameres which resemble a bottle opener.

##### Distribution.

Only known from the type locality in Bolivia (Fig. 13A).

##### Habitat.

Nothing is known about the habitat of this species as it was collected at lights.

##### Remarks.

Among the thousands of specimens studied here, we only found one specimen of this species, which might be an indicator of their rarity in nature, or a reflection of the lack of sampling in the lowlands of Bolivia. The specimen is in modest condition, with one maxillary palp and a few tarsi missing.

###### ﻿*Novocharesgarfo* species group

**Figure 12.**
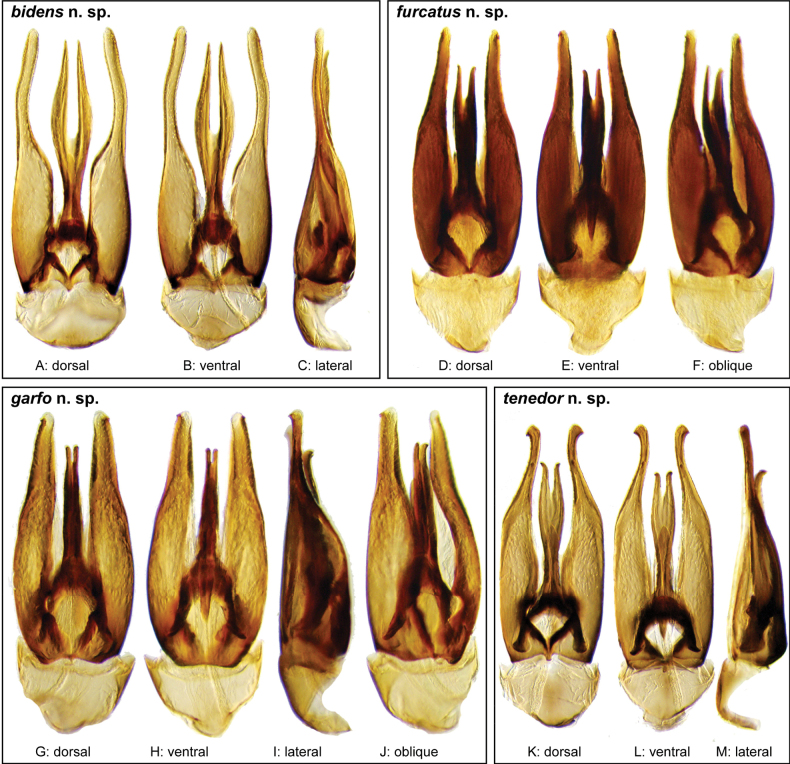
Aedeagi of the *Novocharesgarfo* species group **A–C***N.bidens***D–F***N.furcatus***G–J***N.garfo***K–M***N.tenedor***A, D, G, K** dorsal view **B, E, H, L** ventral view **C, I, M** lateral view **F, J** oblique view.

**Species group diagnosis.** Body length 4.9–6.5 mm. ***Coloration***: Dorsal surfaces pale brown (orange to yellowish), with slightly paler margins of pronotum and elytra, sometimes also clypeus. ***Head***: Maxillary palps slightly to 1.3× longer than width of head, uniformly orange or yellow in color. ***Thorax***: Ground punctation on pronotum and elytra relatively dense and shallowly impressed. Elytra without rows of serial punctures, each with very faint rows (sometimes one dorsal and two or three lateral, more usually only lateral) of scarce and weakly marked systematic punctures. Prosternum medially broadly and weakly convex. Posterior elevation of mesoventrite broadly elevated, somewhat transverse, often with glabrous longitudinal ridge extending anteriorly. ***Abdomen***: Apical emargination of fifth ventrite relatively deep, U- or V-shaped. ***Aedeagus***: (Fig. 12) Overall shape sub-rectangular or oval to pear-like, joint basal margins of parameres truncate to medially pointed or medially emarginate in dorsal view; outer margin and apical region of each paramere weakly to strongly pointed; parameres longer than median lobe, with apex rounded; parameres with apical region variable in degree of sclerotization; dorsal inner margin of each paramere straight to sinuate; dorsal plate of median lobe (in dorsal view) forming a narrow neck; notch between arms variable; arms usually parallel, variable in shape and length; gonopore placed at or near base of dorsal plate of median lobe; ventral plate of median lobe moderately sclerotized, triangular, slender, and apically rounded, not reaching base of fork of dorsal plate; basal piece nearly 0.3× length of a paramere. In lateral view, aedeagus somewhat triangular, straight to weakly oblique at base, with ventral outline of parameres 3–4× longer than greatest width.

**Composition.** The *Novocharesgarfo* species group is composed of four species: *Novocharesbidens* sp. nov., *N.furcatus* sp. nov., *N.garfo* sp. nov., and *N.tenedor* sp. nov.

**Remarks.** All four species in this group are relatively light brown/yellow in dorsal coloration, making them resemble the much more common members of the *abbreviatus* species group. Species in this group can be recognized by the relatively simple shape of the dorsal plate of the median lobe which resembles a bifid fork in all four species, as well as the shape of the parameres, which are typically straighter and with only a very small tooth near the apex (except *N.tenedor*, in which the apex is more curved and well developed).

#### ﻿ Novochares bidens sp. nov.

Taxon classificationAnimaliaColeopteraHydrophilidae

70F87D86-9684-5A2B-84C6-89F733660A91

https://zoobank.org/C6A7B51F-54C9-4F36-A157-D10A6252A456

Figs 12A–C , 13B

##### Type material.

***Holotype* (male)**: “BRAZIL, M.G./ Jacare, P.N. Zingu/ XI-1965, at lite/ M. Alvarenga” (USNM). ***Paratypes* (2 exs.): Brazil: Mato Grosso**: Tapirape Indian Village at confluence of R. Tapirape and R. Araguaia, 26–31.xii.1960, leg. B. Malkin, at light (1, FMNH). **Mato Grosso**: Same data as holotype (1, SEMC).

##### Differential diagnosis.

This species is easily distinguished from others in the species group by the extremely large and deeply cleft dorsal plate of the median lobe, and the unusually narrow and apically rounded parameres (Fig. 12A, B).

##### Description.

Body length 5.9–6.4 mm. ***Coloration***: Dorsal surfaces pale (yellowish) brown, with slightly paler (orange) margins of pronotum and elytra. ***Head***: Maxillary palps nearly 1.3× longer than width of head, uniformly orange in color. ***Thorax***: Elytra without rows of serial punctures, each with very faint rows (one dorsal and two or three lateral) of scarce and weakly marked systematic punctures. Prosternum medially weakly convex. Posterior elevation of mesoventrite broadly elevated, posteriorly somewhat transverse, with low and glabrous longitudinal ridge extending anteriorly. ***Abdomen***: Apical emargination of fifth ventrite relatively deep, U-shaped. ***Aedeagus***: (Fig. 12A–C) Overall shape sub-rectangular, 2.2× longer than wide, with outer lateral margins of parameres weakly sinuate; joint basal margins of parameres medially pointed in dorsal view; apical region of each paramere rounded, partly membranous, with outer margin weakly pointed; at closest point (near base of neck), dorsal inner margins of parameres separated by nearly 0.8× greatest width of a paramere; dorsal plate of median lobe with neck 0.28× as broad as base; arms of dorsal plate of median lobe dorsally concave, with inner margins nearly parallel, gradually narrowing towards apex, nearly 0.4× length of dorsal plate of median lobe; each arm acute at apex; notch between arms at base nearly as wide as base of an arm; basal piece nearly 0.3× length of a paramere. In lateral view, aedeagus straight at base, with ventral outline of parameres 4.2× longer than greatest width near base; dorsal outline of aedeagus in lateral view very weakly convex along basal 1/2, then concave along distal 1/2; ventral outline of aedeagus in lateral view sinuate.

##### Etymology.

*Bidens* (L.), meaning two-pronged fork, referring to the shape of the dorsal plate of the median lobe of this species.

##### Distribution.

**Figure 13.**
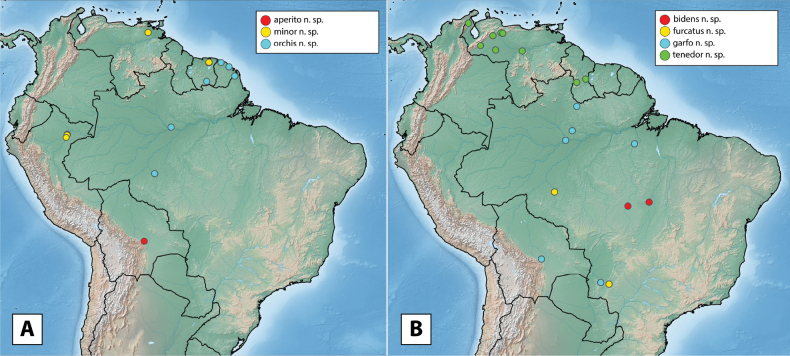
Distribution of *Novochares* spp. **A***N.aperito* (red), *N.minor* (yellow), *N.orchis* (blue) **B***N.bidens* (red), *N.furcatus* (yellow), *N.garfo* (blue), *N.tenedor* (green).

Only known from the type locality in central Brazil (Fig. 13B).

##### Habitat.

Nothing is known about the habitat of this species.

#### ﻿ Novochares furcatus sp. nov.

Taxon classificationAnimaliaColeopteraHydrophilidae

599688BE-EB5F-5A4A-B8DF-3FC2A49A53A9

https://zoobank.org/DC5948F8-88E5-45AA-9921-0EDB6A1877FB

Figs 12D–F , 13B

##### Type material.

***Holotype* (male)**: “BRAZIL: Mato Grosso do Sul/ -20.51369°, -55.42803°, 240 m/ Palmeiras (c. 7 km S) on MS-450/ 22.vi.2018; leg. Hamada & team/ Pond in field w/dense vegetation/ BR18-0622-01A” (INPA). ***Paratype* (1 ex.): Brazil: Rondônia**: Machadinho d’Oeste, Balneario São Jose, -9.44573, -61.98332, 103 m, 9.vii.2018, leg. Short, margins of various places along river, BR18-0709-01A (1, SEMC, DNA voucher SLE2097).

##### Differential diagnosis.

Among members of this species group, this species is most similar to *N.garfo*: both species share a relatively straight and parallel-sided dorsal plate of the median lobe with two relatively short arms at the apex (Fig. 12D, H). These arms are distinctly longer in *N.furcatus* (Fig. 12D), while they are barely noticeable in *N.garfo* (Fig. 12G). Additionally, the parameres are slightly narrower along the apical 1/3 in *N.furcatus* than in *N.garfo*.

##### Description.

Body length 5.6 mm. ***Coloration***: Dorsal surfaces pale (yellowish) brown, with slightly paler (yellow) margins of pronotum and elytra. ***Head***: Maxillary palps slightly longer than width of head, uniformly yellow in color. ***Thorax***: Elytra without rows of serial punctures, each with very faint rows of scarce and weakly marked systematic punctures on lateral region. Prosternum broadly and weakly convex. Posterior elevation of mesoventrite somewhat transverse and broadly elevated. ***Abdomen***: Apical emargination of fifth ventrite relatively deep, U-shaped. ***Aedeagus***: (Fig. 12D–F) Overall shape pear-like, 2.2× longer than wide, with outer lateral margins of parameres evenly convex up to apical region; joint basal margins of parameres medially emarginate in dorsal view; apical region of each paramere rounded to truncate, partly membranous, with outer margin weakly pointed; at closest point (near base of neck), dorsal inner margins of parameres separated by distance slightly narrower than greatest width of a paramere; dorsal plate of median lobe with neck 0.3× as broad as base; base of arms of dorsal plate of median lobe dorsally concave; arms of dorsal plate nearly parallel, gradually narrowing towards apex, nearly 0.16× length of dorsal plate of median lobe; each arm acute and dorsally pointed at apex; notch between arms at base slightly narrower than base of an arm; basal piece nearly 0.32× length of a paramere. In lateral view, aedeagus weakly oblique at base, with ventral outline of parameres 4× longer than greatest width near base; dorsal outline of aedeagus in lateral view evenly convex along basal 2/3, then nearly straight along distal 1/3; ventral outline of aedeagus in lateral view nearly straight.

##### Etymology.

*Furcatus* (L.), meaning split in two, referring to the shape of the dorsal plate of the median lobe of this species.

##### Distribution.

Known from two localities in the Brazilian states of Mato Grosso do Sul and Rondônia (Fig. 13B).

##### Habitat.

One specimen was taken in the margins of an open pond, the other was taken along the margins of a river.

#### ﻿ Novochares garfo sp. nov.

Taxon classificationAnimaliaColeopteraHydrophilidae

74F3C31A-FCF3-5898-BE21-5FB7AD85170A

https://zoobank.org/9E9B36D7-EE62-4DFD-AF0A-7DCC0745FF7C

Figs 12G–J , 13B

##### Type material.

***Holotype* (male)**: “BRAZIL: Roraima: Caroebe/ 00°50.939'N, 59°18.262'W; 145m/ Rio Jatapu, nr. Usina de Jatapu;/ marginal pools of river; 17.i.2018/ leg. A. Short; BR18-0117-02A (INPA). ***Paratypes* (11 exs.): Brazil: Amazonas**: Manacapuru, -3.23037, -60.64269, 35 m, 9.vi.3017, leg. Benetti, margin of large marsh, BR17-0609-01A (1, SEMC, DNA voucher SLE1263); Presidente Figueiredo, (ca. 19 km E) on AM-240, Igarape Pantera, -2.04243, -59.84914, 17.i.2018, leg. Short, margin of small side stream, vegetation, and detritus, BR18-0617-01A (1, SEMC, DNA voucher SLE1931). **Mato Grosso do Sul**: Miranda (ca. 9.5 km SW) on MS-339, -20.32119, -56.42563, 131 m, 26.vi.2018, leg. Hamada & team, marshy area alongside stream, BR18-0626-03A (3, SEMC, including DNA voucher SLE2103). **Pará**: Rio Xingu Camp, ca. 60 km S. Altamira, Igarape Jabuti, 8–16.x.1986, leg. P. Spangler & O. Flint, malaise trap (2, USNM). **Roraima**: Same data as holotype (3, INPA, SEMC). **Bolivia: Santa Cruz**: 3.7 km SSE Buena Vista, Hotel Flora y Fauna, 1–12.v.2004, leg. A.R. Cline, MV+HG lights (1, SEMC).

##### Differential diagnosis.

See differential diagnosis of *N.furcatus*.

##### Description.

Body length 4.9–5.8 mm. ***Coloration***: Dorsal surfaces pale (yellowish) brown, usually with slightly paler (yellow) clypeus and margins of pronotum and elytra. ***Head***: Maxillary palps slightly longer than width of head, uniformly yellow in color. ***Thorax***: Elytra without rows of serial punctures, each with very faint rows of scarce and weakly marked systematic punctures on lateral and posterior regions. Prosternum medially broadly and weakly convex. Posterior elevation of mesoventrite somewhat transverse and broadly elevated, with low and glabrous longitudinal ridge extending anteriorly. ***Abdomen***: Apical emargination of fifth ventrite relatively deep, U- or V-shaped. ***Aedeagus***: (Fig. 12G–J) Overall shape pear-like, 2.1× longer than wide, with outer lateral margins of parameres evenly convex up to apical region; joint basal margins of parameres medially emarginate in dorsal view; apical region of each paramere rounded, partly membranous, with outer margin ventro-laterally pointed; at closest point (near base of neck), dorsal inner margins of parameres separated by distance 0.8× greatest width of a paramere; dorsal plate of median lobe with neck 0.38× as broad as base; base of arms of dorsal plate of median lobe dorsally concave; arms of dorsal plate parallel, parallel-sided along entire length, nearly 0.1× length of dorsal plate of median lobe; each arm acute and dorsally pointed at apex; notch between arms at base slightly narrower than base of an arm; basal piece nearly 0.31× length of a paramere. In lateral view, aedeagus weakly oblique at base, with ventral outline of parameres 3.3× longer than greatest width near base; dorsal outline of aedeagus in lateral view very weakly convex along basal 1/2, then concave along distal 1/2; ventral outline of aedeagus in lateral view nearly straight.

##### Etymology.

Garfo, meaning fork in Portuguese, in reference to the shape of the dorsal plate of the median lobe.

##### Distribution.

Brazil (Amazonas, Mato Grosso do Sul, Pará, Roraima), Bolivia (Fig. 13B).

##### Habitat.

This species has been collected in open marshes as well as along the margins of open rivers.

#### ﻿ Novochares tenedor sp. nov.

Taxon classificationAnimaliaColeopteraHydrophilidae

F3445DA1-0264-5F5A-A4C9-FE7CB3229119

https://zoobank.org/B95F2EE8-D28B-4EAE-B161-AA2783A25F57

Figs 12K–M , 13B

##### Type material.

***Holotype* (male)**: “VENEZUELA: Apure State/ 7°37.289'N, 69°3.679'W, 83m/ side road ca. 10 km E. Mantecal/ leg. Short, García, & Camacho/ 18.i.2009; marshy area and pool by road; VZ09-0118-02X” (MIZA). ***Paratypes* (59 exs.): Guyana: Region 6**: Upper Berbice, Basecamp 1, 4°08.809'N, 58°14.232'W, 108 m, 22.ix.2014, leg. Short, Salisbury, La Cruz, margin of Berbice River, GY14-0922-02A (36, SEMC, CBDG, TTU-Z, including DNA voucher SLE1219). **Region 9**: Karanambu, 3°45.1'N, 59°18.6'W, Rupununi River, 2.iv.1994, leg. P.J. Spangler, colln #8 (1, USNM). **Venezuela: Apure**: Same data as holotype (1, SEMC, DNA voucher SLE1205). **Barinas**: SW of Batatuy, 8°10.259'N, 70°51.866'W, 275 m, 25.i.2012, leg. Short, Arias, & Gustafson, sandbar/gravel margin, VZ12-0125-03C (1, SEMC). **Bolívar**: Cuchivero, 30 km SE of Caicara, 4.viii.1987, leg. S. & J. Peck, Woodland, UV light, SBP87-108 (6, SEMC). **Cojedes**: Rio Tinaco, near, approx. 5 km NE of Tinaco, 9°44.160'N, 68°24.219'W, 170 m, 20.i.2012, leg. Short, Arias, & Gustafson, stream margins, VZ12-0120-02A (4, SEMC); El Pao, approx. 7.5 km, Rio Caiman Grande at San Brano, 9°39.246'N, 68°11.860'W, 137 m, 20.i.2012, leg. Short, Arias, & Gustafson, stream margins, VZ12-0120-03A (3, SEMC); Aparicion, at highway, lagoon/pond, 9°22.268'N, 69°23.062'W, 213 m, 22.i.2012, leg. Short, Arias, & Gustafson, pond, VZ12-0122-01A (2, SEMC). **Portuguesa**: Aparicion, Rio Are, 9°22.900'N, 69°23.153'W, 220 m, 22.i.2012, leg. Short & Arias, river margin, VZ12-0122-02A (5, SEMC). **Zulia**: Quebrada Riencito, 10.86041°N, 72.32210°W, 95 m, 30.xii.2008, leg. Short & García, along margin, VZ08-1230-01B (1, SEMC).

##### Differential diagnosis.

This taxon is unique among members of this species group in having the parameres strongly sinuate along the apical 1/3 (Fig. 12K, L). While the dorsal plate of the median lobe is slightly similar in form to *N.bidens* (Fig. 12A, B), the arms of the fork are less than 1/2 as long, and the plate itself is much shorter, sitting well below the apex of the parameres.

##### Description.

Body length 5.1–6.5 mm. ***Coloration***: Dorsal surfaces orange to yellowish brown, usually with slightly paler (yellow) clypeus and margins of pronotum and elytra. ***Head***: Maxillary palps slightly nearly 1.3× longer than width of head, uniformly orange in color. ***Thorax***: Elytra without rows of serial punctures, each with very faint rows of scarce and weakly marked systematic punctures on lateral and posterior regions. Prosternum medially broadly and weakly convex. Posterior elevation of mesoventrite somewhat transverse and broadly elevated, with low and glabrous longitudinal ridge extending anteriorly. ***Abdomen***: Apical emargination of fifth ventrite relatively deep, U-shaped. ***Aedeagus***: (Fig. 12K–M) Overall shape pear-like, 2.3× longer than wide, with outer lateral margins of parameres very weakly convex up to apical region; joint basal margins of parameres medially pointed in dorsal view; apical region of each paramere rounded, with outer margin laterally pointed; at closest point (along neck), dorsal inner margins of parameres separated by distance 0.54× greatest width of a paramere; dorsal plate of median lobe with neck 0.23× as broad as base; base of arms of dorsal plate of median lobe dorsally concave; arms of dorsal plate somewhat parallel, diverging at apex, nearly parallel-sided for most length, nearly 0.16× length of dorsal plate of median lobe; each arm acute and dorso-laterally pointed at apex; notch between arms at base nearly 1/2 as wide as base of an arm; basal piece nearly 0.3× length of a paramere. In lateral view, aedeagus straight at base, with ventral outline of parameres 3.9× longer than greatest width near base; dorsal outline of aedeagus in lateral view strongly convex at base, then nearly straight and oblique along basal 1/2, then sinuate along distal 1/2; ventral outline of aedeagus in lateral view nearly straight.

##### Etymology.

Tenedor, meaning fork in Spanish, in reference to the shape of the dorsal plate of the median lobe.

##### Distribution.

Guyana, Venezuela (Fig. 13B).

##### Habitat.

This species has been collected in a range of habitats, though most specimens seem to have been taken in riparian areas along the margins of streams and rivers. Other specimens have been collected in more typical marsh habitats.

###### ﻿*Novocharesminor* species group

**Species group diagnosis.** Body length 4.2–5.4 mm. ***Coloration***: Dorsal surfaces very dark brown, with paler (orange to yellow) margins of pronotum and elytra. ***Head***: Maxillary palps slightly longer than width of head, orange to brown in color, usually paler (yellow) at ends of each palpomere (Fig. 7C). ***Thorax***: Ground punctation on pronotum and elytra relatively dense and shallowly impressed. Elytra without rows of serial punctures, each with very faint rows (one dorsal and two or three lateral) of scarce and weakly marked systematic punctures. Prosternum only very weakly medially convex. Posterior elevation of mesoventrite broadly and somewhat transversely elevated; mesoventrite with medial longitudinal glabrous patch extending anteriorly. ***Abdomen***: Apical emargination of fifth ventrite relatively deep, U-shaped. ***Aedeagus***: (Fig. 11G–K) Overall shape pear-like, joint basal margins of parameres somewhat truncate; apical region of each paramere sinuate and laterally pointed; parameres longer than median lobe, with apex rounded; dorsal inner margin of each paramere sinuate; dorsal plate of median lobe (in dorsal view) with broad neck and broad arms; gonopore placed at base of dorsal plate of median lobe; ventral plate of median lobe (in ventral view) membranous and short; basal piece nearly 0.3× length of a paramere. In lateral view, aedeagus somewhat triangular, oblique at base, with ventral outline of parameres nearly 3.5× longer than greatest width near base.

**Composition.** This group is composed of a single known species from Peru and Venezuela: *N.minor* sp. nov.

**Remarks.** The very small size combined with very dark to nearly black dorsal coloration serves to separate this species from most others. Most very dark species are typically much larger.

#### ﻿ Novochares minor sp. nov.

Taxon classificationAnimaliaColeopteraHydrophilidae

21D83E7F-2AD0-5DB6-917E-587C2A0678CD

https://zoobank.org/26D71EB9-D445-449F-BDCB-DE9CBD643DDA

Figs 7C , 11G–K , 13A

##### Type material.

***Holotype* (male)**: “PERU: Loreto: Maynas Province/ 3°50.723'S, 73°22.187'W, 113m/ ca. 10km SW Iquitos, nr. Facultad/ de Ciencias Biologicas UNAP/ leg. S. Baca, 18.i.2020/ seasonal pond; PE20-0118-03A” (MHNSM). ***Paratypes* (41 exs.): Peru: Loreto**: Same data as holotype (27, NZCS, SEMC, TTU-Z, including DNA voucher SLE2143); ca. 15 km SW Iquitos, on Iquitos-Nauta Highway, leg. S. Baca, 18.i.2020, flooded area with vegetation and detritus, PE20-0118-05A (10, SEMC); ca. 20 km SW Iquitos, on Iquitos-Nauta Hwy, 3°56.655'S, 73°23.853'W, 107 m, leg. S. Baca, 20.i.2020, shallow margins of lake, with vegetation/detritus; PE20-0120-01A (1, SEMC); ca. 60 km SW Iquitos, on Iquitos-Nauta Hwy, 4°16.279'S, 73°30.734'W, 95 m, leg. S. Baca, 20.i.2020, margin of small creek, inundated grass, PE20-0120-02A (1, SEMC). **Suriname: Para**: along Martin Luther King Hwy, blackwater marsh by road, 5.4204, -55.09876, SR12-0723-04A (1, SEMC, DNA voucher SLE535). **Venezuela: Monagas State**: S of Maturin, morichal at road crossing, 9°16.398'N, 62°56.246'W, 22 m, 2.ii.2010, leg. Short, García, & Joly, morichal margin, VZ10-0202-02A (1, MIZA).

##### Differential diagnosis.

See species group diagnosis.

##### Description.

Body length 4.2–5.4 mm. ***Coloration***: Dorsal surfaces very dark brown, with paler (orange to yellow) margins of pronotum and elytra. ***Head***: Maxillary palps slightly longer than width of head, orange to brown in color, usually paler (yellow) at ends of each palpomere (Fig. 7C). ***Thorax***: Ground punctation on pronotum and elytra relatively dense and shallowly impressed. Elytra without rows of serial punctures, each with very faint rows (one dorsal and two or three lateral) of scarce and weakly marked systematic punctures. Prosternum only very weakly medially convex. Posterior elevation of mesoventrite broadly and somewhat transversely elevated; mesoventrite with medial longitudinal glabrous patch extending anteriorly. ***Abdomen***: Apical emargination of fifth ventrite relatively deep, U-shaped. ***Aedeagus***: (Fig. 11G–K) Overall shape pear-like, 2.1–2.3× longer than wide, with outer lateral margins of parameres convex up to apical region; apical region of each paramere rounded; at closest point (along neck), dorsal inner margins of parameres separated by distance slightly narrower than greatest width of a paramere; dorsal plate of median lobe with neck 0.5× as broad as base; base of arms of dorsal plate of median lobe dorsally concave; arms of dorsal plate somewhat parallel to slightly diverging, nearly parallel-sided for most length, nearly 0.23× length of dorsal plate of median lobe; each arm truncate at apex, sometimes obliquely so, inner margin extending beyond outer margin; notch between arms at base nearly 0.4× base of an arm; basal piece nearly 0.3× length of a paramere. In lateral view, ventral outline of parameres 3.5× longer than greatest width near base; dorsal outline of aedeagus in lateral view strongly convex at base, then very weakly convex along basal 1/2, then sinuate along distal 1/2; ventral outline of aedeagus in lateral view sinuate.

##### Etymology.

Minor, named after its small body size.

##### Distribution.

Peru, Suriname, Venezuela (Fig. 13A).

##### Habitat.

This species has been found primarily in open marshes and along the margins of vegetated creeks.

##### Remarks.

There are small differences in overall length/width of aedeagus and the shape of the apicolateral region of the arms of the dorsal plate of the median lobe between specimens from Peru and Venezuela. The small size and form of this species allow it to be easily confused with *Sindolus* Sharp, but that genus can easily be separated by the strongly raised longitudinal carina of the mesoventrite, which is absent in *Novochares*.

###### ﻿*Novocharesorchis* species group

**Species group diagnosis.** Body length 7.3–9.3 mm. ***Coloration***: Dorsal surfaces dark brown and sheeny, with paler (brown or reddish brown) clypeus and margins of pronotum and elytra. ***Head***: Maxillary palps nearly 1.3× longer than width of head, uniformly reddish brown in color (Fig. 24B). ***Thorax***: Ground punctation on pronotum and elytra relatively dense and very shallowly impressed. Elytra without rows of serial punctures, each with very faint rows (one dorsal and two or three lateral) of scarce and weakly marked systematic punctures. Prosternum only very weakly medially convex. Posterior elevation of mesoventrite elevated as a triangular pyramid, with medial longitudinal ridge extending anteriorly. ***Abdomen***: Apical emargination of fifth ventrite relatively deep, V- or U-shaped. ***Aedeagus***: (Figs 4B, 11D–F) Overall shape sub-rectangular, nearly 3× longer than wide, joint basal margins of parameres truncate; outer margin of each paramere nearly straight along basal 1/2, then weakly sinuate; apical region of outer margin of each paramere pointed; parameres longer than median lobe; apex of parameres rounded to truncate; parameres with apical region variable in degree of sclerotization; dorsal inner margin of each paramere strongly sinuate; dorsal plate of median lobe (in dorsal view) with stout and strongly sclerotized basal apodemes; dorsal plate of median lobe narrower along mid-section than at base, forming a short and broad neck; notch between arms broad; gonopore sitting proximal to base of median lobe; ventral plate of median lobe (in ventral view) triangular, moderately sclerotized; basal piece nearly 0.35× length of a paramere, with distal margin medially emarginate. In lateral view, aedeagus triangular, weakly oblique at base, with ventral outline of parameres about 4× longer than greatest width near base.

**Composition.** This group is composed of a single known species that is found throughout much of the Guiana Shield region with an extremely elaborate aedeagus: *N.orchis* sp. nov.

**Remarks.** The large size, very dark brown coloration, and strongly elevated mesoventrite serve to separate this species group from all except the *tectiformis* species group. However, we are not aware of any external morphological characters to distinguish it from that group. The species has been placed in its own group largely based on the molecular phylogenetic placement (Fig. 1).

#### ﻿ Novochares orchis sp. nov.

Taxon classificationAnimaliaColeopteraHydrophilidae

2D4C7E33-5799-5C74-BB4B-F0C0CA82C17F

https://zoobank.org/EC044F4E-2E7D-460A-8696-3DAC9AA14E34

Figs 4B , 11D–F , 13A , 24B

##### Type material.

***Holotype* (male)**: “SURINAM/ Suriname Dist./ Krakka-Phedra Rd./ X-25-1962/ Borys Malkin”, “tiny pool in/forest, much/ fallen foliage” (USNM). ***Paratypes* (57 exs.): Brazil: Amapá**: Oiapoque (ca. 22 km S) on BR -156, 3.65822, -51.76958, leg. Short, forested detrital pools, BR18-0720-01B (1, SEMC, including DNA voucher SLE1851). **Amazonas**: Manaus Ducke Reserve, Igarape Barro Branco, -2.93079, -59.97514, 75 m, 6.vi.2018, leg. Short & team, stream margins, BR18-0606-02B (11, INPA, SEMC, TTU-Z); same data except forest pools/riparian area by stream, BR18-0606-02C (1, SEMC); same data except shallow pools, BR18-0606-02D (1, SEMC); same data except 9.vi.2018, muddy pools in swampy area by stream, BR18-0609-02B (1, SEMC). **Rondônia**: Tabajara (ca. 4.5 km W) on RO-133, -8.9217, -62.0978, 100 m, 8vii.2018, leg. Short, detrital pool/marsh by stream, BR18-0708-02B (2, SEMC). **French Guiana**: Piste de montagne de fer, 5.37641, -53.54782, 67 m, 3.iii.2020, leg. Short & Neff, large shallow detrital pool by road, FG20-0303-02A (4, SEMC); Forêt des Sables Blancs Park, 3.iii.2020, leg. Short & Neff, detrital puddle in forest, FG20-0303-03A (2, SEMC); Bagne des Annamites Park, Crique Anguille, 4.83287°N, -52.5145°W, 17 m, leg. Short & Neff, small sandy stream with detritus, FG20-0307-01B (5, SCC, SEMC). **Suriname: Suriname**: same data as holotype (25, SEMC, UNSM). **Sipaliwini District**: Camp 4 (low), Kasikasima; sandy/ creek, trail to Kasikasima, 2.97731°N, 55.38500°W, 200 m, 22.iii.2012, leg. A. Short, SR12-0322-02A (4, NSCS, SEMC, including SLE1214).

##### Differential diagnosis.

See species group diagnosis.

##### Description.

Body length 7.3–9.3 mm. ***Coloration***: Dorsal surfaces dark brown and sheeny, with paler (brown or reddish brown) clypeus and margins of pronotum and elytra. ***Head***: Maxillary palps nearly 1.3× longer than width of head, uniformly reddish brown in color (Fig. 24B). ***Thorax***: Ground punctation on pronotum and elytra relatively dense and very shallowly impressed. Elytra without rows of serial punctures, each with very faint rows (one dorsal and two or three lateral) of scarce and weakly marked systematic punctures. Prosternum only very weakly medially convex. Posterior elevation of mesoventrite elevated as a triangular pyramid, with medial longitudinal ridge extending anteriorly. ***Abdomen***: Apical emargination of fifth ventrite relatively deep, V- or U-shaped. ***Aedeagus***: (Figs 4B 11D, F) apical region of outer margin of each paramere pointed, dorsally directed; parameres longer than median lobe; at closest point, dorsal inner margins of parameres separated by distance 0.5× greatest width of a paramere; dorsal plate of median lobe with neck 0.5× as broad as base; arms of dorsal plate of median lobe abruptly broadening at mid-length, slightly converging at apex, with apex rounded; apical region of outer margin of each arm pointed; arms nearly 0.55× length of dorsal plate of median lobe; notch between arms at base nearly as broad as base of an arm; ventral plate of median lobe apically truncate, apex extending to apical third 1/4 of dorsal plate; basal piece 0.35× length of a paramere. In lateral view, aedeagus somewhat teardrop-shaped, with ventral outline of parameres 4× longer than greatest width near base; dorsal outline nearly evenly convex along entire length.

##### Etymology.

This species is named after the complex and intricate shape of the aedeagus, which we had informally named the “orchid one” during the course of this revision.

##### Distribution.

Known from Brazil (Amapá, Amazonas, Rondônia), French Guiana, and Suriname (Fig. 13A).

##### Habitat.

This species is most commonly found along detrital margins of streams and in forested pools associated with streams.

###### ﻿*Novocharespunctatostriatus* species group

**Figure 14.**
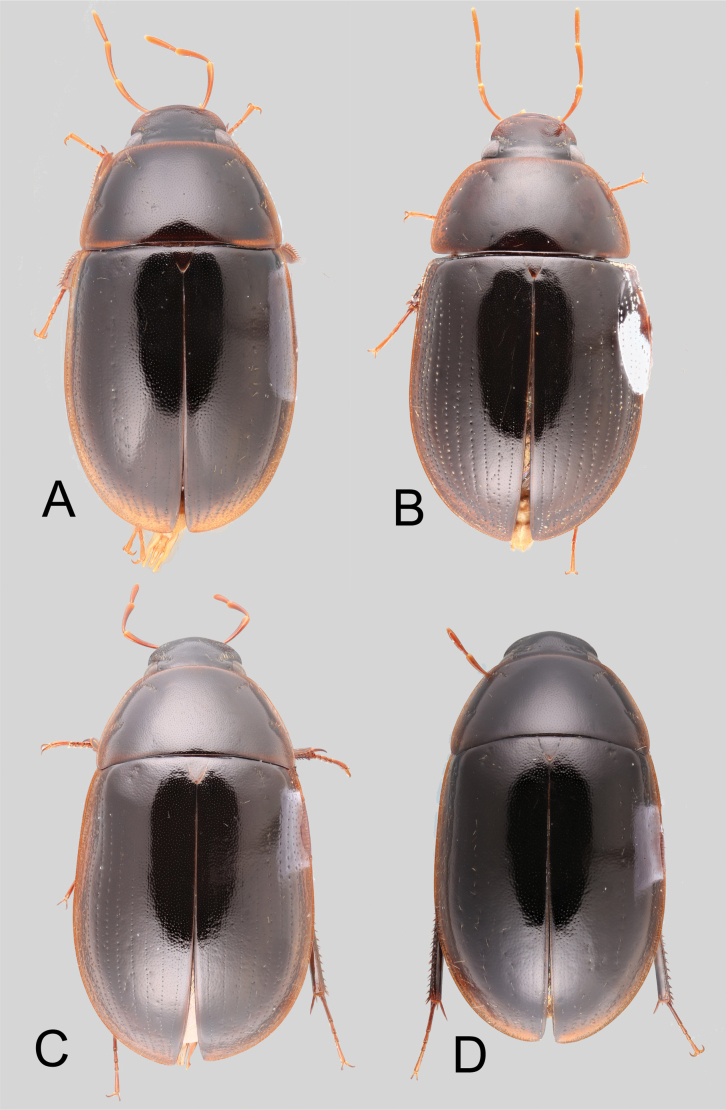
Dorsal habitus of *Novochares* spp. **A***N.dentatus***B***N.punctatostriatus***C***N.pertusus***D***N.triangularis*.

**Figure 15.**
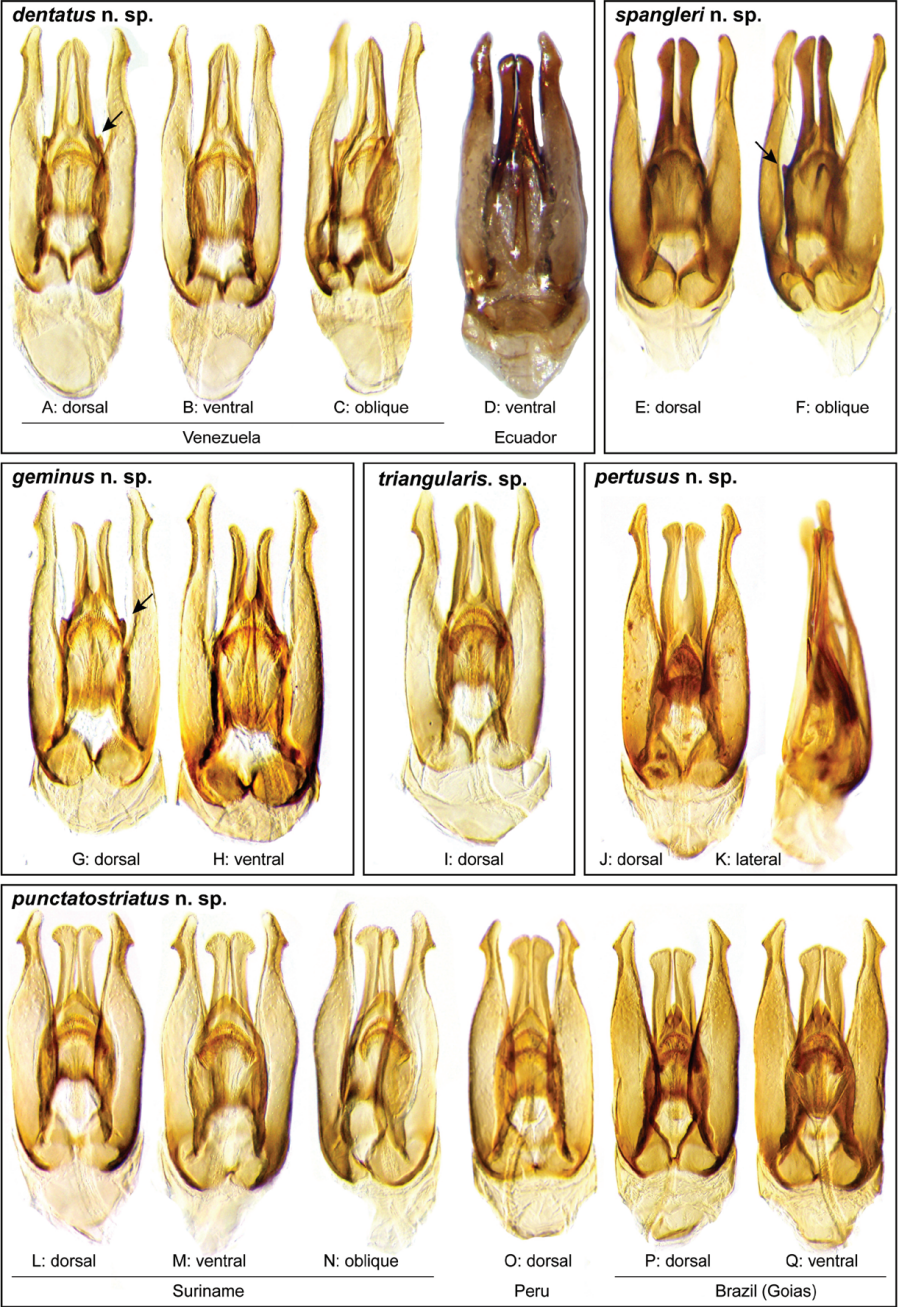
Aedeagi of the *Novocharespunctatostriatus* species group **A–D***N.dentatus***E, F***N.spangleri***G, H***N.geminus***I***N.triangularis***J, K***N.pertusus***L–Q***N.punctatostriatus***A–C** Venezuela **D** Ecuador **L–N** Suriname **O** Peru **P, Q** Brazil. **A, E, G, I, J, L, O, P** dorsal view **H, M, Q** ventral view **C, F, N** oblique view **K** lateral view.

**Species group description.** Body length 4.7–8.8 mm. ***Coloration***: Dorsal surfaces dark brown, usually with paler (orange to yellow) margins of head, pronotum, and elytra. ***Head***: Maxillary palps slightly shorter to slightly longer than width of head, uniformly yellow to orange in color (Fig. 14). ***Thorax***: Ground punctation on pronotum and elytra relatively dense and shallowly impressed. Elytra sometimes with defined rows of serial punctures, at least along posterior and lateral areas, not forming grooves. Prosternum flat to medially convex. Posterior elevation of mesoventrite transverse, usually blunt and low. ***Abdomen***: Apical emargination of fifth ventrite small and shallow, slightly broader than deep. ***Aedeagus***: (Figs 4A, 15) Overall shape sub-rectangular, approximately 2× as long as wide, joint basal margins of parameres medially emarginate and laterally rounded; outer margin of each paramere nearly straight or very weakly convex along basal 2/3, then concave and laterally pointed, then oblique to apex; parameres longer than median lobe; apex rounded; parameres with apical region cylindrical (hollow to apex) and lightly sclerotized; dorsal inner margin of each paramere sinuate; dorsal plate of median lobe (in dorsal view) with strongly sclerotized basal apodemes (nearly 0.25× length of plate); dorsal plate of median lobe nearly parallel-sided along basal 2/3, then bifurcating with parallel to distally converging arms; notch between arms variable; shape and orientation of arms variable; gonopore sitting proximal to base of fork of dorsal plate; ventral plate of median lobe (in ventral view) somewhat triangular, variable in length, shape of apex, and degree of sclerotization; dorsal surface of ventral plate of median lobe slightly concave (sides curved dorsally); basal piece nearly 0.25–0.40× length of a paramere, with distal margin straight. In lateral view, aedeagus triangular, strongly oblique at base, with ventral outline of parameres 3–5× longer than greatest width near base.

**Composition.** The *Novocharespunctatostriatus* species group is composed of six species: *Novocharesdentatus* sp. nov., *N.geminus* sp. nov., *N.pertusus* sp. nov., *N.punctatostriatus* sp. nov., *N.spangleri* sp. nov., and *N.triangularis* sp. nov.

**Remarks.** This species group is united by several distinct morphological characters and was recovered as a monophyletic group by Short et al. (2021). However, our two-gene analysis did not recover the monophyly of this species group, instead recovering two distinct, sequentially diverging lineages (Fig. 1). These two subgroups can be morphologically differentiated: the lineage that contains *N.dentatus*, *N.geminus*, and *N.spangleri* have lateral teeth on the dorsal plate of the median lobe of the aedeagus combined with an extended ventral plate, while the other subgroup does not possess these features (Fig. 15).

#### ﻿ Novochares dentatus sp. nov.

Taxon classificationAnimaliaColeopteraHydrophilidae

0C78ECC3-05FE-5034-B24A-380AB2AE5A95

https://zoobank.org/6F12BE0D-118D-4A52-B342-43A8270270B9

**Figure 16.**
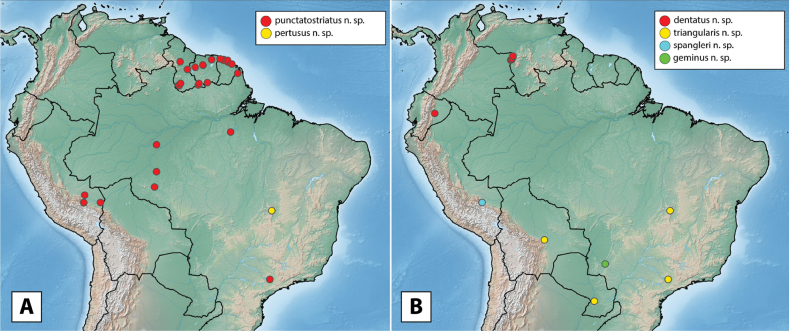
Distribution of *Novocharespunctatostriatus* species group **A***N.punctatostriatus* (red), *N.pertusus* (yellow) **B***N.dentatus* (red), *N.triangularis* (yellow), *N.spangleri* (blue), *N.geminus* (green).

Figs 4A , 14A , 15A–D , 16B

##### Type material.

***Holotype* (male)**: “VENEZUELA: Amazonas State/ 5°20.514'N, 67°45.315'W, 87m/ S. Communidad Porvenir/ 15.i.2009; leg. Miller & Short/ VZ09-0115-03B/ small streamlet (MIZA). ***Paratypes* (57 exs.): Venezuela: Amazonas**: 5 km N. Galipero, Pozo Azul, 25.i.1989, leg. Spangler, Faitoute, & Barr, “roots, stream edge” (1, USNM); same data as holotype (25, MIZA, SEMC, including DNA Voucher SLE1199); ca. 15 km S. Puerto Ayacucho, large rock outcrop, 14.ix.2007, leg. Short, pools at base of outcrop, AS-07-011a (2, SEMC); same locality but 8.viii.2008, leg. Short & García, pools at base of outcrop, AS-08-081a (4, SEMC); same locality but 14.i.2009, leg. Short, “rock pools et al”, VZ09-0114-03B (5, SEMC); Tobogan de la Selva, 5.i.2006, leg. Short, pools in rock w/sand, AS-06-011c (1, SEMC); nr. Iboruwa, 7.viii.2008, leg. Short, García, & Joly, AS-08-078 (6, SEMC); Puerto Ayacucho (39 km S.), Samariapo road, 15.xi.1987, leg. Spangler & Faitoute, “brook”, Collection #4 (1, USNM). **Ecuador: Pastaza**: AGIP platform Villano B, along transect 1 and 2, 24.v.2008, leg. Short, small forest stream, AS-08-008b (12, PUCE, SEMC, including DNA voucher SLE1188).

##### Differential diagnosis.

The distinctive rows of serial punctures on the lateral and posterior margins of the elytra serve to separate this species from all other *Novochares* except a few others in the *punctatostriatus* species group, particularly *N.punctatostriatus* which also occurs throughout much of the Amazonian region. However, as far as is known, the ranges of the two species do not overlap, with *N.dentatus* being more northern and western in distribution. The two species can be distinguished by the impression of the rows of serial punctures, which are more impressed and prominent in *N.punctatostriatus*, as well as the presence of small denticles on the aedeagus (Fig. 15A; denticles absent in *N.punctatostriatus*, e.g., Fig. 15L).

##### Description.

**Size and form**: Body length 4.7–6.5 mm. ***Coloration***: Dorsal surfaces dark brown, with slightly paler margins of pronotum and elytra, occasionally paler clypeus; paler margin sometimes very wide. ***Head***: Maxillary palps as long as to slightly longer than width of head, uniformly orange in color (Fig. 14A). ***Thorax***: Each elytron with defined rows of serial punctures along posterior 1/3 and lateral 1/2. Prosternum medially weakly and broadly convex. Posterior elevation of mesoventrite transverse, low, and slightly curved (posteriorly concave). ***Aedeagus***: (Figs 4A, 15A–D) lateral projection on apical region of outer margin of each paramere rounded to pointed; at closest point, dorsal inner margins of parameres separated by distance similar to greatest width of a paramere; dorsal plate of median lobe with small denticle on each lateral margin, proximal to base of fork; arms of dorsal plate of median lobe parallel to distally converging; each arm parallel sided, slightly wider on apical region, apically oblique with inner margin extending beyond outer margin; notch between arms at base nearly as broad as base of an arm; ventral plate of median lobe gradually more sclerotized distally, at widest point nearly as wide as maximum width of ventral face of a paramere, apically acuminate, apex extending beyond base of fork, not reaching apex of arms of dorsal plate, ventral surface of each side medially sharply elevated forming narrow medial slit; basal piece 0.3× length of a paramere.

##### Etymology.

*Dentatus* (L.), meaning toothed, in reference to the small, lateral, tooth-like projections on the dorsal plate of the median lobe.

##### Distribution.

Venezuela, Ecuador (Fig. 16B).

##### Habitat.

In Venezuela, this species was typically found along the margins of streams that were flowing on or near granite outcrops. The series from Ecuador was taken from a small forested stream with lots of detritus, though it did not appear to be associated with any rocky substrate.

##### Remarks.

Specimens from Ecuador tend to be smaller and more yellowish than specimens from Venezuela (specimens from the “Tobogancito” locality are similar in size to specimens from Ecuador). The eyes of specimens from Venezuela are relatively larger and more prominent than those of specimens from Ecuador.

#### ﻿ Novochares geminus sp. nov.

Taxon classificationAnimaliaColeopteraHydrophilidae

2D22DEBB-4506-5CD1-ABD5-735733333C82

https://zoobank.org/1CA1D279-2185-4BA5-93EF-BAF6E60FB642

Figs 15G, H , 16B

##### Type material.

***Holotype* (male)**: “BRAZIL: Mato Grosso do Sul/ -20.72281, -55.69127; 225 m/ Aquidauana (c. 27 m S) on/ MS-174; leg. Hamada & team;/27.vi.2018; seepage & debris nr. stream margin; BR18-0627-01E” (INPA). ***Paratypes* (2 exs.): Brazil: Mato Grosso do Sul**: Same data as holotype (2, SEMC, including DNA Voucher SLE2092).

##### Differential diagnosis.

Among species of the *punctatostriatus* species group, *N.geminus* is one of three species that lack distinct rows of elytral serial punctures, the others being *N.spangleri* and *N.triangularis*. Those two species can be separated by the shape of the forked projections of the median lobe, which are broader and swollen at the apex (Fig. 15 E, F, I), while they are slender and not expanded apically in *N.geminus* (Fig. 15G, H).

##### Description.

**Size and form**: Body length 5.9–6.0 mm. ***Coloration***: Dorsal surfaces dark brown, with slightly paler margins of pronotum and elytra. ***Head***: Maxillary palps as long as to slightly longer than width of head, uniformly orange in color. ***Thorax***: Elytra without defined rows of serial punctures, each with one dorsal and a few lateral sparse rows of systematic punctures. Prosternum medially weakly and broadly convex. Posterior elevation of mesoventrite transverse, low, and blunt. ***Aedeagus***: (Fig. 15G, H) lateral projection on apical region of outer margin of each paramere pointed; at closest point, dorsal inner margins of parameres separated by distance nearly 1/2 greatest width of a paramere; dorsal plate of median lobe with small denticle on each lateral margin, proximal to base of fork; arms of dorsal plate of median lobe parallel, diverging at apex; each arm parallel sided, with inner margins sinuate, apex rounded and pointing outwards; notch between arms at base nearly as broad as base of an arm; ventral plate of median lobe lightly sclerotized, at widest point nearly as wide as dorsal plate of median lobe, apically acuminate, apex nearly reaching apex of arms of dorsal plate, ventral surface of each side medially sharply elevated forming narrow medial slit; basal piece 0.35× length of a paramere.

##### Etymology.

*Geminus* (L.), meaning twin, in reference to the split apex of the dorsal plate of the median lobe.

##### Distribution.

Brazil (Mato Grosso do Sul) (Fig. 16B).

##### Habitat.

The only known series was taken from the margin of a rocky stream, where a seep was flowing over large rocks.

#### ﻿ Novochares pertusus sp. nov.

Taxon classificationAnimaliaColeopteraHydrophilidae

320E7EF6-8E75-5652-863F-1DBB7C2B7402

https://zoobank.org/FF90FFBC-6E9A-4907-B15C-09737EA3E48D

Figs 6D , 14C , 15J, K , 16A

##### Type material.

***Holotype* (male)**: “BRAZIL: Goiás/ Chapada dos Veadeiros/ 18–24km N. of Alto Paraíso/ 1400–1500m, 25.x.1985 leg. S.E. Miller” (USNM). ***Paratypes* (14 exs.): Brazil: Goiás**: Same data as holotype (14, USNM, SEMC).

##### Differential diagnosis.

The distinctive rows of serial punctures on the lateral and posterior margins of the elytra serve to separate this species from all other *Novochares* except a few others in the *punctatostriatus* species group. It is most similar to *N.punctatostriatus*, to which it is probably most closely related, by the form of the aedeagus which lacks small lateral teeth on the dorsal plate of the median lobe (Fig. 15J–Q). The comparatively long arms of the fork of the dorsal plate of the median lobe, and the more basal positioning of the ventral plate (Fig. 15J, K) serve to distinguish this species from *N.punctatostriatus* (Fig. 15L–Q).

##### Description.

**Size and form**: Body length 6.0–7.3 mm. ***Coloration***: Dorsal surface of head very dark brown to nearly black; pronotum and elytra dark brown, with broad paler margins. ***Head***: Maxillary palps as long as to slightly longer than width of head, uniformly orange in color (Fig. 14C). ***Thorax***: Elytra with defined rows of serial punctures, except on central elytral disc. Prosternum medially broadly and very weakly convex. Posterior elevation of mesoventrite transverse, blunt, and low. ***Aedeagus***: (Fig. 15J, K) lateral projection on apical region of outer margin of each paramere pointed; at closest point, dorsal inner margins of parameres separated by distance slightly narrower than greatest width of a paramere; lateral margins of dorsal plate of median lobe smooth, lacking denticles; arms of dorsal plate of median lobe nearly parallel, somewhat converging at apex; each arm with outer and inner margins slightly sinuate, apical region broader than base of arm, apex roundly truncate and oblique, with inner margin extending beyond outer margin; notch between arms at base narrower than base of an arm, slightly wider at basal 1/3, narrowing towards apex; ventral plate of median lobe strongly sclerotized, at widest point nearly as wide as dorsal plate of median lobe, with apex narrowly rounded, not reaching base of fork of dorsal plate; basal piece 0.28× length of a paramere.

##### Etymology.

*Pertusus* (L.), meaning perforated, in reference to the rows of fine serial punctures on the elytra.

##### Distribution.

Only known from the type locality in Brazil (Goiás) (Fig. 16A).

##### Habitat.

Nothing is known about the habitat of this species.

#### ﻿ Novochares punctatostriatus sp. nov.

Taxon classificationAnimaliaColeopteraHydrophilidae

0F00FF58-3318-5C05-B346-25499933A86E

https://zoobank.org/DFD9C645-A8FD-4F66-811B-06EFEA44E991

Figs 14B , 15L–Q , 16A

##### Type material.

***Holotype* (male)**: “PERU: Madre de Dios/ Rio Tambopata Res.; 290m/ 30 air km SW of Puerto Maldonado/ 16–20.xi.1979; subtropical moist forest/ leg. J.B. Heppner” (USNM). ***Paratypes* (262 exs.): Brazil: Amapá**: Oiapoque (ca. 22 km S) on BR-156, leg. Short, forested detrital pools, BR18-0720-01B (1, SEMC, including DNA Voucher SLE2094). **Amazonas**: Tapauá, Humaita (ca. 240 km N) on BR-319, -5.50298, -62.12392, 54 m, 11.vii.2018, leg. Short, margin of stream, BR18-0712-01B (2, INPA, SEMC, including DNA Voucher SLE1969). **Pará**: Altamira (ca. 60 km S), Rio Xingu Camp, 52°40'W, 3°50'S, 12.x.1986, leg. P. Spangler & O. Flint (10, USNM). **Rondônia**: Machadinho d’Oeste, Tabajara (ca. 7.5 km W) on RO-133, -8.92368, -62.12491, 82 m, 8.vii.2018, leg. Short, river with sandy bottom and rocks, BR18-0708-04A (1, SEMC, including DNA Voucher SLE2037); Novo Uniao, Vale do Cachoeiras, -10.91764, -62.377, 359 m, 10.vii.2018, leg. Short, small sandy-bottom stream margin, BR18-0710-02A (1, SEMC, including DNA Voucher SLE2090). **São Paulo**: Piracicaba, dates between 10.ii.1965 and 2.xii.1965, blacklight, C.A. Triplehorn (68, USNM). **French Guiana**: Route de Petit Saut, Crique Maman Lézard, 5.06701°N, -52.99783°W, 39 m, 1.iii.2020, leg. Short & Neff, margin of creek with detritus, FG20-0301-01A (1, SEMC); Same data but detrital pools in drying creakbed, FG20-0301-01B (1, SEMC); Carbet ONF Montagne de fer, Piste de montagne de fer (formerly road Degrad Florian), Crique Petit Laussat, 5.40697°N, -53.55468°W, 10 m, leg. Short, detrital pools, FG20-0302-01C (3, SEMC); Piste de montagne de fer (formerly Degrad Florian road), tributary of Crique Florian, 5.29688°N, -53.52458°W, 25 m, leg. Short & Neff, small pools in stream channel with sand and detritus, FG20-0303-01A (3, SEMC); same data but leg. Short, margin of clearwater creek, FG20-0303-01B (2, SEMC); St. Laurent du Maroni, Sentier des Malgaches, 5.48627°N, -54.00238°W, 14 m, leg. Short & Neff, pond margins in secondary forest, FG20-0304-01A (1, SEMC); Piste de montagne de fer (formerly road Degrad Florian), 5.40697°N, -53.55468°W, 10 m, leg. Short & Neff, forested detrital pools, FG20-0305-01A (7, SCC, SEMC); Carbet communal St-Elie, Route de Saint-Elie, tributary of Crique Toussaint, 5.29653°N, -53.05205°W, 42 m, leg. Short & Neff, margins of clearwater stream, FG20-0305-03B (2, SEMC); Paracou, Station de recherche CIRAD, Crique Verlot, 5.27966°N -52.92846°W, 8 m, leg. Short & Neff, forested detrital pools, FG20-0306-01A (1, SEMC); Bagne des Annamites Park, Crique Anguille, 4.83287°N, -52.5145°W, 17 m, leg. Short & Neff, small sandy stream with detritus, FG20-0307-01B (2, SEMC). **Guyana: Region 6**: Upper Berbice circa 1 km west of Basecamp 1, 4°09.143'N, 58°11.207'W, 105 m, 22.iv.2014, leg, Short, Salisbury and La Cruz, margins of creek, GY14-0921-03H (1, SEMC). **Region 8**: Konawaruk River, Basecamp 2 (NARIL camp), 14.ix.2014, leg. Salisbury & La Cruz, small puddle along road, GY14-0914-03 (1, SEMC); Konawaruk River, Basecamp 2 (NARIL basecamp), 5°07.539'N, 59°06.732'W, 80 m, 15.ix.2014, leg. Salisbury and La Cruz, unnamed clear water creek, slow flowing and shallow, GY14-0915-02 (1, SEMC). **Region 9**: along road to Parabara, 2°09.557'N, 59°17.569'W, 268 m, 1.xi.2013, leg. Short, Isaacs and Salisbury, forest pools near Mushai Wao, GY13-1101-02A, (2, SEMC); Parabara, trail to mines, 2°05.095'N, 59°14.174'W, 250 m, 2.xi.2013, leg. Short, Isaacs and Salisbury, detrital pools in forest, GY13-1102-01A, (1, SEMC); Parabara north side of river, 2°06.492'N, 59°13.653'W, 274 m, 3.xi.2013, detritus margins and leaf packs, GY13-1103-02A (1, SEMC); Karaawaimin Taawa, Basecamp and surroundings, 2.42284N, 59.06157W, 11-13.iii.2022, leg. Short & Edward, detrital pools near camp, GY22-0311-01A (1, SEMC); pooled up sandy creek GY22-0311-01D (2, SEMC); Karaawaimin Taawa, Trail from Camp 1 to Camp 2, 14.iii.2022, leg. Short & Edward, palm swamp, GY22-0314-04A (4, SEMC); Karaawaimin Taawa, Camp 2 and surroundings, 15.iii.2022, leg. Short & Edward, stream with palm detritus, rocks, and sand, GY22-0315-01A (1, SEMC); Karaawaimin Taawa, Camp 3 and surroundings, 16-18.iii.2022, leg. Short & Edward, small pool in streambed, GY22-0316-01D (1, SEMC); same data but forest pools, GY22-0316-01C (3, SEMC); Karaawaimin Taawa, Camp 4 and surroundings, 18-21.iii.2022, leg. Short & Edward, small stream, GY22-0318-01C (8, SEMC); same data but pools in creekbed, GY22-0318-01D (8, SEMC); same data but second small stream, GY22-0318-01E (18, CBDG, SEMC); Karaawaimin Taawa, Trail between Camps 3 and 4, 21.iii.2022, leg. Short & Edward, small pool in forest, GY22-0321-01B (3, SEMC). **Peru: Cuzco**: Pilcopata, 600 m, 8-10.xii.1979 premonate moist forest, leg. J.B. Heppner (3, USNM); Pilcopata (ca. 3 km NE), on nearby mountain road, 30.v.2022, leg. Short et al., pools and roadside ditches, PE22-0530-01B (1, SEMC); same data but small stream and adjacent grassy pool, PE22-0530-01C (1, SEMC). **Madre de Dios**: same data as holotype (22, USNM, SEMC); Manu Pakitza, 12°7'S, 10°58'W, 250 m, 18.viii.1988, UV light, leg. O. Flight & N. Adams (1, USNM); same locality but 14–23.ix.1988, malaise traps, “trail 2, 1^st^ stream” (1, USNM); Amazonas Lodge, N Atalaya, 12°52.2'S, 71°22.6'W, 480 m, 10-13.xi.2007, leg. D. Brzoska, flight intercept trap, PER1B07 002 (2, SEMC); Villa Carmen Biological Station (ca. 2 km N of Pilcopata), South of Rio Piñipiñi, 26.v.2022, leg. Short et al., small streams in bamboo thicket, PE22-0526-01A (1, SEMC); same data but large marshy pool along trail with abundant detritus, PE22-0526-01E (1, SEMC); Villa Carmen Biological Station (ca. 2 km N of Pilcopata), North of Rio Piñipiñi, 28.v.2022, leg. Short et al., small detrital pools, PE22-0526-02F (3, MHNSM, SEMC). **Suriname: Saramacca**: Coesewijne Savanna, 6.iii.2012, leg. Short, forested pool in muddy road, SR12-0306-03B (9, SEMC). **Sipaliwini**: Camp 1 on Kutari River, 2°10.521'N, 56°47.244'W, 228 m, 20.viii.2010, leg. Short and Kadosoe, forest stream, CI-RAP Survey, forested swamp, SR10-0819-01A (7, SEMC); Iwaana Saamu, forest swamp, 26.viii.2010, leg. Short, SR10-0826-01A (1, SEMC); Camp 3, Werehpai, 2°21.776'N, 56°41.861'W, 237 m, 3-7.ix.2010, leg. Short and Kadosoe, pooled up detrital creek, SR10-0903-01A (3, SEMC, including DNA Voucher SLE452); same data except detrital forest pools, SR10-0903-02A (1,SEMC); same data except sandy forest creek, SR10-0904-01A (3, SEMC); Upper Palumeu, Camp 1, 2.47700°N, 55.62941°W, 275 m. leg. A. Short, 10–16.iii.2012, Flight Intercept Trap, SR12-0310-TN1 (3 SEMC); Raleighfallen Nature Reserve, trail to Raleighfallen, 04°42.480'N, 56°13.159'W, 24 m, 27.vii.2012, SR12-0727-03A (1, SEMC); same data but leg. C. McIntosh, detrital pools near creek in forest, SR12-0727-03D (2, SEMC); Raleighvallen Nature Reserve Voltzberg trail, 04°40.910'N, 56°11.138'W, 78 m, 30.vii.2012, SR12-0730-01A (1, SEMC); same data but detrital pools along stream, SR12-0730-01B (2, SEMC); Raleighfallen Nature Reserve, Fungu island, 04°43.459'N, 56°12.658'W, 30 m, 1.viii.2012, SR12-0801-01D (2, SEMC); Raleighvallen Nature Reserve, base of Voltzberg, 4°40.432’N, 56°11.079’W, 86 m, 16.iii.2016, leg. Short et al., pooled up stream, SR16-0316-01B (2, SEMC); Raleighvallen Nature Reserve, Lolopaise area, 4°42.48'N, 56°13.15908'W, 24 m, intermittent stream pools, 19.iii.2016, leg. Toussaint et al., SR16-0319-02C (1, SEMC); Raleighvallen Nature Reserve, Coppename River, Voltzberg trail, 17.iii.2016, leg. A. Short, detrital pools in stream bed, SR16-0319-01A (10, NZCS, SEMC); Kabalebo Nature Resort, Moi Moi Creek, leg. Short, detrital pool, SR19-0310-01G (3, SEMC, including DNA Voucher SLE1802); same data except leg. Short and class, SR19-0310-01M (2, SEMC). **Suriname**: Krakka-Phedra Road, 25.x.1962, leg. B. Malkin (12, USNM).

##### Differential diagnosis.

The distinctive rows of serial punctures on the lateral and posterior regions of the elytra serve to separate this species from all other *Novochares* except a few others in the *punctatostriatus* species group. This species is the most commonly encountered and widespread of the six known species in the group, occurring from the eastern Guiana Shield (Guyana, Suriname), west to the foothills of the Peruvian Andes, and south São Paulo, Brazil. It is also one of the largest species in the group. The lack of lateral denticles on the dorsal plate of the median lobe separates this from other *punctatostriatus* species group taxa except for *N.pertusus* sp. nov. (see diagnosis of that species).

##### Description.

**Size and form**: Body length 5.2–8.8 mm. ***Coloration***: Dorsal surface of head dark brown, sometimes with gradually paler clypeus and labrum; pronotum and elytra dark brown, with broad pale margins. ***Head***: Maxillary palps as long as to slightly longer than width of head, uniformly orange in color (Fig. 14B). ***Thorax***: Ground punctation on pronotum and elytra relatively dense and shallowly impressed. Elytra with defined rows of serial punctures, except on central elytral disc. Prosternum flat to medially weakly and broadly convex. Posterior elevation of mesoventrite transverse, blunt, and low. ***Aedeagus***: (Fig. 15L–Q) lateral projection on apical region of outer margin of each paramere pointed and strongly protruded; at closest point, dorsal inner margins of parameres separated by distance slightly narrower than greatest width of a paramere; lateral margins of dorsal plate of median lobe smooth, lacking denticles; arms of dorsal plate of median lobe slightly converging at apex; each arm with outer and inner margins straight at base, curved at apex (outer margin concave, inner margin convex); apical region of arm broader than base of arm, apex broadly rounded and oblique, with inner margin extending beyond outer margin; notch between arms very narrow, much narrower than base of an arm, narrowing even more towards apex; ventral plate of median lobe moderately sclerotized, at widest point seemingly wider than dorsal plate of median lobe, with apex broadly to narrowly rounded, reaching to slightly surpassing base of fork of dorsal plate; basal piece 0.35× length of a paramere.

##### Etymology.

*Puctatostriatus* (L.) in reference to the distinct rows of elytral serial punctures.

##### Distribution.

Brazil (Amapá, Amazonas, Rondônia, São Paulo), French Guiana, Guyana, Peru, and Suriname (Fig. 16A).

##### Habitat.

This species is most commonly found in the detrital margins of densely forested lowland streams. Some collections have also been made in forested detrital pools, especially those that are riparian in origin. It has also been collected at lights and in malaise traps.

##### Remarks.

This relatively distinct and widespread taxon is very likely a complex of very closely related species. The pairwise genetic divergence in COI is nearly 9% among the ten individuals we sequenced from Peru to Suriname and French Guiana. This is the largest observed intraspecific divergence among any *Novochares* examined here, though not unprecedented in Acidocerinae: both *Helocharesmaculicollis* Mulsant, 1844 and *H.normatus* (LeConte, 1861) showed intraspecific variation of greater than 9% (Short and Girón 2018) but these too also likely represent species complexes. We examined the aedeagus from a variety of populations, and indeed there are subtle variations. The apex of the ventral plate of the median lobe varies in length and shape. The width of the notch between the arms of the dorsal plate of the median lobe can be very narrow and be essentially not visible to be as broad as 1/4 the width of an arm at base. However, we were not able to correlate this variation with any structure in the molecular tree. We have taken a conservative approach, and favor treating all these populations as one widespread, somewhat variable species.

#### ﻿ Novochares spangleri sp. nov.

Taxon classificationAnimaliaColeopteraHydrophilidae

8BAAC044-3CFD-550B-AFE4-AA4E9F00AB80

https://zoobank.org/59377017-2936-4FA3-9B74-154A5D09E5E5

Figs 15E–F , 16B

##### Type material.

***Holotype* (male)**: “PERU: Cusco: Paucartambo/ -12.91411S, -71.37492W, 585m/ c. 3km NE of Pilcopata; 30.v.2022/ grassy pools/ditch along road/ PE22-0530-01C; leg. Short et al.” (MHNSM). ***Paratypes* (6 exs.): Peru: Cusco**: same data as holotype (1, SEMC); same data except mountain side pools and ditches, PE22-0530-01B (2, SEMC). **Madre de Dios**: Villa Carmen Biological Station (ca. 2 km N of Pilcopata), North of Rio Piñipiñi, 26.v.2022, leg. Short et al., small muddy pools in landslide, PE22-0526-02B (1, SEMC); same data except 28.v.2022, detrital pools formed by seeps, PE22-0526-02F (2, MHNSM, SEMC).

##### Differential diagnosis.

Among members of the *punctatostriatus* species group, *N.spangleri* is one of three species that lack distinct rows of elytral serial punctures, the others being *N.pertusus* and *N.triangularis*. It is most similar to *N.triangularis*, to which the aedeagal form is very similar, though it can be distinguished by the apices of the forked projection of the dorsal plate of the median lobe being slightly more swollen, with the outer margins of the parameres weakly curved (apices of the forked projection of the dorsal plate of the median lobe being not as swollen and the outer margins of the parameres strongly curved in *N.triangularis*; compare Fig. 15E vs. Fig. 15I). The elytral ground punctation is extremely fine and almost not noticeable in *N.spangleri*, while it is distinctly coarser in *N.triangularis*. Additionally, *N.triangularis* has only very narrow paler margins of the pronotum, these margins are much more broadly pale in *N.spangleri*. See diagnosis of *N.pertusus* for separation from that species.

##### Description.

Body length 5.6–6.0 mm. ***Coloration***: Dorsal surface of head, labrum, pronotum, and elytra dark brown, gradually paler towards margins. ***Head***: Maxillary palps slightly shorter than width of head, uniformly yellow in color. ***Thorax***: Ground punctation on pronotum and elytra relatively dense and very shallowly impressed. Elytra without rows of serial punctures, except for rows of very weak serial punctures along lateral regions of each. Prosternum medially convex. Posterior elevation of mesoventrite transverse, blunt, and low. ***Aedeagus***: (Fig. 15E, F) lateral projection on apical region of outer margin of each paramere rounded to weakly pointed; at closest point, dorsal inner margins of parameres separated by distance slightly greater than greatest width of a paramere; dorsal plate of median lobe with small denticle on each lateral margin, proximal to base of fork; arms of dorsal plate of median lobe distally weakly converging; each arm slightly wider at base and widened on apical region, apically oblique with inner margin extending beyond outer margin; notch between arms at base 1/2 as wide as base of an arm; ventral plate of median lobe moderately sclerotized, at widest point as wide as dorsal plate of median lobe, apically acuminate, apex extending beyond base of fork, not reaching apex of arms of dorsal plate; basal piece 0.4× length of a paramere.

##### Etymology.

Named after Paul J. Spangler, longtime curator at the US National Museum of Natural History, Smithsonian Institution, and specialist on aquatic beetles, who collected and sorted a lot of the specimens included in this contribution.

##### Distribution.

Peru (Fig. 16B).

##### Habitat.

This species was collected primarily in forested riparian habitats.

#### ﻿ Novochares triangularis sp. nov.

Taxon classificationAnimaliaColeopteraHydrophilidae

0E6A8311-432C-5FAD-82C6-43C19F2E3237

https://zoobank.org/4281F5F3-075C-4259-A3A2-59243165F5CD

Figs 14D , 15I , 16B

##### Type material.

***Holotype* (male)**: “BOLIVIA: Santa Cruz Dept./Potrerillos del Guenda,/Preserva Natural, 17°40'S, 63°27'W, 370m, 17-22-X-2007/ ex BL/MV, J. & F. Romero/ BOL1Cline07 007.5” (SEMC). ***Paratypes* (25 exs.): Brazil: Goiás**: Chapada dos Veadeiros, 18–24 km. N. of Alto Paraíso, 1400–1500 m, 25.x.1985, leg. S.E. Miller (5, USNM). **Minas Gerais**: Pedra Azul, 800 m, xi.1972, leg. M. Alvarenga (2, CMNH). **São Paulo**: Piracicaba, 12.xii.1965, leg. C.A. Triplehorn (10, USNM). **Bolivia: Santa Cruz**: Same data as holotype except leg. A.R. Cline & J. Wappes, “BOL1Cline07 007” (1, SEMC). **Paraguay: Cordillera**: Compania Naranjo, 266 m, leg. Brzoska; 2.xii.2012 (9, SEMC).

##### Differential diagnosis.

See differential diagnoses of *N.geminus* and *N.spangleri*.

##### Description.

Body length 5.4–6.7 mm. ***Coloration***: Dorsal surfaces very dark brown, with paler margins of pronotum and elytra. ***Head***: Maxillary palps as long as to slightly longer than width of head, uniformly orange in color (Fig. 14D). ***Thorax***: Ground punctation on pronotum and elytra relatively dense and shallowly impressed. Elytra without defined rows of serial punctures, each with one dorsal and a few lateral sparse rows of systematic punctures; rows of systematic punctures more evident along posterior region. Prosternum sometimes medially convex. Posterior elevation of mesoventrite transverse, low, and slightly curved (posteriorly concave). ***Aedeagus***: (Fig. 15I) lateral projection on apical region of outer margin of each paramere rounded to weakly pointed; at closest point, dorsal inner margins of parameres separated by distance similar to greatest width of a paramere; dorsal plate of median lobe with small denticle on each lateral margin, proximal to base of fork; arms of dorsal plate of median lobe distally converging; each arm nearly parallel sided, slightly wider on apical region, apically oblique with inner margin extending beyond outer margin; notch between arms at base slightly narrower than base of an arm; ventral plate of median lobe weakly sclerotized, at widest point nearly as wide as dorsal plate of median lobe, apically acuminate, apex extending beyond base of fork, not reaching apex of arms of dorsal plate; basal piece 0.4× length of a paramere.

**Figure 17.**
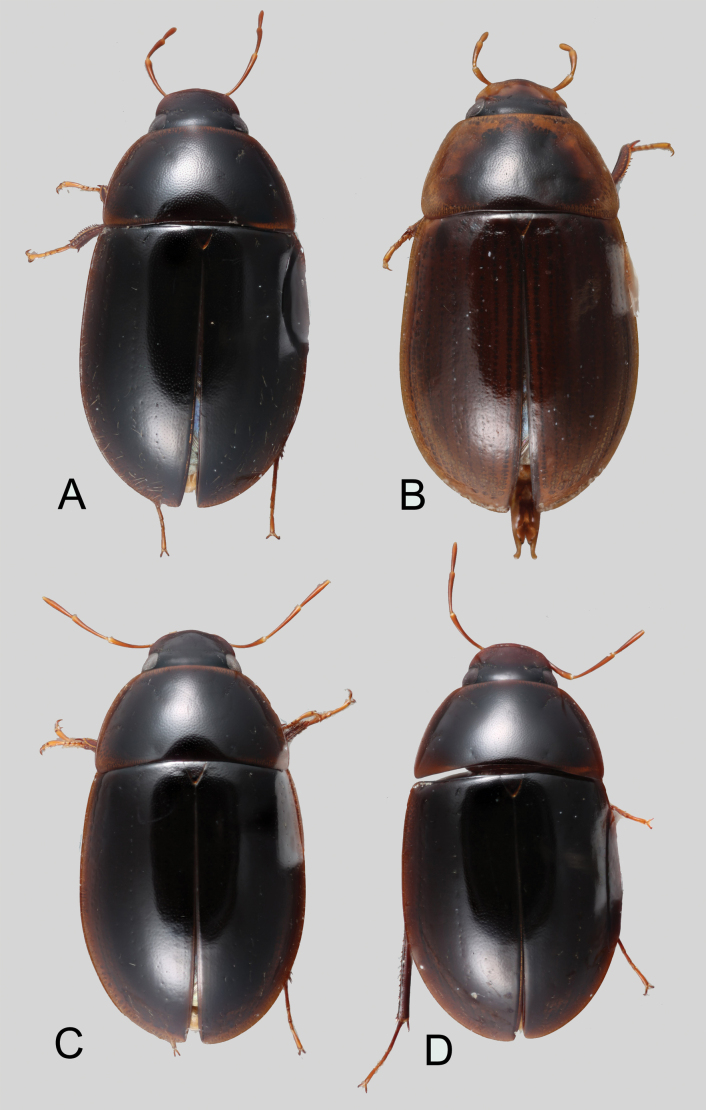
Dorsal habitus of *Novochares* spp. **A***N.guadelupensis***B***N.pichilingue***C***N.cochlearis***D***N.quadrispinus*.

##### Etymology.

*Triangularis* (L.), in reference to the triangular shape of the ventral plate of the median lobe.

##### Distribution.

Bolivia, Brazil (Goiás, Minas Gerais, São Paulo), Paraguay (Fig. 16B).

##### Habitat.

Little is known about the habitat of this species, there is no ecological information on the labels. Some specimens were collected at lights.

###### ﻿*Novocharessallaei* species group

**Figure 18.**
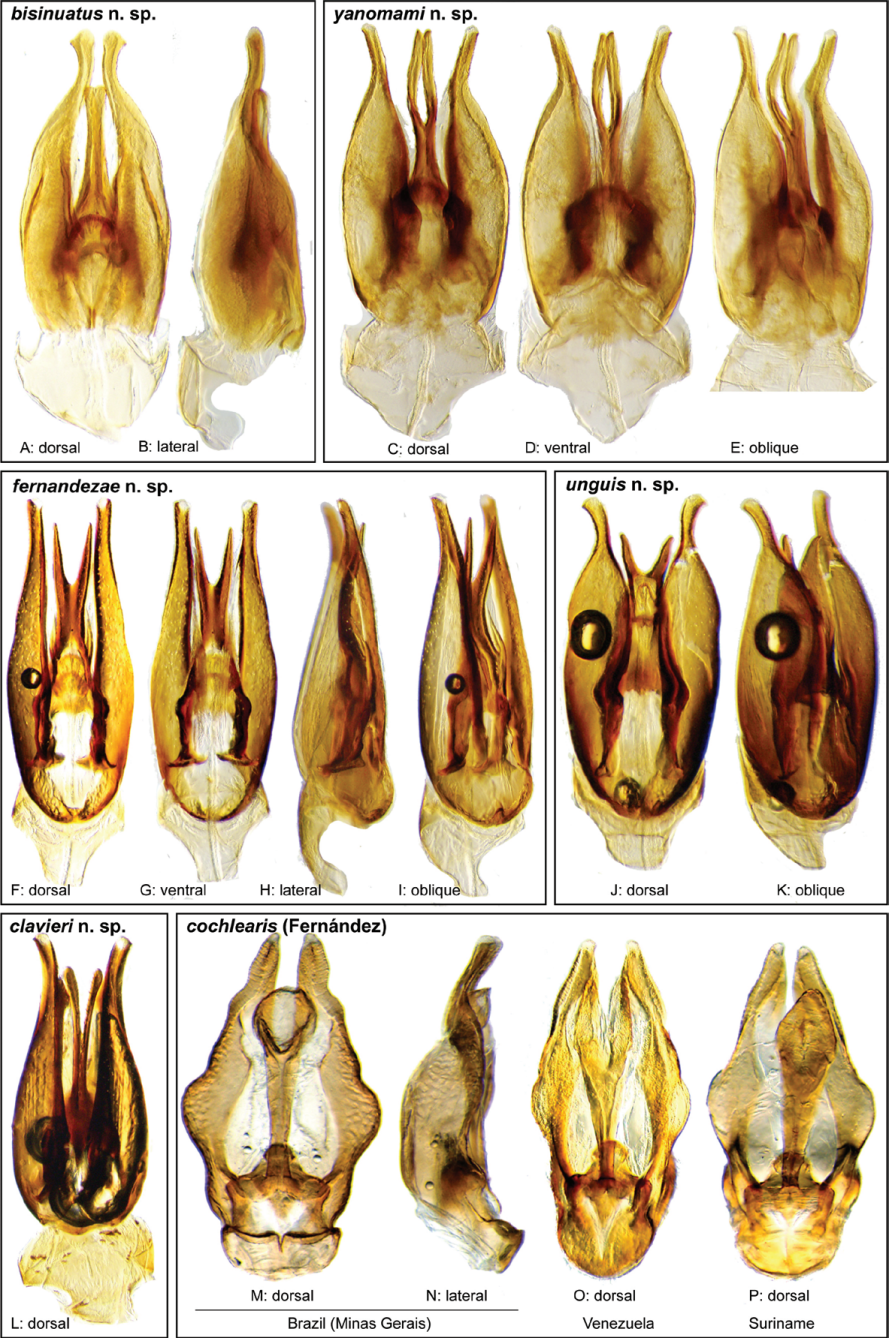
Aedeagi of the *Novocharessallaei* species group **A, B***N.bisinuatus***C–E***N.yanomami***F–I***N.fernandezae***J, K***N.unguis***L***N.clavieri***M–P***N.cochlearis***A, C, F, J, L, M, O, P** dorsal view **B, H, N** lateral view **D, G** ventral view **E, I, K** oblique view **M, N** Brazil **O** Venezuela **P** Suriname.

**Figure 19.**
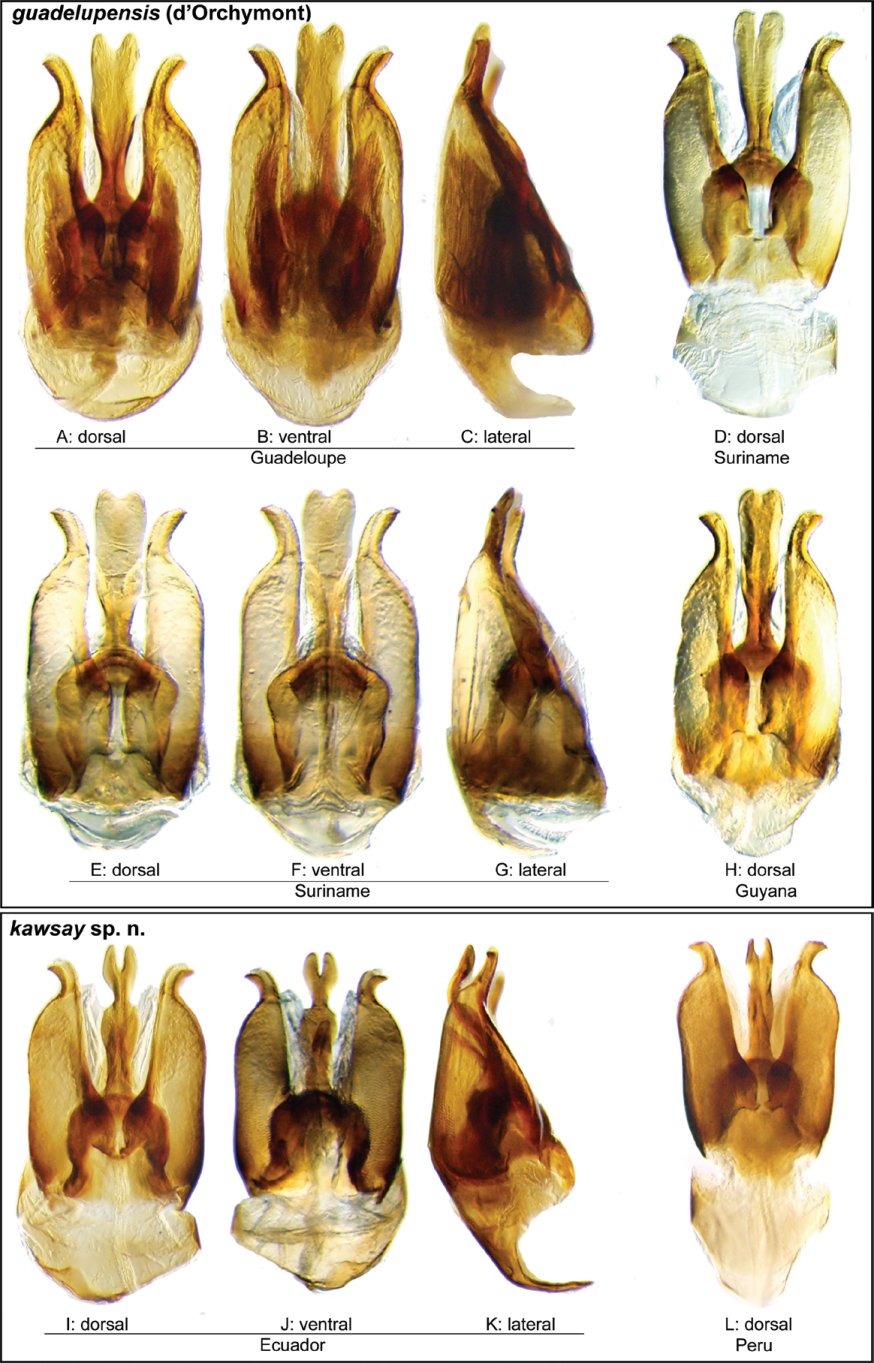
Aedeagi of the *Novocharessallaei* species group **A–H***N.guadelupensis***I–L***N.kawsay***A–C** Guadeloupe **D–G** Suriname **H** Guyana **I–K** Ecuador **L** Peru **A, D, E, H, I, L** dorsal view **B, F, J** ventral view **C, G, K** lateral view.

**Figure 20.**
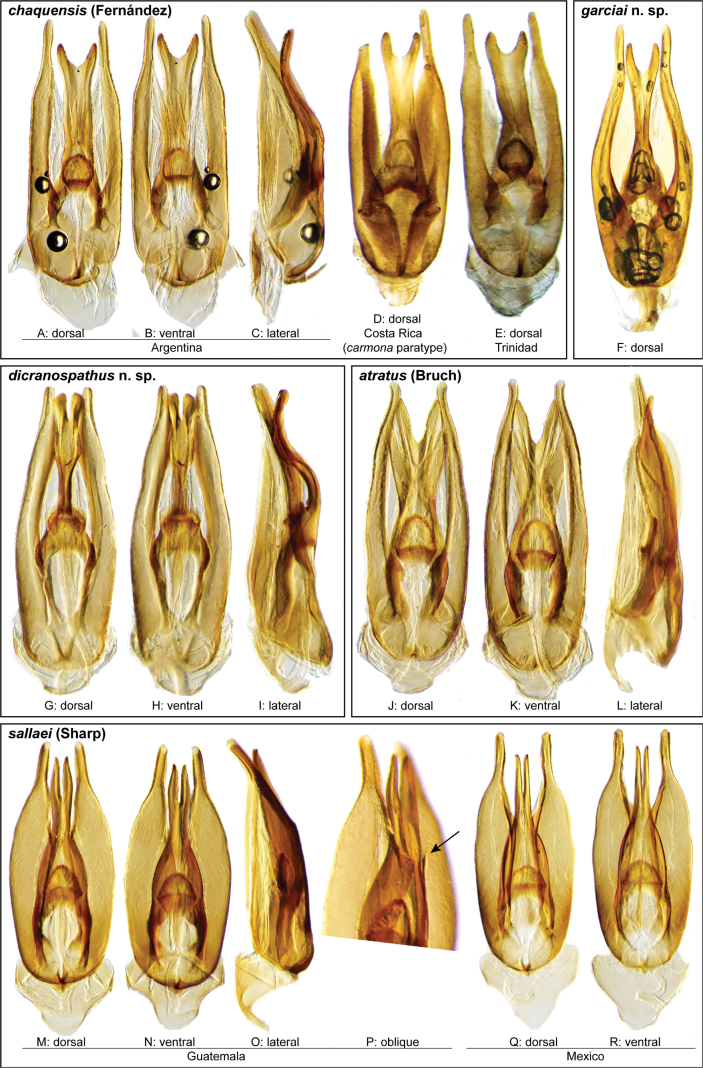
Aedeagi of the *Novocharessallaei* species group **A–E***N.chaquensis***F***N.garciai***G–I***N.dicranospathus***J–L***N.atratus***M–R***N.sallaei***A–C** Argentina **D** Costa Rica **E** Trinidad **M–P** Guatemala **Q, R** Mexico **A, D–G, J, M, Q** dorsal view **B, H, K, N, R** ventral view **C, I, L, O** lateral view **P** oblique view.

**Figure 21.**
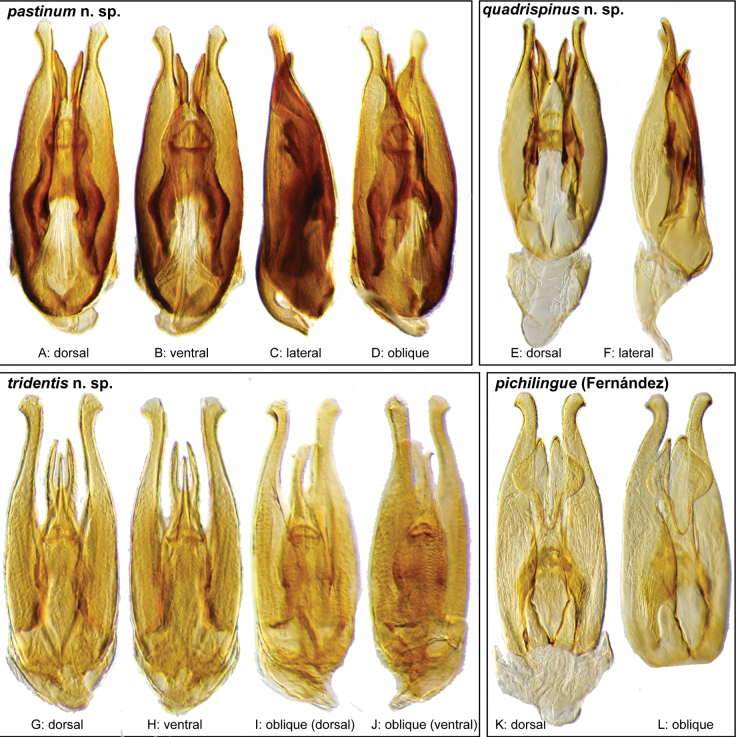
Aedeagi of the *Novocharessallaei* species group **A–D***N.pastinum***E, F***N.quadrispinus***G–J***N.tridentis***K, L***N.pichilingue***A, E, G, K** dorsal view **B** ventral view **C, F** lateral view **D, I** (dorsal) **J** (ventral) **L** oblique view.

**Species group diagnosis.** Body length 4.7–8.0 mm. ***Coloration***: Dorsal surfaces brown to dark brown or reddish brown. ***Aedeagus***: (Figs 18–21) Overall shape and relative length variable, joint basal margins of parameres broadly rounded, truncate or medially pointed; outer margin and apical region of each paramere variable; parameres usually longer than median lobe, with apex rounded or truncate; parameres with apical region variable in degree of sclerotization; dorsal inner margin of each paramere highly variable; dorsal plate of median lobe (in dorsal view) highly variable, sometimes forming a narrow neck; notch between arms variable; shape and orientation of arms variable; position of gonopore variable; ventral plate of median lobe (in ventral view) somewhat triangular, variable in length, shape of apex, and degree of sclerotization, sometimes absent; basal piece 0.20–0.55× length of a paramere, rarely strongly reduced (see *cochlearis*). In lateral view, aedeagus somewhat triangular, straight to strongly oblique at base, with ventral outline of parameres 1.5–4.2× longer than greatest width.

**Composition.** The *Novocharessallaei* species group is composed of 17 species: *N.atratus* (Bruch, 1915), *N.bisinuatus* sp. nov., *N.chaquensis* (Fernández, 1982) [= *N.carmona* (Short, 2005) syn. nov.], *N.clavieri* sp. nov., *N.cochlearis* (Fernández, 1982), *N.dicranospathus* sp. nov., *N.fernandezae* sp. nov., *N.garciai* sp. nov., *N.guadelupensis* (d’Orchymont, 1926), *N.kawsay* sp. nov., *N.pastinum* sp. nov., *N.pichilingue* (Fernández, 1989), *N.quadrispinus* sp. nov., *N.sallaei* (Sharp, 1882), *N.tridentis* sp. nov., *N.unguis* sp. nov., and *N.yanomami* sp. nov.

#### ﻿ Novochares atratus

Taxon classificationAnimaliaColeopteraHydrophilidae

(Bruch, 1915)

2DA6DBE7-0EA3-5DE3-A7B2-08E9CFEEF432

Figs 20J–L , 22B

Helochares atratus Bruch, 1915: 451.
Helochares (s. str.) atratus Bruch, 1915; Knisch 1925: 4; Fernández 1989: 148 [in key]; Clarkson and Ferreira Jr 2014: 400.
Helochares (s. str.) parhedrus d’Orchymont, 1939: 259 (synonymy: Fernández 1982a: 35).
Novochares atratus (Bruch, 1915); Girón and Short 2021: 203.

##### Type material examined.

***Holotype* (male)**: We examined images of the holotype, including the dissected aedeagus. The specimen is from Buenos Aires Province, Argentina and deposited in MACN.

##### Additional material examined

**(9 exs.): Argentina: Entre Ríos**: Río Paraná Ibicuy, Pto. Ibicuy, 10.xii.1979, leg. C.M. & O.S. Flint, Jr. (2, USNM). **Brazil: Bahia**: 5 km W. Ilheus, 4.vii.1969, leg. P. & P. Spangler (3, USNM). **Espírito Santo**: Muniz Freire, 19.vi.1908 (1, CMNH). **Rio de Janeiro**: Araruama, xi.1981, leg. Moacir Alvarenga (3, USNM).

##### Differential diagnosis.

The dorsal plate of the median lobe of the aedeagus is very unusual and distinct in that each side of the fork is bilobed and projected dorsally (Fig. 20J, L). The aedeagus is generally most similar to *N.chaquensis*, which may also be very dark brown to black.

##### Description.

Body length 6.0–7.2 mm. ***Coloration***: Dorsal surfaces brown to dark brown, usually with slightly paler (orange) clypeus, margins of head, pronotum, and elytra. ***Head***: Maxillary palps nearly 1.2× width of head, uniformly orange in color. ***Thorax***: Ground punctation on pronotum and elytra relatively dense and very shallowly impressed. Elytra without rows of serial punctures, each with very faint rows (one dorsal and two or three lateral) of scarce and weakly marked systematic punctures. Prosternum only very weakly medially convex. Posterior elevation of mesoventrite weakly, broadly, and somewhat triangularly elevated. ***Abdomen***: Apical emargination of fifth ventrite shallow to deep, U-shaped. ***Aedeagus***: (Fig. 20J–L) Overall shape pear-like, 4.5× longer than wide, with outer lateral margins of parameres weakly and evenly convex; apical region of each paramere rounded, with outer margin very weakly pointed; at closest point, dorsal inner margins of parameres separated by distance 0.6× greatest width of a paramere; dorsal plate of median lobe with neck 0.65× as broad as base; lateral margins of dorsal plate of median lobe strongly laterally projected at base of fork; arms of dorsal plate of median lobe diverging, dorsally concave, nearly 0.2× length of dorsal plate of median lobe; each arm uniformly wide along basal 1/2, then narrowing to acute apex; notch between arms at base nearly as wide as base of an arm; gonopore placed at base of dorsal plate of median lobe; ventral plate of median lobe weakly sclerotized, triangular, acute at apex, apex extending to base of neck of dorsal plate; basal piece 0.3× length of a paramere. In lateral view, aedeagus oblique at base, with ventral outline of parameres 3.4× longer than greatest width near base; dorsal outline of aedeagus in lateral view nearly straight along second 1/3.

##### Distribution.

**Figure 22.**
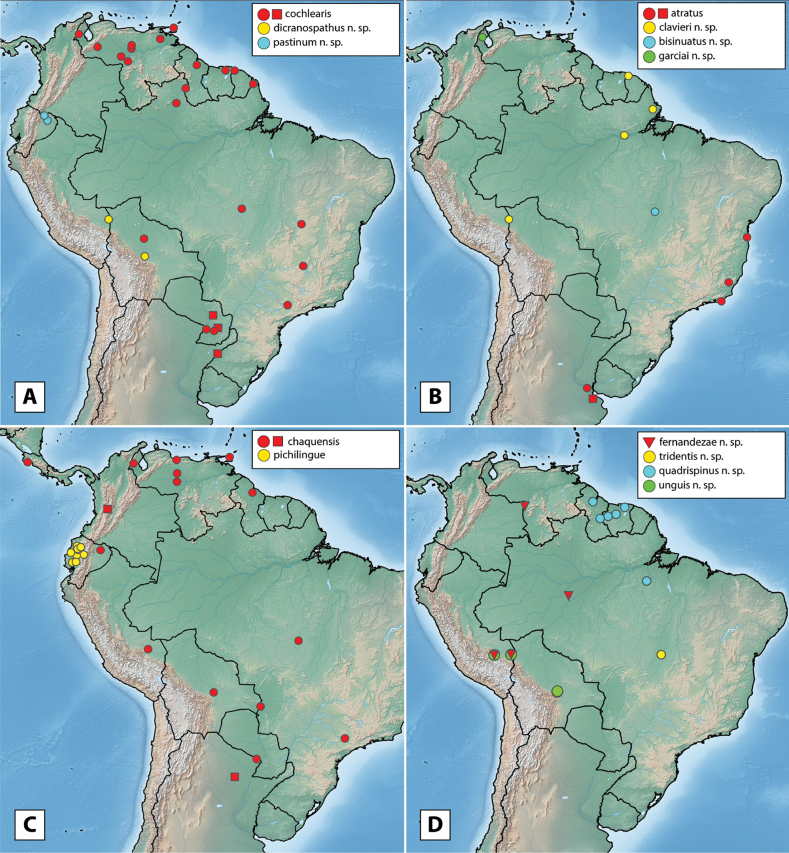
Distribution of *Novocharessallaei* species group **A***N.cochlearis* (red: circles, examined specimens; squares, literature/unconfirmed records), *N.dicranospathus* (yellow), *N.pastinum* (blue) **B***N.atratus* (red: circles, examined specimens; squares, literature/unconfirmed records), *N.clavieri* (yellow), *N.bisinuatus* (blue), *N.garciai* (green) **C***N.chaquensis* (red: circles, examined specimens; squares, literature/unconfirmed records), *N.pichilingue* (yellow) **D***N.fernandezae* (red), *N.tridentis* (yellow), *N.quadrispinus* (blue), *N.unguis* (green).

Argentina, Brazil (Bahia, Espírito Santo, Minas Gerais, Rio de Janeiro) (Fig. 22B). Colombia, Ecuador, Paraguay, and the Brazilian state of Mato Grosso do Sul are removed from the distribution, see remarks.

##### Habitat.

Little is known about the habitat of this species.

##### Remarks.

The Colombian specimens identified by Gonzalez-Rodriguez et al. (2017) as *N.atratus* are in fact *N.chaquensis*, as can be determined by their figure 24 of the aedeagus, which matches *N.chaquensis* perfectly. Colombia is thereby removed from the range of this species. Additionally, there is a historical record from Ecuador that was published by Knisch (1925). This Ecuador record was based on two specimens from “Gualaquiza” from “Dr. Festa”. No sex is indicated, though in records of some other species in the same paper, the sex is sometimes indicated, suggesting that the specimens mentioned here may have been unsexed. Because this record is likely a misidentification and so far outside the known range of this species, which is otherwise not known from northern South America or anywhere in the Andean region, we remove Ecuador from the reported range of this species.

The records from Paraguay (Rio Alto Parana) and Mato Grosso do Sul (Corumba) in Brazil derive from specimens cited by d’Orchymont (1939) under *H.parhedrus*, which is now a synonym of *N.atratus*. However, all the specimens cited from these localities were females, so there is no way to confirm they are in fact this species and so we remove them from the currently known distribution.

#### ﻿ Novochares bisinuatus sp. nov.

Taxon classificationAnimaliaColeopteraHydrophilidae

63431524-C250-5928-A596-A3872E4017FF

https://zoobank.org/383FE863-40A5-4317-8C4B-DCA2AE0242C7

Figs 18A, B , 22B

##### Type material.

***Holotype* (male)**: “BRASIL: Goiás, Sta./ Isabel, R. Araguaia,/ Isla do Bananal/ I,8–11,1961./ B. Malkin leg.” (FMNH). ***Paratype* (1 ex.): Brazil: Goiás**: Same data as holotype (1, SEMC).

##### Differential diagnosis.

The aedeagal form of this species is most similar to *N.yanomami*, but that species has a deeply forked dorsal plate of the median lobe that extends to the apex of the parameres (Fig. 18C); it is not forked and it is much shorter in *N.bisinuatus* (Fig. 18A).

##### Description.

Body length 5.5–6.3 mm. ***Coloration***: Dorsal surfaces brown to dark brown, with slightly to strongly paler (orange) margins of clypeus, head, pronotum, and elytra. ***Head***: Maxillary palps nearly 1.2× width of head, uniformly orange in color. ***Thorax***: Ground punctation on pronotum and elytra relatively dense and moderately impressed. Each elytron with eight or nine well-defined rows of serial punctures on dorsal surface, with very faint rows (one dorsal and two or three lateral) of scarce and weakly marked systematic punctures; elytral ground punctation moderately impressed. Prosternum flat. Posterior elevation of mesoventrite transverse, curved, and posteriorly concave. ***Abdomen***: Apical emargination of fifth ventrite relatively broad and deep, U-shaped. ***Aedeagus***: (Fig. 18A, B) Overall shape pear-like, nearly 2× longer than wide; lateral projection on apical region of outer margin of each paramere pointed; at closest point, dorsal inner margins of parameres separated by distance 0.2× greatest width of a paramere; dorsal plate of median lobe with neck 0.2× as broad as base; apex of dorsal plate of median lobe medially emarginate, not forming distinct arms; gonopore placed at base of dorsal plate of median lobe; ventral plate of median lobe moderately sclerotized, triangular, apically acute, apex nearly extending to apex of dorsal plate; basal piece 0.37× length of a paramere. In lateral view, aedeagus nearly straight at base, somewhat teardrop-shaped, with ventral outline of parameres 3× longer than greatest width near basal 1/3; dorsal outline nearly straight along basal 1/2.

##### Etymology.

Referring to the bisinuate apex of the dorsal plate of the median lobe.

##### Distribution.

Known only from the type locality in central Brazil (Fig. 22B).

##### Habitat.

Nothing is known about the habitat of this species.

#### ﻿ Novochares chaquensis

Taxon classificationAnimaliaColeopteraHydrophilidae

(Fernández, 1982)

2F1C4CB1-EDF9-5674-9344-E2264DA810EF

Figs 20A–E , 22C

Helochares (s. str.) chaquensis Fernández, 1982b: 87; Fernández 1989: 148 [in key].
Helochares (s. str.) carmona Short, 2005: 215; syn. nov.
Novochares carmona (Short, 2005: 215); Girón and Short 2021: 204.
Novochares chaquensis (Fernández, 1982); Girón and Short 2021: 204.

##### Type material examined.

***Helochareschaquensis* Fernández, 1982**: Holotype male from Argentina (Chaco: San Bernardo) deposited in MLP (not seen).

***Helocharescarmona* Short, 2005: *Paratypes* (2 exs.): Costa Rica: Guanacaste Province**: nr. Carmona, laguna de Cocodrilo, 16.i.2003, leg. Short, Roughley, & Porras, HG-vapor light (2, SEMC).

##### Additional material examined

**(34 exs.). Argentina**: Formosa Province: P.N. Rio Pilcomayo, 50 km NW Clorinda, 19.xii.1990, FM#90-293, marsh edge, S & J Peck, UV light (3, FMNH). **Bolivia: Santa Cruz**: 60 mi. N. Santa Cruz, Saavedre Exp. Sta., 3–5.i.1960, leg. R. Cumming (4, USNM), same data but 27–20.xii.1959 (1, USNM). **Brazil: Mato Grosso**: Jacare, Xingu National Park, xi.1965, leg. M. Alvarenga, at light (3, UNSM). **Mato Grosso do Sul**: Corumba, Paraguay River, -18.95184, -57.66642, 101 m, 25.vi.2018, leg. Hamada & team, macrophytes along river margins and in floating island mats, BR18-0625-01A (4, INPA, SEMC). **São Paulo**: Piracicaba, 12.xii.1965, leg. C.A. Triplehorn (2, USNM). **Ecuador: Napo**: Limoncocha, 15.vi.1977, leg. P.J. Spangler & D.R. Givens #129 (1, USNM). **Guyana**: Mazaruni-Potaro District, Kartabo Point, 1.i.1983, leg. W.E. Steiner (1, USNM). **Panama: Darién**: Cana ANCON station, 500 m, 7°45.323'N, 77°41.069'W, 3–9.vi.1996, leg. S. Lingafelter, blacklight (1, SEMC). **Peru: Madre de Dios**: Parque Manu, Pakitza, Cocha Salvador, 12°07'S, 70°59'W, 250 m, 21.ix.1989, leg. R.A. Faitoute, colln. #50 (2, USNM). **Trinidad And Tobago**: Trinidad, Piarco, 15–16.vii.1969, leg. P. & P. Spangler (4, USNM). **Venezuela: Apure**: 5 km N. San Juan de Payara, 350’, 25.vii.1988, leg. C. & L. O’Brien & G. Wibmer (2, CAS). **Aragua**: El Limon, 450 m, 25.v.1977, leg. J. Clavijo, at light (3, MIZA). **Guárico**: Corozo Pando (8 km. N.), 17–18.vi.1984, leg. F.W. Eiland & V. Linares, blacklight (2, USNM). **Zulia**: 9°51.833'N, 72°43.285'W, 96 m, btw Machiques & Tukuko, 29.i.2009, leg. Short, García, & Camacho, roadside marsh, VZ09-0129-03Z (1, SEMC).

##### Differential diagnosis.

This widespread species has several less common aedeagal features, particularly the straight, untoothed apices of the parameres (Fig. 20A). The shallow, generally smooth fork at the apex of the dorsal plate of the median lobe is somewhat similar to *N.garciai*, but that species has strongly sinuate outer margins of the parameres, as well as the dorsal plate being constricted medially (Fig. 20F).

##### Description.

Body length 5.2–7.0 mm. ***Coloration***: Dorsal surfaces brown to dark brown, usually with slightly paler (brown to orange) margins of pronotum, and elytra. ***Head***: Maxillary palps 1.2–1.3× width of head, orange to brown in color, with apex of each palpomere paler. ***Thorax***: Ground punctation on pronotum and elytra relatively dense and shallowly impressed. Elytra without rows of serial punctures, each with very faint rows (one dorsal and two or three lateral) of scarce and weakly marked systematic punctures. Prosternum medially broadly convex. Posterior elevation of mesoventrite broadly and roundly elevated, with medial longitudinal ridge extending anteriorly. ***Abdomen***: Apical emargination of fifth ventrite relatively deep, U- to V-shaped. ***Aedeagus***: (Fig. 20A–E) Overall shape pear-like, 4.5× longer than wide, with outer lateral margins of parameres straight to weakly and evenly convex along basal 3/4; apical region of each paramere rounded, with outer margin smooth, not pointed; at closest point, dorsal inner margins of parameres separated by distance 0.5–1.0× greatest width of a paramere; dorsal plate of median lobe with neck 0.35–0.50× as broad as base; arms of dorsal plate of median lobe diverging, dorsally concave, nearly 0.1× length of dorsal plate of median lobe; each arm uniformly wide along basal 2/3, then narrowing to rounded to acute apex; notch between arms at base slightly wider than base of an arm; gonopore placed at base of dorsal plate of median lobe; ventral plate of median lobe weakly sclerotized, nearly membranous, triangular, irregular at apex, apex extending to second 1/3 of neck of dorsal plate; basal piece 0.3× length of a paramere. In lateral view, aedeagus oblique at base, with ventral outline of parameres 4.2× longer than greatest width near base; dorsal outline of aedeagus in lateral view straight nearly along basal 2/3.

##### Distribution.

Argentina, Bolivia (new record), Brazil (Mato Grosso, Mato Grosso do Sul, São Paulo), Colombia (new record), Costa Rica (new record), Ecuador (new record), Guyana (new record), Panama (new record), Peru (new record), Trinidad and Tobago (new record), Venezuela (new record) (Fig. 22C). The only previously published locality that we have not examined specimens or images is the type locality in Argentina.

##### Habitat.

Little is known about this species: most specimens were taken at light traps. One series of specimens was collected in floating macrophytes on the Paraguay River, others were collected in marshes.

##### Remarks.

This species occurs from Costa Rica south to Argentina. Despite this vast range, it is rather uncommonly collected, with just a smattering of records known to us. Specimens from Colombia identified by Gonzalez-Rodriguez et al. (2017) as *N.atratus* are in fact this species.

We examined the aedeagus of a paratype of the hitherto Costa Rican endemic *N.carmona*, which we found to be an exact match to *N.chaquensis* leading us to synonymize the two species.

#### ﻿ Novochares clavieri sp. nov.

Taxon classificationAnimaliaColeopteraHydrophilidae

870B8382-4A27-5AAD-8019-FFA4C0D69CC1

https://zoobank.org/CEC6D3C6-CA04-44D7-BDA6-7A714941A7C5

Figs 18L , 22B

##### Type material.

***Holotype* (male): Brazil**: Pará: Alenquer/ -1.96253, -54.50458; 44m/ ca. 25 km E of Alenquer;/ Palm swamp, lots of detritus/ 4.ii.2018; Short & Benetti;/ BR18-0204-01A (INPA). ***Paratypes* (11 exs.): Brazil: Amapá**: Tartarugalzinho (22 km S) on BR-156, 1.30747, -50.93803, 41 m, 23.vii.2018, leg. Short, marsh/pond, BR18-0723-02A (1, SEMC, DNA voucher SLE2085). **Pará**: Same data as holotype (8, INPA, SCC, SEMC, including DNA voucher SLE1514). **French Guiana**: St. Laurent du Maroni, Sentier des Malgaches, 5.48627, -54.00238, 14 m, 4.iii.2020, leg. Short & Neff, pond in secondary forest, FG20-0304-01A (1, SEMC, DNA voucher SLE2420). **Peru: Madre de Dios**: Tambopata, Kawsay Biological Station, -12.54034 S, -69.00074W, 190 m, 4.vi.2022, leg. Short et al., palm swamp with lots of detritus (1, MHNSM).

##### Differential diagnosis.

This species, with its moderately sinuate outer paramere margins and Y-shaped dorsal plate of the median lobe (Fig. 18L), is at first glance most similar to *N.garciai* (Fig. 20F), and also to *N.fernandezae* (Fig. 18F) and *N.unguis* (Fig. 18J). However, these species all differ in the specific shapes of the median lobe and parameres.

##### Description.

Body length 5.2–5.6 mm. ***Coloration***: Dorsal surfaces brown to dark brown, with slightly paler (brown to orange) clypeus and margins of pronotum, and elytra. ***Head***: Maxillary palps nearly 1.7× width of head, orange in color, with apex of each palpomere paler. ***Thorax***: Ground punctation on pronotum and elytra relatively dense and very shallowly impressed. Elytra without rows of serial punctures, each with very faint rows (one dorsal and two or three lateral) of scarce and weakly marked systematic punctures. Prosternum medially weakly convex. Posterior elevation of mesoventrite broadly and roundly elevated, with low medial longitudinal ridge extending anteriorly. ***Abdomen***: Apical emargination of fifth ventrite relatively deep and broad, U-shaped. ***Aedeagus***: (Fig. 18L) Overall shape pear-like, nearly 2× longer than wide, with outer lateral margins of parameres evenly sinuate; lateral projection on apical region of outer margin of each paramere pointed; at closest point, dorsal inner margins of parameres separated by distance 0.23× greatest width of a paramere; dorsal plate of median lobe with neck 0.2× as broad as base; arms of dorsal plate of median lobe diverging, nearly 0.35× length of dorsal plate of median lobe; gonopore placed near base of dorsal plate of median lobe; ventral plate of median lobe not visible; basal piece 0.35× length of a paramere. In lateral view, aedeagus nearly straight at base, somewhat triangular, with ventral outline of parameres 2× longer than greatest width near basal 1/3; dorsal outline nearly straight.

##### Etymology.

Named in honor of Simon Clavier, Aquatic Biologist in French Guiana, who has contributed to our knowledge of the aquatic beetle fauna of the region and who assisted AEZS with fieldwork.

##### Distribution.

Brazil (Amapá, Pará), French Guiana, Peru (Fig. 22B).

##### Habitat.

This species has been collected from ponds and swamps with abundant detritus.

#### ﻿ Novochares cochlearis

Taxon classificationAnimaliaColeopteraHydrophilidae

(Fernández, 1982)

F7E19A97-E332-53B9-B682-8253B0C2D4D1

Figs 6B , 17C , 18M–P , 22A

Helochares (s. str.) cochlearis Fernández, 1982b: 89; Fernández 1989: 148 [in key].
Novochares cochlearis (Fernández, 1982); Girón and Short 2021: 204.

##### Type material.

***Holotype*** male from Argentina (Corrientes, Santo Tomé) deposited in MACN (not seen).

##### Material examined

**(83 exs.). Bolivia: Beni Department**, Cercado Province, 9.5 km N. of Trinidad, 17.vi.1999, leg. K.B. Miller (1, SEMC). **Brazil: Amapá**: Oiaqpoque (ca. 5.5 km NE), balneario, 18.vii.2018; leg. Short; seepage area, BR18-0718-01B (DNA Voucher SLE1922). **Bahia**: Roda Velha, ca. 25 km S on BR-20, -12.97821, -45.99091, 805 m, 22.ii.2018, leg. Benetti & Team, marginal marsh of river, BR18-0222-03A (16, SEMC, INPA, including DNA Voucher SLE1628). **Mato Grosso**: Jacare, Zingu National Park, xi.1965, M. Alvarenga, at light (1, USNM); Luizlândia do Oeste, 18 km W on BR-40, -17.99086, -45.78403, 768 m, 3.iii.2018, leg. Benetti & team, marsh area in valley next to stream, BR18-0303-01A (DNA Voucher SLE2080). **Roraima**: BR-401, ca. 6 km SW of Bonfim, 3°21.615'N, 59°53.361'W, 100 m, 12.i.2018, leg. Short, Benetti & Santana, large marsh with abundant vegetation, BR18-0112-02A (3, SEMC); Caracaraí, ca. 30 km SE, on BR-174, 1°35.091'N, 61°00.118'W, 80 m, 16.i.2018, leg. Short, Benetti, & Santana, marsh, BR18-0116-05A (1, SEMC). **São Paulo**: Piracicaba, 12.xii.1965, leg. C.A Triplehorn (3, UNSM), same data except 6.x.1965, blacklight trap (1, USNM); same data except 15.i.1966 (1, USNM). **French Guiana**: St. Laurent du Maroni, Sentier des Malgaches, 5.48627, -54.00238, 14 m, 4.iii.2020, leg. Short & Neff, pond in secondary forest, FG20-0304-01A (6, SCC, SEMC); “Guyane”, Mission Balachowsky-Gruner, Foret d’Acarouany, 19.x.1969 (1, USNM) [note: this male specimen was labeled by P. Spangler as being compared with the type of “*Helocharesguianus* BB”. **Guyana**: “Hope LT.”, 16–20.vii.1962, leg. J. Maldonado C. (2, USNM); “Essequibo R.”, Moraballi Creek, 19.x.1929, Oxford University Expedition, “clearing” (1, USNM). **Paraguay**: 3.9 km South Villarrica, 2.xii.1973, leg. O. Flint Jr. (8, USNM); Paraguarí Department, Arroyo Caanabe, 12.iv.1980, leg. P.J. Spangler. **Suriname: Pará**: Along Martin Luther King Highway, SR12-0723-02A (1, SEMC); SR12-0306-01A (4, SEMC; TTU-Z). **Trinidad And Tobago**: Trinidad, Piarco, 15–16.vii.1969, leg. P. & P. Spangler (3, USNM). **Venezuela: Apure**: road between San Fernando and Rio Capanaparo, 0.5 km N. Rio Claro, 7°10.162'N, 67°38.69'W, 50 m, 4.i.2006, leg. Short & Torres, roadside ditch/swale, AS-06-009 (3, SEMC, TTU-Z); ca. 6 km S. Rio Cinaruco, Road between Rio Orinoco & Rio Cinaruco, 8.i.2006, morichal and marsh along road, leg. Short, AS-06-019 (2, SEMC). **Barinas**: Ciudad Bolivia, approx. 13 km SE, large Hacienda, 8°19.394'N, 70°28.238'W, 173 m, 21.i.2012, leg. Short, Arias, & Gustafson, marsh, VZ12-0125-02A (1, SEMC). **Bolívar**: Los Pijiguaos, outcrop/ morichal, 6°35.617'N, 66°49.238'W, 60–80 m, 12.i.2009, leg. Short, Camacho, García, Joly, & Miller, algae on rocky margin of morichal, VZ09-0112-01B (1, SEMC). **Guárico**: San Nicolasito Field Station, 8°8.296'N, 66°24.459'W, 10.i.2009, leg. Short & Miller, VZ09-0110-02X (16, SEMC, including DNA voucher SLE1196); Las Mercedes, approx. 65 km S, 8°31.705'N, 66°22.602'W, 145 m, 9.i.2009, leg. Short, García, Camacho, & Miller, large vegetated lagoon, VZ09-0109-01X (5, SEMC). **Monagas**: S of Maturin, morichal at road crossing, 9°16.398'N, 62°56.246'W, 22 m, 2.ii.2010, leg. Short, García, & Joly, morichal margin, VZ10-0202-02A (6, MIZA, SEMC, including DNA voucher SLE1175). **Zulia**: between Machiques and Tukuko, 9°51.883'N, 72°43.285'W, 96 m, 29.i.2009, leg. Short, Camacho, & García, roadside marsh, VZ09-0129-03X (1, SEMC); El Tucuco, 420 m, 12–27.v.1971, leg. C.J. Rosales, J. Salcedo, A. Ramirez (2, MIZA).

##### Differential diagnosis.

The form of the aedeagus of this species is extremely distinctive, with both the broadly triangular and undulating parameres and the spoon-shaped dorsal plate of the median lobe (Fig. 18M–P). There is no other species that even closely resembles this aedeagal form.

##### Description.

Body length 5.0–6.5 mm. ***Coloration***: Dorsal surfaces brown to dark brown, with slightly paler (brown to orange) margins of clypeus, pronotum, and elytra. ***Head***: Maxillary palps 1.4–1.6× width of head, uniformly orange to brown in color (Fig. 17C). ***Thorax***: Ground punctation on pronotum and elytra relatively dense and very shallowly impressed. Elytra without rows of serial punctures, each with very faint rows (one dorsal and two or three lateral) of scarce and weakly marked systematic punctures. Prosternum flat to medially very weakly convex. Posterior elevation of mesoventrite weakly, broadly, and roundly elevated, with low medial longitudinal ridge extending anteriorly. ***Abdomen***: Apical emargination of fifth ventrite relatively deep and broad, U-shaped. ***Aedeagus***: (Fig. 18M–P) Overall shape somewhat rhomboid, 1.7× longer than greatest width, with outer lateral margins of parameres irregularly convex; apical region of each paramere with outer margin smooth, not pointed; at closest point, dorsal inner margins of parameres separated by distance 0.7× greatest width of a paramere; dorsal plate of median lobe with narrowest point of neck near base of plate, 0.15× as broad as base, gradually widening towards apical region; apical region of dorsal plate of median lobe dorsally concave, variable in length and shape, from oval to rhomboidal; gonopore placed at base of dorsal plate of median lobe; ventral plate of median lobe weakly sclerotized, not visible; basal piece strongly reduced. In lateral view, aedeagus strongly oblique at base, with ventral outline of parameres 3.4× longer than greatest width near base; dorsal outline of aedeagus in lateral view somewhat concave along basal 1/2.

##### Distribution.

Argentina, Bolivia (new record), Brazil (new record; Amapá, Bahia, Minas Gerais, Roraima, São Paulo), French Guiana (new record), Guyana (new record), Paraguay, Suriname (new record), Trinidad and Tobago (new record), Venezuela (new record) (Fig. 22A).

##### Habitat.

This species has generally been collected in lentic habitats such as marshes, pond margins, and roadside ditches.

##### Remarks.

This species has a very distinct aedeagus, with the flattened and ear-like shape of the median lobe unlike any others we have seen. The species is widely distributed in South America, and there is some variation in the precise shape of the dorsal plate of the median lobe, ranging from more circular (Fig. 18M) to more elongate (Fig. 18P) as well as the width and sinuosity of the outer margins of the parameres. There is also a higher (but not extreme) level of genetic divergence in COI between the northern and southern populations we sampled (maximum intraspecific difference of 5.1%; Fig. 1). It is possible that further detailed study of this species will reveal it to be a species complex.

The apical region of the dorsal plate of the median lobe in Fig. 18O is broken along the midline, which does not represent intraspecific variation.

There is a dissected male specimen from Guyana of this species in the USNM that is labeled “Helocharesguianus JBB” [J. Balfour-Browne]. There is no record of this name ever having been published in the literature.

#### ﻿ Novochares dicranospathus sp. nov.

Taxon classificationAnimaliaColeopteraHydrophilidae

4F051EE6-1234-57C5-B7B4-FC5301FD8199

https://zoobank.org/34766F63-9CE4-471C-B58B-9AA3E539D5EE

Figs 20G–I , 22A

##### Type material.

***Holotype* (male)**: “PERU: Tambopata Prov./ 15 km NE Pto. Maldonado/ 30 June 1989, 200 m/ J. Ashe, R. Leschen, #336/ ex. at light” (SEMC). ***Paratypes* (1 ex.): Bolivia: Cochabamba**: Puerto Villarroel env., “6.12.2001”, leg. O. Safranek (1, SEMC).

##### Differential diagnosis.

The aedeagus of this species is rather distinct, with the expanded and bifid, spoon-like shape of the apex of the dorsal plate of the median lobe (Fig. 20G–I). The strong dorso-ventrally sinuate form of the dorsal plate of the median lobe (seen easily in lateral view, Fig. 20I) is most similar overall to *N.chaquensis* (Fig. 20C) and *N.garciai*.

##### Description.

Body length 5.6–6.0 mm. ***Coloration***: Dorsal surfaces brown to dark brown, with slightly paler (brown to orange) margins of pronotum, and elytra. ***Head***: Maxillary palps 1.2–1.4× width of head, uniformly orange to brown in color. ***Thorax***: Ground punctation on pronotum and elytra relatively dense and very shallowly impressed. Elytra without rows of serial punctures, each with very faint rows (one dorsal and two or three lateral) of scarce and weakly marked systematic punctures. Prosternum medially weakly convex. Posterior elevation of mesoventrite weakly, broadly, and somewhat longitudinally elevated, with low medial longitudinal ridge extending anteriorly. ***Abdomen***: Apical emargination of fifth ventrite relatively deep and broad, U-shaped. ***Aedeagus***: (Fig. 20G–I) Overall shape elongated-oval, 2.8× longer than greatest width, with outer lateral margins of parameres nearly evenly convex up to apical region; apical region of each paramere rounded, with outer margin smooth, not pointed; at closest point, dorsal inner margins of parameres separated by distance slightly wider than greatest width of a paramere; dorsal plate of median lobe with neck 0.2× as broad as base; dorsal plate of median lobe dorsally concave along apical 1/3, with arms nearly 0.1× length of dorsal plate of median lobe; each arm nearly uniformly wide along entire length, apically obliquely truncate (outer margin shorter); notch between arms at base nearly 0.3× width of an arm at base; gonopore placed at base of dorsal plate of median lobe; ventral plate of median lobe not visible; basal piece 0.27× length of a paramere. In lateral view, aedeagus oblique at base, with ventral outline of parameres 3× longer than greatest width near base; dorsal outline of aedeagus in lateral view somewhat concave along basal 1/3.

##### Etymology.

A combination of the Greek *dikranon* (pitchfork) and *spathe* (paddle for stirring); referring to the spoon-like shape of the dorsal plate of the median lobe that bifurcates at apex.

##### Distribution.

Known from a pair of localities in Peru and Bolivia (Fig. 22A).

##### Habitat.

Nothing is known about the habitat of this species.

#### ﻿ Novochares fernandezae sp. nov.

Taxon classificationAnimaliaColeopteraHydrophilidae

6C4F617E-7E1E-5F44-A465-F9DF1A7E62C9

https://zoobank.org/9E9FF77D-317E-4073-BBD2-30D1EA9DFF9D

Figs 18F–I , 22D

##### Type material.

***Holotype* (male)**: “VENEZUELA: T.F. Amaz./ Puerto Ayacucho/ 22 January 1985/ G. E. Ball, collr.”, “in small ponds full/ of dead leaves;” (USNM). ***Paratypes* (12 exs.): Brazil: Amazonas**: Tapauá, Humaita (ca. 240 km N) on BR-319, -5.50298, -62.12392, 54 m, 11.vii.2018, leg. Short, forest detrital pool, BR18-0712-01B (1, INPA, DNA voucher SLE1992). **Peru: Cusco**: Pilcopata, 600 m, 8–10.xii.1979, premontane moist forest, leg. J.B. Heppner (1, USNM). **Madre de Dios**: Rio Tambopata Reserve, ca. 30 km SW Puerto Maldonado, 290 m, 16–20.xi.1979, leg. J.B. Heppner, subtropical humid forest (1, USNM); Tambopata (1, SEMC, DNA voucher SLE1099). **Venezuela: Amazonas**: Same data as holotype (8, USNM, SEMC).

##### Differential diagnosis.

The aedeagus of this species (Fig. 18F) somewhat resembles several others, including *N.unguis* (which has much wider and thicker parameres and a shallower fork in the apex of the median lobe; Fig. 18J) and *N.clavieri* (which has more sinuate parameres and different median lobe shape; Fig. 18L).

##### Description.

Body length 5.2–5.9 mm. ***Coloration***: Dorsal surfaces brown to dark brown, with slightly paler (brown to orange) margins of clypeus, pronotum, and elytra. ***Head***: Maxillary palps 1.6–1.7× width of head, uniformly orange to brown in color. ***Thorax***: Ground punctation on pronotum and elytra relatively dense and very shallowly impressed. Elytra without rows of serial punctures, each with very faint rows (one dorsal and two or three lateral) of scarce and weakly marked systematic punctures. Prosternum medially weakly convex. Posterior elevation of mesoventrite weakly, broadly, and somewhat longitudinally elevated, with low medial longitudinal ridge extending anteriorly. ***Abdomen***: Apical emargination of fifth ventrite relatively deep and broad, U-shaped. ***Aedeagus***: (Fig. 18F–I) Overall shape pear-like, 2.5× longer than wide, with outer lateral margins of parameres nearly evenly convex up to apical region; apical region of each paramere rounded, partly membranous, with outer margin very weakly pointed; at closest point, dorsal inner margins of parameres separated by distance equal to greatest width of a paramere; dorsal plate of median lobe with neck 0.3× as broad as base; each lateral margin of dorsal plate of median lobe with long and narrow projection emerging near base of neck; neck dorsally concave; arms of dorsal plate of median lobe diverging, nearly 0.3× length of dorsal plate of median lobe; each arm apically and gradually narrowing to acute apex; notch between arms at base nearly as wide as base of an arm; gonopore placed near base of dorsal plate of median lobe; ventral plate of median lobe not visible; basal piece 0.38× length of a paramere. In lateral view, aedeagus oblique at base, with ventral outline of parameres 3.1× longer than greatest width near base; dorsal outline of aedeagus in lateral view slightly concave along basal 1/3, then nearly straight to apex; ventral outline of aedeagus in lateral view convex along basal 1/3.

##### Etymology.

In honor of aquatic beetle specialist Dr. Liliana Fernández, who significantly expanded our knowledge of this genus and other groups of Hydrophilidae in South America.

##### Distribution.

Brazil (Amazonas), Peru, Venezuela (Fig. 22D).

##### Habitat.

This species has been collected in forested detrital pools.

#### ﻿ Novochares garciai sp. nov.

Taxon classificationAnimaliaColeopteraHydrophilidae

452372F1-3AC1-5577-A2A8-211520A33109

https://zoobank.org/F99A4F52-3A1B-44C1-8C8B-EFEA781D31B9

Figs 20F , 22B

##### Type material.

***Holotype* (male)**: “Venezuela-Zulia/ Mision El Rosario/ 50m, 12-13-I-1977”, “L.J. Joly T./ J. Salcedo/ J. Clavijo” (MIZA).

##### Differential diagnosis.

See differential diagnosis of *N.clavieri*.

##### Description.

Body length 5.8–6.0 mm. ***Coloration***: Dorsal surfaces dark brown, with very slightly paler (brown) margins of pronotum and elytra. ***Head***: Maxillary palps nearly 1.3× width of head, uniformly brown in color. ***Thorax***: Ground punctation on pronotum and elytra relatively dense and very shallowly impressed. Elytra without rows of serial punctures, each with very faint rows (one dorsal and two or three lateral) of scarce and weakly marked systematic punctures. Prosternum medially very weakly convex. Posterior elevation of mesoventrite weakly, broadly, and somewhat longitudinally elevated, with low medial longitudinal ridge extending anteriorly. ***Abdomen***: Apical emargination of fifth ventrite relatively deep and broad, U-shaped. ***Aedeagus***: (Fig. 20F) Overall shape pear-like, 2.5× longer than wide, with outer lateral margins of parameres evenly convex along basal 4/5; apical region of each paramere rounded, with outer margin smooth, not pointed; at closest point, dorsal inner margins of parameres separated by distance 0.4× greatest width of a paramere; dorsal plate of median lobe with neck dorsally concave, 0.35× as broad as base; arms of dorsal plate of median lobe diverging, nearly 0.18× length of dorsal plate of median lobe; each arm gradually and uniformly narrowing to rounded apex; notch between arms at base slightly wider than base of an arm; gonopore placed at base of neck of dorsal plate of median lobe; ventral plate of median lobe not clearly visible; basal piece 0.4× length of a paramere. In lateral view, aedeagus oblique at base, with ventral outline of parameres 3.7× longer than greatest width near base; dorsal outline of aedeagus in lateral view straight nearly along basal 2/3.

##### Etymology.

In honor of Venezuelan aquatic researcher Mauricio García, in recognition of his contributions to the knowledge of Venezuelan aquatic beetles.

##### Distribution.

Known only from the type locality (Fig. 22B).

##### Habitat.

Nothing is known about the habitat of this species.

#### ﻿ Novochares guadelupensis

Taxon classificationAnimaliaColeopteraHydrophilidae

(d’Orchymont, 1926)

18A9DFCA-BB17-55E3-A966-0E602CC254A0

**Figure 23.**
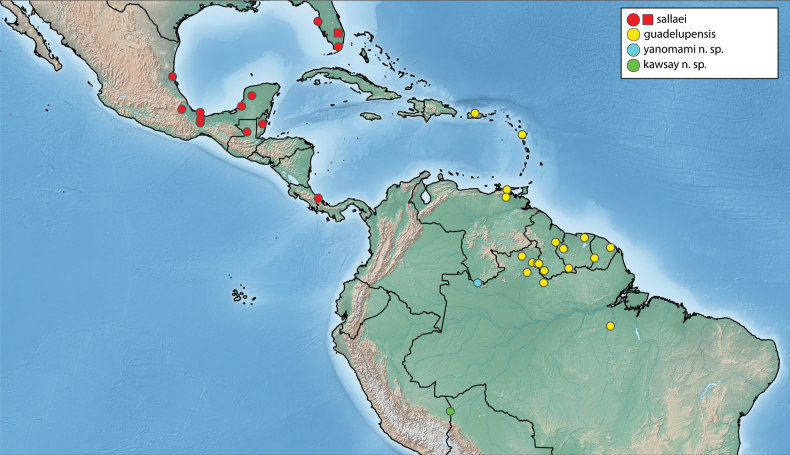
Distribution of *Novocharessallaei* species group. *N.sallaei* (red: circles, examined specimens; squares, literature/unconfirmed records), *N.guadelupensis* (yellow), *N.yanomami* (blue), *N.kawsay* (green).

Figs 17A , 19A–H , 23

Helochares (s. str.) guadelupensis d’Orchymont, 1926: 233.
Novochares guadelupensis (d’Orchymont, 1926); Girón and Short 2021: 204.

##### Material examined

**(155 exs.). Brazil: Pará**: Rio Xingu Camp, ca. 60 km S. Altamira, Igarape Jabuti, 8-16.x.1986, leg. P. Spangler & O. Flint, malaise trap (1, USNM); Rio Xingu Camp, ca. 60 m S. Altamira, 8.x.1986, leg. P. Spangler & O. Flint, first jungle stream on trail 4, Colln. #14 (4, USNM); same data but 10.x.1986, Colln. #19 (4, USNM). **Roraima**: BR-401, ca. 26 km NE of Boa Vista, 2°56.191'N, 60°28.017'W, 92 m, 12.i.2018, leg. Short, pooled up morichal, BR18-0112-06B (29, INPA, SEMC); Amajari, ca. 16 km W on RR-203, 3°36.874'N, 61°33.470'W, 125 m, leg. Short, Benetti & Santana, marsh, BR18-0113-04A (3, SEMC); Sitio Bem Querer, ca. 2 km W, along road, 1°55.737'N, 61°01.372'W, 116 m, leg. Short, Benetti, & Santana, forested detrital pool, BR18-0116-03A (1, SEMC); Caroebe, Rio Jatapu, nr. Usina de Jatapu, 00°50.939'N, 59°18.262'W, 145 m, 17.i.2018, leg. A. Short, marginal pools of river, BR18-0117-02A (9, SEMC). **French Guiana**: Anapaike Village, Lawa River, 22-25.ix1963, leg. B. Malkin (26, USNM); Auberge des Orpailleurs, Marsh on RN2, 4.51138, -52.35079, 14 m, 11.iii.2020, leg. Short & Neff, shallow marsh, FG20-0311-02A (1, SEMC). **Guadeloupe**: Pointe-a-Pietra, 1936, leg. H. Stehle (3, USNM). **Guyana: Region 9**: Tributary of the Takatu River, NW of Kusad Mts., 2°50.563'N, 59°59.113'W, 109 m, 24.x.2013, leg. Short, Isaacs, & Salisbury, vegetated creek margins, GY13-1024-02B (8, SEMC, including DNA voucher SLE1200); Ziida Karisihizi (Lake), nr. Kusad Mts., 2°49.793'N, 59°48.361'W, 123 m, 25.x.2013, leg. Short, Isaacs, & Salisbury, large vegetated marsh, GY13-1025-01A (2, SEMC). Parabara, trail to mines, 2°05.095'N, 59°14.174'W, 250 m, 2.xi.2013, leg. Short, Isaacs and Salisbury, detrital pools in forest, GY13-1102-01A (1, SEMC); Parabara north side of river, 2°06.492'N, 59°13.653'W, 274 m, 3.xi.2013, detritus margins and leaf packs, GY13-1103-02A (1, SEMC); Parabara, at N. edge of village, 2°05.733'N, 59°14.390'W, 248 m, leg. Short, Isaacs and Salisbury, small vegetated marsh, GY13-1103-03A (14, CBDG, SEMC, TTU-Z). **Puerto Rico**: L[aguna] Tortuguero, 2.viii.1962, leg. O. & R. Flint, Matthews (4, USNM, including homotype designated by P. Spangler). **Suriname: Para**: nr. Overbridge River Resort, 5°31.8'N, 55°3.5'W, 14–15.ii.2010, leg. P. Skelley, W. Warner, & C. Gillet (2, SEMC). **Sipaliwini**: Iwaana Saamu, forest swamp, 26.viii.2010, leg. Short, SR10-0826-01A (1, SEMC); Camp 3, Werehpai, 2°21.776'N, 56°41.861'W, 237 m, 3–7.ix.2010, leg. Short and Kadosoe, pooled up detrital creek, SR10-0903-01A (4, SEMC); same data except detrital forest pools, SR10-0903-02A (4, SEMC); same data except 5.ix.2010, small stream, SR10-0905-01A (1, SEMC); Sipaliwini Savanna Nature Reserve, Sipaliwini Village, 2°01.194'N, 56°07.433'W, 270 m, 29.iii.2017, leg. Short; flooded forest along path to ACT, SR17-0329-01A (1, SEMC); Kabalebo Nature Resort, Moi Moi Creek, 4.42313°N, 57.19198°W, 104 m, 10–14.iii.2019, leg. Short, detrital pool, SR19-0310-01G (19, NZCS, SCC, SEMC, TTU-Z, including DNA voucher SLE1803); Kabalebo Nature Resort: Sand Creek, 4.38476°N, 57.24636°W, 72 m, 13-15.iii.2019, leg. Short & Baca, large isolated pool near creek SR19-0313-01B (5, SEMC, including DNA voucher SLE2117). Suriname District: Krakka-Phedra Rd., 25.x.1962, leg. B. Malkin (1, USNM). **Venezuela: Monagas**: La Esperanza, 10.vi.1967, leg. J. Salcedo & L. Rodriguez (2, MIZA); **Sucre**: El Pilar, approx. 5 km SE, 10°31.419'N, 63°7.070'W, 2 m, 29.i.2010, leg. Short & García, marsh/swamp along road, VZ10-0129-04A (4, MIZA, SEMC).

##### Differential diagnosis.

This is one of only two species in which the dorsal plate of the median lobe extends beyond the apices of the parameres (Fig. 19A). The only species it could be confused with is *N.kawsay* (Fig. 19I) which is extremely similar except for the distinct apical notch and subapical constriction of the dorsal plate of the median lobe.

##### Description.

Body length 4.9–6.6 mm. ***Coloration***: Dorsal surfaces brown to dark brown, with slightly paler (brown to yellow) margins of clypeus, pronotum and elytra. ***Head***: Maxillary palps 1.4–1.6× width of head, uniformly brown in color (Fig. 17A). ***Thorax***: Ground punctation on pronotum and elytra relatively dense and shallowly impressed. Elytra without rows of serial punctures, each with very faint rows (one dorsal and two or three lateral) of scarce and weakly marked systematic punctures. Prosternum flat to medially very weakly convex. Posterior elevation of mesoventrite weakly, broadly, and somewhat longitudinally elevated, with low medial longitudinal ridge extending anteriorly. ***Abdomen***: Apical emargination of fifth ventrite relatively deep and broad, U-shaped. ***Aedeagus***: (Fig. 19A–H) Overall shape sub-rectangular, nearly 1.4–1.8× longer than wide, with outer lateral margins of parameres weakly convex and very weakly sinuate near mid-length; apical region of each paramere rounded and partly membranous, with outer margin pointed; at closest point, dorsal inner margins of parameres separated by distance 0.25× greatest width of a paramere; dorsal plate of median lobe extending beyond apex of parameres, spatula-like, with narrowest point of neck near mid-length of plate, 0.2× as broad as base, gradually widening towards third 1/4, then nearly parallel-sided to apex; arms of dorsal plate of median lobe very short, sometimes only indicated by a medial emargination at apex of plate; each arm rounded at apex; gonopore placed at base of dorsal plate of median lobe; ventral plate of median lobe not visible; basal piece 0.3× length of a paramere. In lateral view, aedeagus triangular, straight at base, with ventral outline of parameres 1.8× longer than greatest width near base; dorsal outline of aedeagus in lateral view obliquely straight nearly along basal 5/6.

##### Distribution.

Brazil (new record; Pará, Roraima), French Guiana (new record), Guadeloupe, Guyana (new record), Peru (new record), Puerto Rico (new record), Suriname (new record), Venezuela (new record) (Fig. 23).

##### Habitat.

This species has been collected from a broad range of habitats, including open ponds and marshes as well as forested pools and creek margins.

##### Remarks.

After this species was described 96 years ago by d’Orchymont, it was never discussed or reported again, nor has its aedeagus been previously illustrated. Although described from the relatively small island of Guadeloupe in the Lesser Antilles, it is actually fairly widespread in northern South America. The original description by d’Orchymont (1926) mentions the median lobe is quadrate and spatulate, which matches the form of the specimens we have seen, including those from Guadeloupe. In addition, we examined a homotype of this species designated by Paul Spangler from Puerto Rico that matches our concept here, although we did not examine the type material. The apex of the dorsal plate of the median lobe in Fig. 19D is longitudinally broken at apex, which does not represent observed intraspecific variation.

#### ﻿ Novochares kawsay sp. nov.

Taxon classificationAnimaliaColeopteraHydrophilidae

62698326-F7D8-58F4-BA79-EA7B3C49CCFB

https://zoobank.org/7AEC851B-747E-47F7-9E7A-C547D0C5AE6F

Figs 19I–L , 23

##### Type material.

***Holotype* (male)**: “PERU: Madre de Dios: Tambopata/ -12.53550°S, -69.01205°W, 190m/ Kawsay Biological Station, 3.vi.2022/ Large detrital pool nr. banana area/ PE22-0603-02B, leg. Short et al.” (MHNSM). ***Paratypes* (5 exs.): Peru: Madre de Dios**: Same data as holotype (3, MHNSM, SEMC); same locality as holotype but 2.vi.2022, swamp pool in forest, PE22-0602-01C (1, SEMC); same data except swamp pool with detritus and mud, PE22-0602-01A (1, SEMC).

##### Additional material examined

**(1 ex.). Ecuador: Napo**: Limoncocha, 15.vi.1977, leg. P.J. Spangler & D.R. Givens #129 (1, USNM).

##### Differential diagnosis.

See differential diagnosis for *N.guadelupensis*.

##### Description.

Body length 5.3–6.1 mm. ***Coloration***: Dorsal surfaces brown to dark brown, with very slightly paler (brown to yellowish) margins of clypeus, pronotum and elytra. ***Head***: Maxillary palps nearly 1.5× width of head, uniformly brown or orange in color. ***Thorax***: Ground punctation on pronotum and elytra relatively dense and shallowly impressed. Elytra without rows of serial punctures, each with very faint rows (one dorsal and two or three lateral) of scarce and weakly marked systematic punctures. Prosternum medially very weakly convex. Posterior elevation of mesoventrite weakly, broadly, and somewhat longitudinally elevated, with low medial longitudinal ridge extending anteriorly. ***Abdomen***: Apical emargination of fifth ventrite relatively deep and broad, U-shaped. ***Aedeagus***: (Fig. 19I–L) Overall shape sub-rectangular, nearly 1.3× longer than wide, with outer lateral margins of parameres nearly straight, very weakly sinuate along mid-length; apical region of each paramere rounded and partly membranous, with outer margin pointed; at closest point, dorsal inner margins of parameres separated by very narrow distance; dorsal plate of median lobe extending to or beyond apex of parameres, with neck slightly to strongly constricted at base of fork, dorsally concave, and continuing as short fork; arms of dorsal plate of median lobe slightly diverging along inner margins, nearly 0.1× length of dorsal plate of median lobe; each arm narrowing towards apex, apically rounded; notch between arms at base nearly 0.3× or as wide as width of an arm at base; gonopore placed at base of dorsal plate of median lobe; ventral plate of median lobe strongly sclerotized, triangular, narrow, rounded at apex, apex extending to before constriction of neck of dorsal plate; basal piece 0.55× length of a paramere. In lateral view, aedeagus triangular, straight at base, with ventral outline of parameres 1.5× longer than greatest width near base; dorsal outline of aedeagus in lateral view obliquely straight nearly along basal 5/6.

##### Etymology.

Named after the Kawsay Biological Station, the type locality for this species, to honor the effort of Raul Bello to preserve this biodiverse area in Peru.

##### Distribution.

This species has been found in Peru and Ecuador (Fig. 23).

##### Habitat.

This species was collected from large forested pools with abundant detritus in seasonally flooded forest.

##### Remarks.

The dorsal plate of the median lobe of the aedeagus exhibits some variation in the degree of constriction at the base of the fork and in the shape of the arms of the fork. We examined one specimen from Ecuador that appears very similar to the Peruvian form (compare Fig. 19I vs. Fig. 19L) but because it differs in the degree of constriction and the shape of the arms of the fork, we have not included it in the paratype series.

#### ﻿ Novochares pastinum sp. nov.

Taxon classificationAnimaliaColeopteraHydrophilidae

C44A3F14-A82A-5E37-BE7B-D36464EF27CB

https://zoobank.org/F3453F99-893E-405C-A816-7DE2DC13ACB1

Figs 21A–D , 22A

##### Type material.

***Holotype* (male)**: ECUADOR: Sucumbíos/ Sacha Lodge, 0.5°S,/ 76.5°W, 270 m, 22II-4III/ 1994, Hibbs, ex: malaise” (SEMC). ***Paratype* (2 exs.): Ecuador**: Same data as holotype (1, SEMC). **Napo**: Lago Agrio, 26.vi.1978, leg. J. J. Anderson, swamp under secondary forest (1, USNM).

##### Differential diagnosis.

The aedeagal form of this species (Fig. 21A) somewhat resembles that of *N.quadrispinus* (Fig. 21E) but that species has four rather than two projections at the apex of the dorsal plate of the median lobe.

##### Description.

Body length 5.8 mm. ***Coloration***: Dorsal surfaces brown, with slightly paler (yellowish) margins of clypeus, pronotum and elytra. ***Head***: Maxillary palps nearly 1.5× width of head, uniformly brown or orange in color. ***Thorax***: Ground punctation on pronotum and elytra relatively dense and very shallowly impressed. Elytra without rows of serial punctures, each with very faint rows (one dorsal and two or three lateral) of scarce and weakly marked systematic punctures. Prosternum medially very weakly convex. Posterior elevation of mesoventrite weakly, broadly, and somewhat longitudinally elevated, with low medial longitudinal ridge extending anteriorly. ***Abdomen***: Apical emargination of fifth ventrite relatively narrow and deep, U-shaped. ***Aedeagus***: (Fig. 21A–D) Overall shape pear-like, 2.2× longer than wide, with outer lateral margins of parameres evenly convex up to apical region; apical region of each paramere rounded, with outer margin laterally pointed; at closest point, dorsal inner margins of parameres separated by distance 0.6× greatest width of a paramere; dorsal plate of median lobe with neck 0.6× as broad as base; arms of dorsal plate of median lobe parallel-sided along basal 1/2, diverging along distal 1/2, dorsally concave, nearly 0.2× length of dorsal plate of median lobe; each arm nearly uniformly wide along basal 1/2, then narrowing to narrowly rounded apex; notch between arms very narrow at base; gonopore placed near mid-length of dorsal plate of median lobe; ventral plate of median lobe membranous, extending to near base of fork of dorsal plate; basal piece 0.2× length of a paramere. In lateral view, aedeagus oblique at base, with ventral outline of parameres 3.5× longer than greatest width near basal 1/3; dorsal outline of aedeagus in lateral view concave along basal 1/3, then evenly convex up to apical region of parameres.

##### Etymology.

*Pastinum* (L.), a hoe, a two-pronged instrument used for digging, in reference to the two prongs of the dorsal plate of the median lobe.

##### Distribution.

Ecuador (Fig. 22A).

##### Habitat.

One specimen was collected in a swamp, the other two were taken in a malaise trap.

#### ﻿ Novochares pichilingue

Taxon classificationAnimaliaColeopteraHydrophilidae

(Fernández, 1989)

01D13F27-77CA-5B6C-B53A-6C45EBD50D40

Figs 17B , 21K, L , 22C

Helochares (s. str.) pichilingue Fernández, 1989: 147.
Novochares pichilingue (Fernández, 1989); Girón and Short 2021: 205.

##### Type material.

The unique holotype male is from Ecuador (Los Rios: Queveao, Rio Pichilingue) and deposited in MACN (not examined).

##### Material examined

**(56 exs.): Ecuador: Cotopaxi**: Latacunga (133 km W), 1080’, 2.vii.1975, at blacklight, leg. Langley & Cohen (1, USNM). **Esmeraldas**: La Union, 3.ii.1979, leg. J. J. Anderson, “UV att.” (11, USNM). **Guayas**: Daule, viii.1998, leg. A. Bandinelli (2, SEMC). **Los Ríos**: Quevedo, 11.v.1975, leg. Spangler, Gurney, Langley, & Cohen, blacklight (20, USNM, TTU-Z); 11 Km S Quevedo, 3.vii.1975, leg. Langley & Cohen, blacklight (1, UNSM); Babahoyo, 21.vi.1975, leg. Cohen, Langley, & Monnig, blacklight (7, USNM); same data except “large swampy pool w/ water hyacinth” (3, USNM). **Manabí**: Bahía de Caráquez (35 km SE), 10.v.1975, leg. Spangler, Langley, & Cohen, “weedy roadside pools” (1, USNM); Bahía de Caráquez (35.6 km E), 9.i.1978, leg. Spangler, culvert ditch (7, SEMC, USNM); 29 km S of Sto. Domingo, Rancho Ronald, 8.ix.1978, blacklight, leg. J.J. Anderson (1, USNM). **Pichincha**: Santo Domingo de los Colorados, 14 km E, 5.vii.1975, leg. Langley & Cohen (2, USNM), Santo Domingo de los Colorados, 29 km W, 7.v.1975, blacklight (2, USNM).

##### Differential diagnosis.

This species is one of the few that might be distinguished without dissection, due its distribution in a region with few other congeners, and due to its darkened pronotal disc (Fig. 17B) and relatively short maxillary palps. The form of the genitalia is also very distinctive and cannot be confused with any other species (Fig. 21K).

##### Description.

Body length 4.7–6.2 mm. ***Coloration***: Dorsal surfaces brown to dark brown, with very slightly paler (brown to yellowish) clypeus and margins of pronotum and elytra; dark labrum. ***Head***: Maxillary palps nearly 0.8× width of head, uniformly orange in color (Fig. 17B). ***Thorax***: Ground punctation on pronotum and elytra relatively dense and shallowly impressed. Elytra without rows of serial punctures, each with very faint rows (one dorsal and two or three lateral) of scarce and weakly marked systematic punctures. Prosternum medially weakly convex. Posterior elevation of mesoventrite broadly elevated, with very weak medial longitudinal ridge extending anteriorly. ***Abdomen***: Apical emargination of fifth ventrite relatively deep and broad, U-shaped. ***Aedeagus***: (Fig. 21K, L) Overall shape pear-like, 2.3× longer than wide, with outer lateral margins of parameres weakly and evenly convex; apical region of each paramere rounded, with outer margin laterally pointed; at closest point, dorsal inner margins of parameres separated by distance 0.58× greatest width of a paramere; dorsal plate of median lobe with neck 0.35× as broad as base; arms of dorsal plate of median lobe somewhat parallel, dorsally concave, nearly 0.4× length of dorsal plate of median lobe; each arm strongly broadened along basal 2/3, then narrowing to acute apex; notch between arms at base very narrow, dorsally cup-like; gonopore placed near base of dorsal plate of median lobe; ventral plate of median lobe membranous, extending to basal 2/3 of arms of dorsal plate; basal piece 0.3× length of a paramere. In lateral view, aedeagus weakly oblique at base, with ventral outline of parameres 2.9× longer than greatest width near base; dorsal outline of aedeagus in lateral view nearly straight along second 2/5.

##### Distribution.

Ecuador (Fig. 22C).

##### Habitat.

This species has been collected in pools and ditches, as well as at blacklights.

##### Remarks.

This species is currently only known from the western slopes of the Andes in Ecuador where it has been collected at a variety of localities. It is not known from the eastern (Amazonian) side.

#### ﻿ Novochares quadrispinus sp. nov.

Taxon classificationAnimaliaColeopteraHydrophilidae

51189FC4-30BA-50B7-8D19-3E5D77709302

https://zoobank.org/F9C91931-27D4-4F0B-9F9C-5ADB8E63C4CF

Figs 17D , 21E, F , 22D

##### Type material.

***Holotype* (male)**: SURINAME: Sipaliwini District/ 4.42313°N, 57.19198°W, 104 m/ Kabalebo Nature Resort/ Moi Moi Creek; 10-14-iii.2019/ Rock & detrital pools along creek/ Short & Class; SR19-0310-01A” (NZCS). ***Paratypes* (18 exs.): Brazil: Pará**: Rio Xingu Camp, Altamira (60 km S), 12.x.1986, leg. P. Spangler & O. Flint, pond at second palm grove on trail 1, Colln #21 (1, USNM); same data but 1.x.1986, jungle stream on trail 1, Colln. #3 (1, USNM). **Guyana: Region 6**: Upper Berbice Basecamp 1, 4°09.289'N, 58°10.717'W, 96 m, 21.ix.2014, leg, Short, Salisbury and La Cruz, muddy detrital pools in drying creekbed near camp, GY14-0921-02A (1, SEMC); Upper Berbice, N. Basecamp 2, 4°47.030'N, 58°01.850'W, 89 m, 28.ix.2014, leg. Short, Salisbury, & La Cruz, artificial pools by logging road, GY14-0928-01A (1, CBDG). **Region 7**: Takutu Mountains, 6°15'N, 59°5'W, 2-14.xii.1983, leg. P.D. Perkins (1, SEMC). **Suriname: Para**: along Martin Luther King Hwy, 5°32.856'N, 55°6.710'W, 2 m, 23.vii.2012, leg. Short et al., marsh by road, SR12-0723-02A (1, SEMC). **Sipaliwini**: Raleighvallen Nature Reserve, base of Voltzberg, 4°40.432’N, 56°11.079’W, 86 m, 16.iii.2016, leg. Short et al., pooled up stream, SR16-0316-01B (2, SEMC); Raleighvallen Nature Reserve, trail from plateau to Voltzberg, 17.iii.2016, leg. J. Girón, stream with roots and mud, SR16-0317-04A (3, NZCS, SEMC); Raleighvallen Nature Reserve, Coppename River, Voltzberg trail; detrital pools in stream bed, 17.iii.2016, leg. A. Short, SR16-0319-01A (1, SEMC); Raleighvallen Nature Reserve, Voltzberg Station, 4°40.910'N, 56°11.138'W, 78 m, stream margins, 30.vii.2012, leg. Short & McIntosh, detrital pools along stream, SR12-0730-01B (4, SEMC, including DNA vouchers SLE536 and SLE537, TTU-Z); same data as holotype except margins of detrital pool in drying creekbed, SR19-0310-01G (2, SEMC).

##### Differential diagnosis.

The four-pronged apex of the dorsal plate of the median lobe is unique in *Novochares*, no other species has four well-developed and apically acute projections (Fig. 21E).

##### Description.

Body length 5.9–7.0 mm. ***Coloration***: Dorsal surfaces brown to dark brown, with slightly paler (brown to orange) margins of clypeus, pronotum, and elytra. ***Head***: Maxillary palps nearly 1.3× width of head, uniformly brown to orange in color (Fig. 17D). ***Thorax***: Ground punctation on pronotum and elytra relatively dense and shallowly impressed. Elytra without rows of serial punctures, each with very faint rows (one dorsal and two or three lateral) of scarce and weakly marked systematic punctures. Prosternum medially very weakly convex. Posterior elevation of mesoventrite weakly, broadly, and somewhat longitudinally elevated, with low medial longitudinal ridge extending anteriorly. ***Abdomen***: Apical emargination of fifth ventrite relatively deep and broad, U-shaped. ***Aedeagus***: (Fig. 21E, F) Overall shape pear-like, 2.5× longer than wide, with outer lateral margins of parameres nearly evenly convex up to apical region; apical region of each paramere rounded, partly membranous, with outer margin very weakly pointed; at closest point, dorsal inner margins of parameres separated by distance 0.5× greatest width of a paramere; dorsal plate of median lobe with neck 0.6× as broad as base; neck dorsally concave; each arm of dorsal plate of median lobe with thin and narrow lateral branch; arms apically diverging and opposed, nearly 0.2× length of dorsal plate of median lobe; each arm apically and gradually narrowing to acute apex; notch between arms at base nearly 1/2 as wide as base of an arm; gonopore placed near mid-length of dorsal plate of median lobe; ventral plate of median lobe membranous, extending to second 1/3 of neck of dorsal plate; basal piece 0.4× length of a paramere. In lateral view, aedeagus oblique at base, with ventral outline of parameres 2× longer than greatest width near mid-length; dorsal outline of aedeagus in lateral view slightly concave along basal 1/3, then nearly straight to apical region; ventral outline of aedeagus in lateral view evenly convex up to apical region.

##### Etymology.

Quadrispinus, referring to the four-pointed apex of the dorsal plate of the median lobe.

##### Distribution.

Brazil (Para), Guyana, Suriname (Fig. 22D).

##### Habitat.

This species had been most commonly collected in pools in drying creek bed in the forest and other similar riparian detrital pools.

#### ﻿ Novochares sallaei

Taxon classificationAnimaliaColeopteraHydrophilidae

(Sharp, 1882)

2221638E-D20D-5206-9316-E52BD36E80AE

Figs 4C , 20M–R , 23

Helochares sallæi Sharp, 1882: 75.
Helochares (s. str.) sellae Sharp, 1882; Knisch 1924: 199 [catalog; misspelled].
Helochares (s. str.) sallaei Sharp, 1882; Hansen 1999: 163 [catalog].
Philhydrus estriatus Blatchley, 1917: 139; Winters 1927a: 24 [synonymy].
Novochares sallaei (Sharp, 1882); Girón and Short 2021: 205.

##### Type material examined.

***Novocharessallaei* Sharp, 1882: *Holotype* (female) by monotypy**: “Helocharessallaei/ type D. S./ Cordova, Mexico,/Sallaé” [on card with specimen], “Holo-type” [red disc], “Cordova”, “Mexico/ Sallé. Coll.”, “881” [upside down], “B.C. A. Col. I. 2./ Helochares/ sallaei,/ Sharp.”, “Helocharis castaneus, Chev/ [illegible word] Sallé” [label folded over], “Helochares/ sallaei Sharp/ M. E. Bacchus det. 1981/ Holotype”. The female genitalia are dissected and mounted next to the specimen (NHMUK).

***Philhydrusestriatus* Blatchley, 1917: *Lectotype* (male)**: “TYPE [red rectangle]”, “Dunedin, Fla./ W. S. B. Coll./ 1-12.1913”. “Purdue/ Blatchley/ Collection”, “LECTOTYPE/ Enochrus/ estriatus/ Blatchley/ Des. W.S. Blatchley 1930” (PERC). ***Paralectotype* (1 ex.)**: Same date as holotype (1 female, PERC).

##### Additional material examined

**(177 exs.). Belize: Belize District**: Western Hwy nr. Zoo, 7.i.2003, leg. C.R. Bartlett, Pine Grassland, light trap (1, SEMC). **Costa Rica: Limon Province**: Talamanca, Est. Gandoca, 22.v.2004, leg. Porras, Gamboa, Briceno, Morga, & Cardenas (2, SEMC). **Guatemala: Petén**: Parque Nacional El Rosario, E of Sayaxche, 16.52414°N, 90.16000°W, 30.vi.2014, leg. R.S. Zack, BL/MV lights (81, SEMC, WSU, including DNA voucher SLE1212). **Mexico: Campeche**: 14.9 mi S. of Champoton, Rte 180, 23.iv.1966, leg. G.E. Ball and D.R. Whitehead, Typha marsh (7, USNM); Zoh Laguna, 7–12.iv.1968, leg. Reyes & Cabrera (11, CAS). **Tamaulipas**: Tampico, 21.vi.1965, leg. Freytag & Gibson (1, USNM). **Veracruz**: 6 mi. N. Jesus Carranza (Isth. Tehuantepec), 200 ft., 25.vi.1961 (1, SEMC); La Granja, 30.vi.1964, leg. A.G. Baske, at light (1, SEMC); 0.8 mi. W Sontecomapan, 0–100 ft. elevation, 18–26.ix.1965, leg. G.E. Ball and D.R. Whitehead (1, USNM); 25 mi S. Acayucan, 4.vii.1965, leg. P.J. Spangler (69, USNM); Cordoba, 6–9.xi.1966, leg. A.B. Lau (1, USNM). **Yucatán**: Muna, 49 mi. S., 14.vii.1963 (1, SEMC). **USA: Florida**: Miami-Dade Co., Everglades National Park, vi.2000, leg. E.L. Nance (1, South Florida Collections Management Center [examined photos posted to Bugguide by M. Pintar]).

##### Differential diagnosis.

Throughout much of its range, this species only co-occurs with members of the *abbreviatus* species group (*N.abbreviatus* and *N.oculatus*) which both are much more brown/tan in dorsal coloration and have very different aedeagal forms. This species co-occurs in southern Central America with *N.chaquensis*, but the aedeagal forms are quite different (compare Fig. 20A vs. Fig. 20M). The small dorsally projecting ridge (Fig. 20P) along the inner margin of each paramere is also unique within *Novochares*.

##### Description.

Body length 6.4–8.0 mm. ***Coloration***: Dorsal surfaces brown to dark brown, with paler (brown to orange) margins of clypeus, pronotum, and elytra. ***Head***: Maxillary palps nearly 1.2× width of head, uniformly brown to orange in color. ***Thorax***: Ground punctation on pronotum and elytra relatively dense and shallowly impressed. Elytra without rows of serial punctures, each with very faint rows (one dorsal and two or three lateral) of scarce and weakly marked systematic punctures. Prosternum medially very weakly convex. Posterior elevation of mesoventrite weakly and broadly elevated, with low medial longitudinal ridge extending anteriorly. ***Abdomen***: Apical emargination of fifth ventrite relatively shallow and broad, U-shaped. ***Aedeagus***: (Figs 4C, 20M–R) Overall shape pear-like, 2.0–2.3× longer than wide, with outer lateral margins of parameres nearly evenly convex up to apical region; apical region of each paramere rounded, partly membranous, with outer margin smooth, not pointed; at closest point (near base of neck), dorsal inner margins of parameres separated by short distance or nearly touching each other; dorsal plate of median lobe with neck 0.3× as broad as base; neck dorsally concave; arms of dorsal plate of median lobe nearly parallel, from parallel-sided (Fig. 20Q) to broadened near mid-length (Fig. 20M), nearly 0.2× length of dorsal plate of median lobe; each arm acute or narrowly rounded at apex; notch between arms at base very narrow; gonopore placed at base of dorsal plate of median lobe; ventral plate of median lobe membranous, reaching base of neck of dorsal plate; basal piece 0.35× length of a paramere. In lateral view, aedeagus oblique at base, with ventral outline of parameres 3.6× longer than greatest width near mid-length; dorsal outline of aedeagus in lateral view slightly concave along basal 1/3, then nearly straight along second 1/3, and oblique to apex of parameres; ventral outline of aedeagus in lateral view straight.

##### Distribution.

Belize, Costa Rica, Guatemala (new record), Mexico, USA (Florida) (Fig. 23).

##### Habitat.

Though most specimens seem to have been collected at lights, the species has been collected in marshes.

##### Remarks.

This species occurs from Mexico south to Costa Rica. It is also known from Florida, where its status as a native or introduced species has remained unclear. The oldest known specimens from Florida were collected in 1913 (Blatchley 1917) from Dunedin in Pinellas County, along the central Gulf coast. Later, Young (1954) speculated that the species may have been introduced from Mexico from the lumber trade: “It is possibly a waif in Florida, having been transported by lumber ships from Mexico. (see L. J. Marchand, Fla. Acad. Sci., Quart. Jour., 9, 1948 [sic: 1946], for a discussion of the effects of the trade in cigar box lumber on the fauna of the Hillsborough River)” (Young 1954: 174).

The article that Young cites is about the occurrence of a crab that was only known from Tabasco, Mexico and later found also in the Tampa Bay area. That author (Marchand), found that logs had been imported from Tabasco to lumber mills in the Tampa Bay area and suggested the Florida population of the crab in Tampa was an introduction. However, the first importation of logs from Tabasco was in 1915 (Marchand 1946), while the first known Florida specimens of *N.sallaei* were collected in 1913 (from the Tampa area). This timeline makes it impossible that the cited lumber trade could be the cause of an introduction, and casts doubt on the introduction theory in general. Indeed, just a year later Young (1955) reported *N.sallaei* from the Everglades, a great distance away from Tampa and seemed to have second thoughts on his introduction hypothesis, stating “The rarity of the species in Florida caused me to conclude that it was a waif introduced by shipping from Mexico… To my chagrin, I now find that I have had at least seven specimens of *H.sallaei* in my collection for over a decade”.

After a review of available data, we find no basis for asserting that *N.sallaei* is an introduced species and therefore consider it native to the United States and the state of Florida.

#### ﻿ Novochares tridentis sp. nov.

Taxon classificationAnimaliaColeopteraHydrophilidae

2FAEF569-B78E-5387-ADD8-5700D5222133

https://zoobank.org/BF0BC6D8-BD38-4F9F-9EC8-5392DE19937E

Figs 21G–J , 22D

##### Type material.

***Holotype* (male): *Holotype* (male)**: “BRASIL: Goiás, Sta./ Isabel, R. Araguaia,/ Isla do Bananal/ I,8–11,1961./ B. Malkin leg.” (FMNH).

##### Differential diagnosis.

This species is unique among members of the *sallaei* species group by the 3-pronged appearance of the median lobe (Fig. 21G). There are other species with three prongs in the *tectiformis* species group (e.g., *N.trifurcatus*, Fig. 27I) but these species are larger in body size and the form of the aedeagus is much more robust.

##### Description.

Body length 5.1 mm. ***Coloration***: Dorsal surfaces brown, with paler (orange) clypeus and margins of pronotum and elytra. ***Head***: Maxillary palps only slightly longer than width of head, uniformly orange in color. ***Thorax***: Ground punctation on pronotum and elytra relatively dense and very shallowly impressed. Elytra without rows of serial punctures, each with very faint rows (one dorsal and two or three lateral) of scarce and weakly marked systematic punctures. Prosternum medially very weakly convex. Posterior elevation of mesoventrite transversely and weakly elevated, posteriorly concave, with low and broad medial longitudinal elevation extending anteriorly. ***Abdomen***: Apical emargination of fifth ventrite relatively shallow and broad, U-shaped. ***Aedeagus***: (Fig. 21G–J) Overall shape pear-like, 2.4× longer than wide, with outer lateral margins of parameres nearly evenly convex up to apical region; apical region of each paramere rounded, partly membranous, with outer margin laterally pointed; at closest point, dorsal inner margins of parameres separated by distance 0.6× greatest width of a paramere; dorsal plate of median lobe with neck 0.46× as broad as base; neck dorsally concave; arms of dorsal plate of median lobe nearly parallel along basal 1/2, converging along distal 1/2, nearly 0.26× length of dorsal plate of median lobe; each arm acute at apex; notch between arms at base 2× width of arm at base; gonopore placed near mid-length of dorsal plate of median lobe; ventral plate of median lobe moderately sclerotized, extending to second 1/3 of arms of dorsal plate, ventrally curved at apex; basal piece 0.3× length of a paramere. In lateral view, aedeagus oblique at base, with ventral outline of parameres 3.2× longer than greatest width near mid-length; dorsal outline of aedeagus in lateral view sinuate along basal 3/4, then nearly straight to apex of parameres; ventral outline of aedeagus in lateral view nearly straight, slightly bent at basal 1/3.

##### Etymology.

Named after the three-pointed appearance of the median lobe of the aedeagus, formed by the lateral arms of the dorsal plate and the median projection of the ventral plate.

##### Distribution.

Known only from the type locality in Brazil (Goiás) (Fig. 22D).

##### Habitat.

Nothing is known about the habitat of this species.

#### ﻿ Novochares unguis sp. nov.

Taxon classificationAnimaliaColeopteraHydrophilidae

5B9D3BA6-33F5-5A0B-92ED-7967B347B66D

https://zoobank.org/5E0EAE71-7821-4E09-A055-57A8095BE2C3

Figs 18J, K , 22D

##### Type material.

***Holotype* (male)**: “BOLIVIA: Santa Cruz Dept./ 3.7 km SSE Buena Vista/ Hotel Flora y Fauna/ 17°29'S, 63°33'W; A.R. Cline/ 1–12.v.2004; MV+UV lights (SEMC). ***Paratypes* (46 exs.): Bolivia**: “Bolivia” without additional data (1, USNM); **Santa Cruz**: Same data as holotype (14, SEMC); Inchilo Province, “Cafetal” by Rio Quebrada Palometilla, 5.viii.1990, forest clearing at UV light, leg. P. Parrillo & P. Bettella, “No. 001” (1, FMNH). **Peru: Cuzco**: Pilcopata, 600 m, 8–10.xii.1979 premontane moist forest, leg. J.B. Heppner (7, USNM); Villa Carmen Biological Station, South of Rio Pinipini, 27.v.2022, leg. Short et al., large marshy pool with detritus, PE22-0526-01E (2, MHNSM, SEMC, including DNA voucher SLE2460). **Madre de Dios**: Rio Tambopata Reserve, ca. 30 km SW Puerto Maldonado, 290 m, 16–20.xi.1979, leg. J.B. Heppner, subtropical humid forest (14, USNM [one is separated]); same data but 11–15.xi.1979 (4, USNM), same data but 2–5.xi.1979 (2, USNM); Tambopata, 12°50.204'S, 69°17.609'W, 208 m, Explorers Inn, S. Puerto Maldonado, “Ant trail”, 11.i.2020, leg. S. Baca, forest pools connected by small trickle, PE20-0111-01A (1, SEMC, DNA Voucher SLE2136).

##### Differential diagnosis.

See differential diagnosis for *N.clavieri*.

##### Description.

Body length 5.8–6.3 mm. ***Coloration***: Dorsal surfaces brown to dark brown, with paler (brown) margins of clypeus, pronotum, and elytra. ***Head***: Maxillary palps nearly 1.5× width of head, uniformly orange or brown in color. ***Thorax***: Ground punctation on pronotum and elytra relatively dense and very shallowly impressed. Elytra without rows of serial punctures, each with very faint rows (one dorsal and two or three lateral) of scarce and weakly marked systematic punctures. Prosternum medially very weakly convex. Posterior elevation of mesoventrite weakly, broadly, and somewhat longitudinally elevated, with low medial longitudinal ridge extending anteriorly. ***Abdomen***: Apical emargination of fifth ventrite shallow to moderately deep and broad, U-shaped. ***Aedeagus***: (Fig. 18J, K) Overall shape pear-like, 2× longer than wide, with outer lateral margins of parameres nearly evenly convex up to apical region; apical region of each paramere rounded, partly membranous, with outer margin very weakly pointed; at closest point, dorsal inner margins of parameres separated by distance 0.5× greatest width of a paramere; dorsal plate of median lobe with neck 0.57× as broad as base; neck dorsally concave; arms of dorsal plate of median lobe apically diverging, nearly 0.17× length of dorsal plate of median lobe; each arm slightly outwardly curved, apically and gradually narrowing to narrowly rounded apex; notch between arms at base slightly wider than base of an arm; gonopore placed at base of fork of dorsal plate of median lobe; ventral plate of median lobe not visible; basal piece 0.4× length of a paramere. In lateral view, aedeagus oblique at base, with ventral outline of parameres 3.7× longer than greatest width near base; dorsal outline of aedeagus in lateral view nearly evenly convex; ventral outline of aedeagus in lateral view nearly straight up to apical region.

##### Etymology.

*Unguis* (L.), meaning claws, referring to the curved, insect-claw-like form of the apex of the dorsal plate of the median lobe.

##### Distribution.

Bolivia, Peru (Fig. 22D).

##### Habitat.

Known from forested pools and streams margins.

#### ﻿ Novochares yanomami sp. nov.

Taxon classificationAnimaliaColeopteraHydrophilidae

9A446B43-48A2-584B-802C-14E8651D60D2

https://zoobank.org/32F865B4-110C-4C22-98A0-3CA69D012F00

Figs 18C–E , 23

##### Type material.

***Holotype* (male)**: “VENEZUELA: Amazonas State/ 0°50'N, 66°9'44"W; 140m/ Cerro de la Neblina, Basecamp;/ 12–20.ii.1984; leg. D. Davis/ & T. McCabe” (USNM).

##### Differential diagnosis.

See differential diagnosis for *N.bisinuatus*.

##### Description.

Body length 5.1 mm. ***Coloration***: Dorsal surfaces reddish brown, with slightly paler (reddish) clypeus and margins of pronotum and elytra. ***Head***: Maxillary palps nearly 1.8× width of head, uniformly reddish brown in color. ***Thorax***: Ground punctation on pronotum and elytra relatively dense and shallowly impressed. Elytra without rows of serial punctures, each with very faint rows (one dorsal and two or three lateral) of scarce and weakly marked systematic punctures. Prosternum medially very weakly convex. Posterior elevation of mesoventrite weakly, broadly, and somewhat longitudinally elevated, with low medial longitudinal ridge extending anteriorly. ***Abdomen***: Apical emargination of fifth ventrite shallow to moderately deep and broad, U-shaped. ***Aedeagus***: (Fig. 18C–E) Overall shape pear-like, 1.76× longer than wide, with outer lateral margins of parameres evenly convex up to apical region; apical region of each paramere rounded, partly membranous, with outer margin weakly pointed; at closest point, dorsal inner margins of parameres separated by distance 0.28× greatest width of a paramere; dorsal plate of median lobe with neck 0.33× as broad as base; arms of dorsal plate of median lobe dorsally concave, nearly 0.38× length of dorsal plate of median lobe; each arm basally diverging, apically converging, somewhat evenly broad along entire length, apically rounded and weakly pointing dorsally; notch between arms at base slightly narrower than base of an arm; gonopore placed at base of dorsal plate of median lobe; ventral plate of median lobe not visible; basal piece 0.36× length of a paramere. In lateral view, aedeagus oblique at base, with ventral outline of parameres 2.6× longer than greatest width near mid-length; dorsal outline of aedeagus in lateral view sinuate; ventral outline of aedeagus in lateral view nearly straight along basal 3/4.

##### Etymology.

Yanomami, in reference to the Yanomami indigenous group that inhabits the region where this species has been collected.

##### Distribution.

Only known from the type locality in southern Venezuela (Fig. 23).

##### Habitat.

Nothing is known about the habitat of this species.

###### ﻿*Novocharestectiformis* species group

**Species group diagnosis.** Body length 6.2–9.5 mm. ***Aedeagus***: (Figs 5, 25–28) Overall shape and relative length variable, joint basal margins of parameres broadly rounded; outer margin and apical region of each paramere variable; parameres longer than median lobe; apex rounded or truncate; parameres with apical region variable in degree of sclerotization; dorsal inner margin of each paramere highly variable; dorsal plate of median lobe (in dorsal view) with usually stout and strongly sclerotized basal apodemes; dorsal plate of median lobe highly variable, usually narrower along mid-section than at base and apex, sometimes forming a narrow neck (Fig. 27A, F; at narrowest, neck 0.1× as broad as dorsal plate of median lobe at base); notch between arms variable; shape and orientation of arms variable; gonopore sitting proximal to base of median lobe; ventral plate of median lobe (in ventral view) somewhat triangular, variable in length, shape of apex, and degree of sclerotization; dorsal surface of ventral plate of median lobe slightly concave (sides curved dorsally); basal piece nearly 0.25–0.35× length of a paramere, with distal margin concave. In lateral view, aedeagus triangular, strongly oblique at base, with ventral outline of parameres 3–5× longer than greatest width near base.

**Figure 24.**
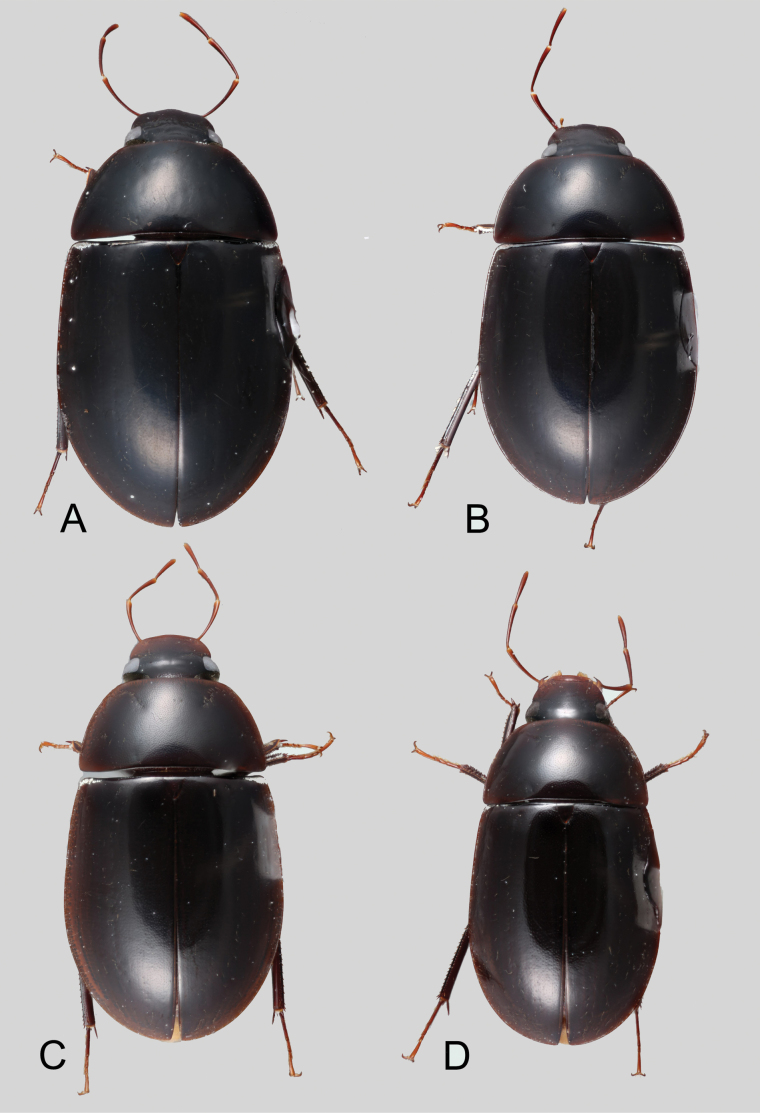
Dorsal habitus of *Novochares* spp. **A***N.tectiformis***B***N.orchis***C***N.duo***D***N.piaroa*.

**Figure 25.**
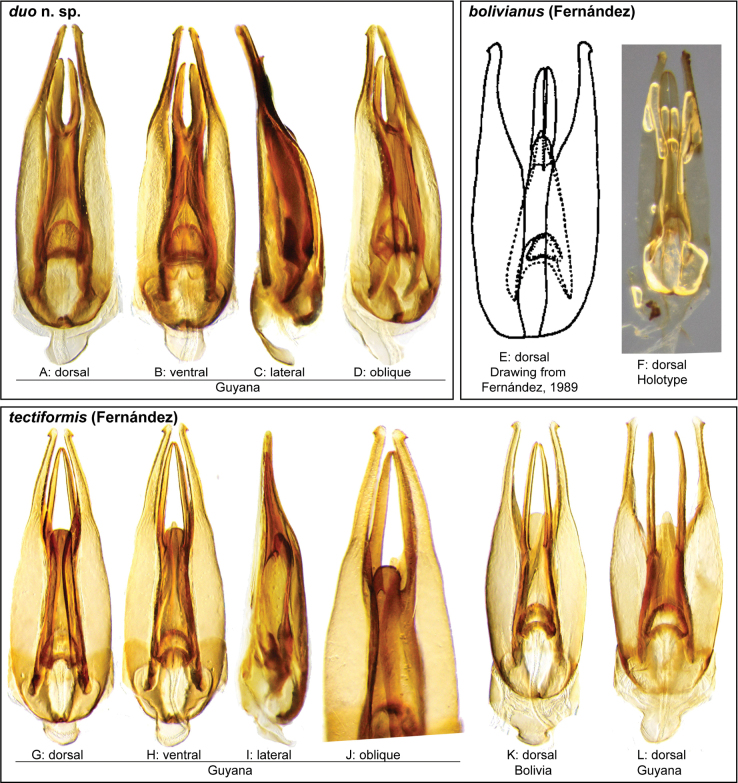
Aedeagi of the *Novocharestectiformis* species group **A–D***N.duo***E, F***N.bolivianus***G–L***N.tectiformis***A, E, F, G, K, L** dorsal view **B, H** ventral view **C, I** lateral view **D, J** oblique view.

**Figure 26.**
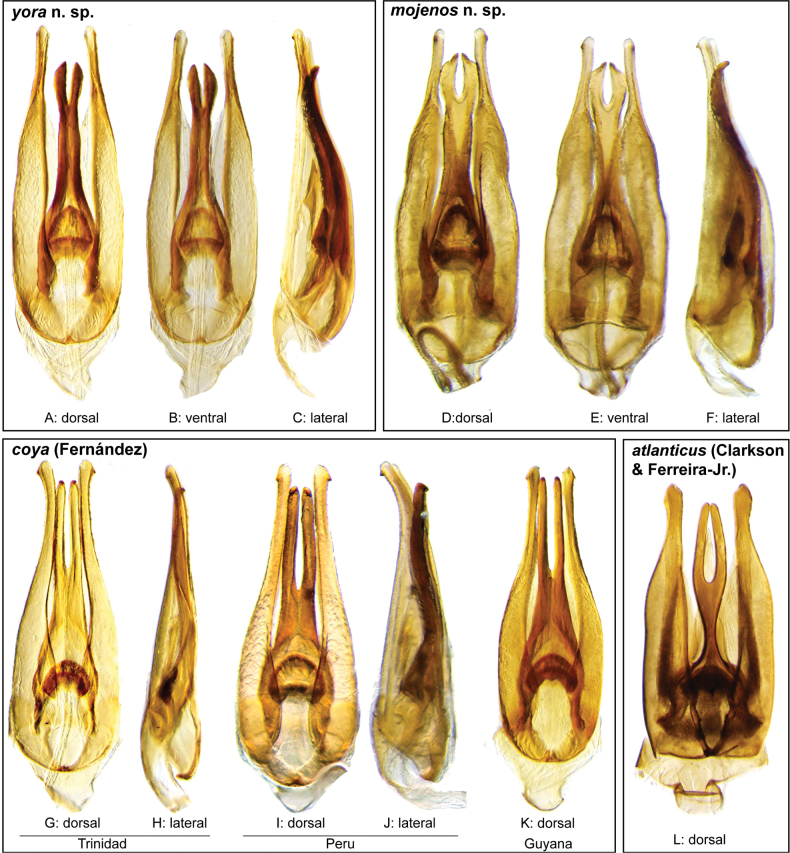
Aedeagi of the *Novocharestectiformis* species group **A–C***N.yora***D–F***N.mojenos***G–K***N.coya***L***N.atlanticus***A, D, G, I, K, L** dorsal view **B, E** ventral view **C, F, H, J** lateral view **G, H** Trinidad **I, J** Peru **K** Guyana.

**Figure 27.**
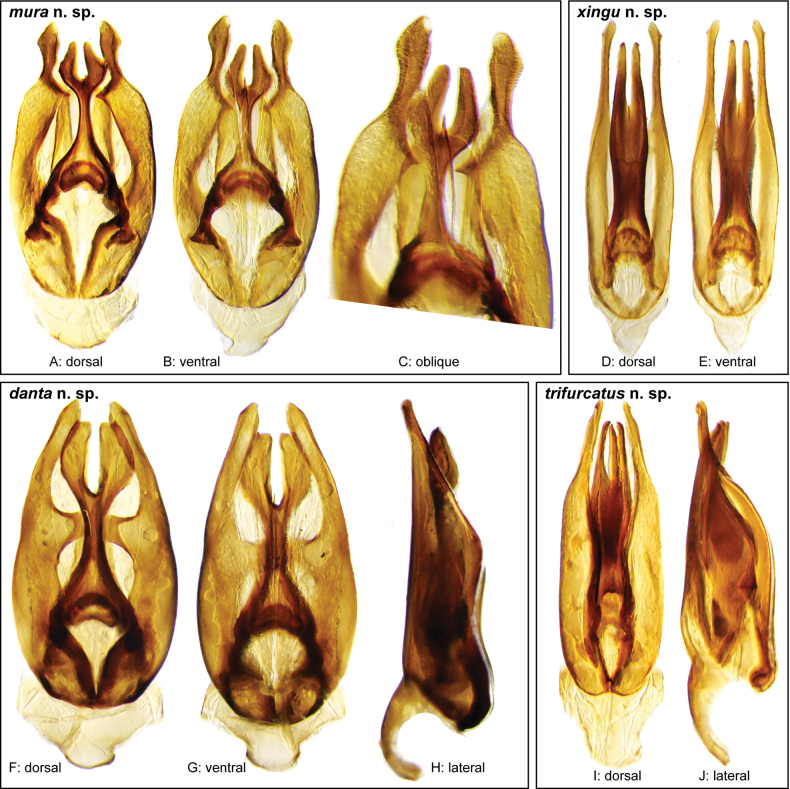
Aedeagi of the *Novocharestectiformis* species group **A–C***N.mura***D, E***N.xingu***F–H***N.danta***I, J***N.trifurcatus***A, D, F, I** dorsal view **B, E, G** ventral view **C** oblique view **H, J** lateral view.

**Figure 28.**
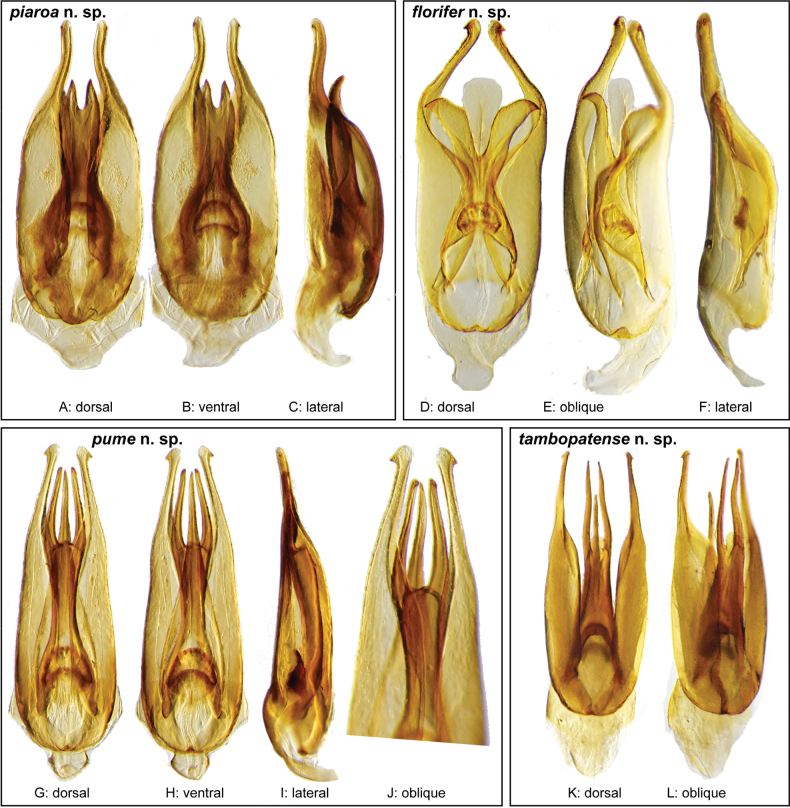
Aedeagi of the *Novocharestectiformis* species group **A–C***N.piaroa***D–F***N.florifer***G–J***N.pume***K, L***N.tambopatense***A, D, G, K** dorsal view **B, H** ventral view **C, F, I** lateral view **E, J, L** oblique view.

**Composition.** The *Novocharestectiformis* species group is composed of *Novocharesatlanticus* (Clarkson & Ferreira Jr, 2014), *N.bolivianus* (Fernández, 1989), *N.coya* (Fernández, 1982), *N.danta* sp. nov., *N.duo* sp. nov., *N.florifer* sp. nov., *N.mojenos* sp. nov., *N.mura* sp. nov., *N.piaroa* sp. nov., *N.pume* sp. nov., *N.tectiformis* (Fernández, 1982), *N.trifurcatus* sp. nov., *N.xingu* sp. nov., and *N.yora* sp. nov.

#### ﻿ Novochares atlanticus

Taxon classificationAnimaliaColeopteraHydrophilidae

(Clarkson & Ferreira Jr, 2014)

FFBCC2EB-5B6C-519D-8B3C-0AE538F51248

Figs 26L , 29A

Helochares (s. str.) atlanticus Clarkson & Ferreira Jr, 2014: 402.
Novochares atlanticus (Clarkson & Ferreira Jr); Girón and Short 2021: 203.

##### Type material.

***Holotype*** male from Brazil (São Paulo, Ubatuba, Parque Estadual da Serra do Mar, Nucleo Picinguaba) and deposited in DZRJ (not seen).

##### Material examined

**(1 ex.). Brazil: São Paulo**: Ubatuba, Parque Estadual da Serra do Mar, Nucleo Picinguaba, Pocas no caminho, de Casa de Furinha, 29.v.2010, leg. Ferreira Jr. & N. [Hebergr?] (1, DZRJ).

##### Differential diagnosis.

The aedeagus of this species is not exceptionally close to any others. It is superficially similar to *N.coya* (Fig. 26G) but *N.atlanticus* has the neck of the dorsal plate of the median lobe strongly constricted, and the outer margins of the parameres are nearly straight along their basal 1/2 (Fig. 26L).

##### Description.

Body length 8.9 mm. ***Coloration***: Dorsal surfaces dark brown, with slightly paler (orange-brown) margins of clypeus, pronotum and elytra. ***Head***: Maxillary palps 1.3× longer than width of head, reddish brown in color, slightly paler (orange) at apex of each palpomere. ***Thorax***: Ground punctation on pronotum and elytra dense and very shallowly impressed. Elytra without rows of serial punctures, each with very faint rows (one dorsal and two or three lateral) of scarce and weakly marked systematic punctures. Prosternum only very weakly medially convex. Posterior elevation of mesoventrite elevated as a triangular pyramid, with posterior face somewhat bisinuate and medial longitudinal ridge extending anteriorly (resembles a nose). ***Abdomen***: Apical emargination of fifth ventrite relatively wide, V-shaped. ***Aedeagus***: (Fig. 26L) Overall shape sub-rectangular, 2.0–2.3× longer than wide; lateral projection on apical region of outer margin of each paramere pointed; at closest point, dorsal inner margins of parameres separated by distance nearly 1/2 greatest width of a paramere; dorsal plate of median lobe with narrow neck along mid section; arms of dorsal plate of median lobe distally converging, 0.3–0.4× length of dorsal plate of median lobe; each arm parallel sided, apically rounded; notch between arms at base as broad as base of an arm; ventral plate of median lobe triangular, apically acuminate, apex extending between mid-length to second 1/3 of arms of dorsal plate; basal piece 0.3–0.4× length of a paramere. In lateral view, aedeagus weakly oblique at base, with ventral outline of parameres 3.4× longer than greatest width near base; dorsal outline of aedeagus in lateral view nearly straight along basal 2/3.

##### Distribution.

**Figure 29.**
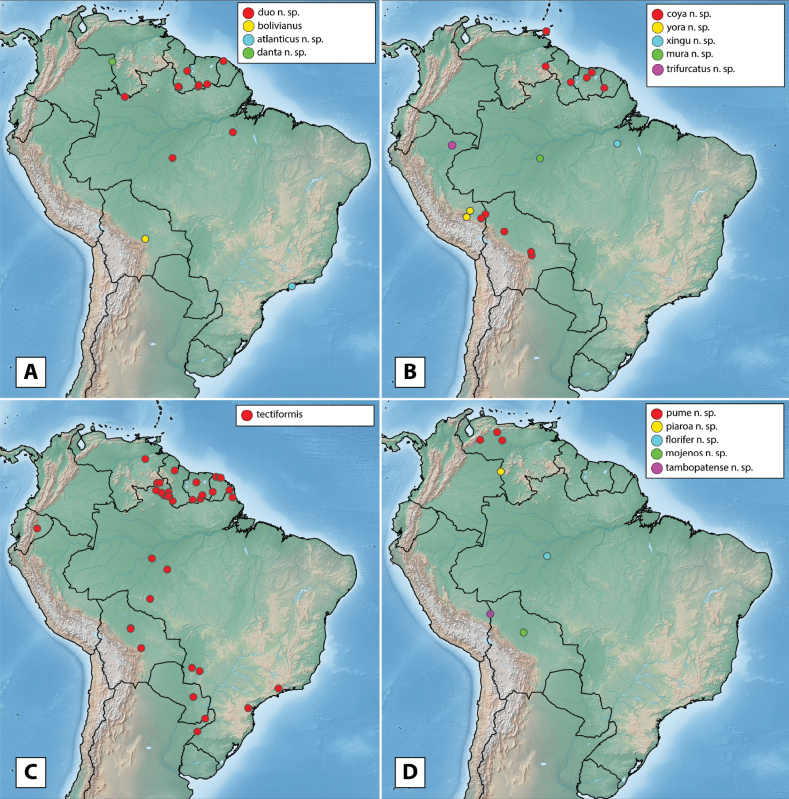
Distribution of *Novocharestectiformis* species group **A***N.duo* (red), *N.bolivianus* (yellow), *N.atlanticus* (blue), *N.danta* (green) **B***N.coya* (red), *N.yora* (yellow), *N.xingu* (blue), *N.mura* (green), *N.trifurcatus* (purple) **C***N.tectiformis* (red) **D***N.pume* (red), *N.piaroa* (yellow), *N.florifer* (blue), *N.mojenos* (green), *N.tambopatense* (purple).

Known from several localities in São Paulo and Rio de Janeiro States, Brazil (Fig. 29A).

##### Habitat.

Specimens have been collected from “...temporary ponds that have leaf litter and aquatic vegetation. These ponds were covered and shaded in the border of the forest, or were in open areas”. Localities ranged from sea level to 1150 m in elevation.

##### Remarks.

The specimen we examined and figured here is from the type locality.

#### ﻿ Novochares bolivianus

Taxon classificationAnimaliaColeopteraHydrophilidae

(Fernández, 1989)

2CB3C309-0C6A-54DB-BD5C-AE949440CC0C

Figs 25E, F , 29A

Helochares (s. str.) bolivianus Fernández, 1989: 146, 148 [in key].
Novochares bolivianus (Fernández); Girón and Short 2021: 204.

##### Type material examined.

***Holotype* (male)**: We examined images of the holotype, including the dissected aedeagus. The specimen is from Bolivia (Santa Cruz Department, Gutiérrez Province, Nueva Moka) and deposited in MACN.

##### Differential diagnosis.

See differential diagnosis for *N.duo*.

##### Description.

***Aedeagus***: (Fig. 25E, F) Overall shape pear-like, 2.4× longer than wide; lateral projection on apical region of outer margin of each paramere pointed; at closest point, dorsal inner margins of parameres separated by distance nearly 0.5× greatest width of a paramere; dorsal plate of median lobe with arms parallel, nearly 0.5× length of dorsal plate of median lobe; each arm parallel sided, apically rounded; notch between arms very narrow; ventral plate of median lobe triangular, apically rounded, apex extending to base of arms of dorsal plate.

##### Distribution.

Known only from the type locality in Bolivia (Fig. 29A).

##### Habitat.

Nothing is known about the habitat of this species.

##### Remarks.

This species is known from a single male specimen. We examined images of the type specimen and its aedeagus (Fig. 25E, F). While we considered that it may in fact refer to *N.duo*, the differences in the length of the ventral plate of the median lobe of the aedeagus and the disjunct distributions led us to consider them distinct species.

#### ﻿ Novochares coya

Taxon classificationAnimaliaColeopteraHydrophilidae

(Fernández, 1982)

E3E216D0-D5B2-5364-AB18-862F2F99715D

Figs 26G–K , 29B

Helochares (s. str.) coya Fernández, 1982: 87; Fernández 1989: 148 [in key].
Novochares coya (Fernández); Girón and Short 2021: 204.

##### Type material.

***Holotype***: male from Bolivia (Santa Cruz Department, Sara Province, Monteros) and deposited MLP (not seen).

##### Material examined

**(58 exs.). Bolivia: Beni**: Beni Station, Palm Camp, 1.viii.1988, leg. R.W. Brooks, at lights (2, SEMC); same data but also “NE of San Borja” (1, SEMC). **Santa Cruz**: Ayacucho, 13–14.v.1969, leg. P. & P. Spangler (1, USNM). **French Guiana**: Anapaike Village, Lawa River, 22–25.ix1963, leg. B. Malkin (3, USNM). **Guyana: Region 6**: Upper Berbice, ca. 1 km W. Basecamp 1, 105 m elev., small detrital side pools, 22.ix.2014, leg. Short, GY14-0921-03G (2, CBDG, SEMC, including DNA Voucher SLE1218). **Peru**: [no other locality data], 22.xi.1935, leg. F. Woytkowski (1, SEMC). **Huanuco**: Tingo Maria, 670 m, 1–10.v.1937, leg. F. Woytkowski (7, SEMC). **Jauja**: Junin Department, Viena, 2300 m, 1–6.viii.1935, leg. F. Woytkowski (1, SEMC). **Madre de Dios**: Rio Tambopata Reserve, ca. 30 km SW Puerto Maldonado, 290 m, various dates between 2–25.xi.1979, leg. J.B. Heppner, subtropical humid forest (21, USNM); Villa Carmen Biological Station (ca. 2 km N of Pilcopata), South of Rio Piñipiñi, 29.v.2022, leg. Short et al., small marsh with dense vegetation, PE22-0526-01G (1, SEMC); same data but pools in dirt road near station, PE22-0526-01B (1, SEMC); Kawsay Biological Station (ca. 19 km E of Puerto Maldonado), 3.vi.2022, leg. Short et al., swamp pool near banana area, PE22-0603-02B (1, SEMC); same data but 4.vi.2022, forested swamp, PE22-0604-01C (1, SEMC). **Suriname: Saramacca**: Coesewinje River at Coesewinje Savanna, shallow river margin and backwaters, 6.iii.2012, leg. Short & Kadosoe, SR12-0306-03A (1, SEMC); **Sipaliwini**: Raleighvallen Nature Reserve, Coppename River-Voltzberg Trail, 19.iii.2016, leg. A.E.Z. Short, detrital pools in small stream bed, SR16-0319-01A (1, SEMC); Raleighvallen Nature Reserve Lolopaise area, 4°42.48’N, 56°13.15908’W, 24 m, 18.iii.2016, leg. Short et al., intermittent stream margins and flotation, SR16-0318-01D (1, NZCS, SEMC). **Trinidad and Tobago**: Trinidad, St. Helena, 24.xi.1931, leg. W.E. Broadway (8, SEMC); [without further locality] 31.v.1931, leg. W.E. Broadway (1, SEMC). **Venezuela: Bolívar**: El Dorado (65 km S.), 1.xi.1982, leg. J.L. Hellman (4, USNM).

##### Differential diagnosis.

See differential diagnosis for *N.atlanticus*.

##### Description.

Body length 6.5–9.1 mm. ***Coloration***: Dorsal surfaces dark brown, with slightly paler (brown or reddish brown) clypeus and margins of pronotum and elytra, sometimes sheeny. ***Head***: Maxillary palps 1.3–1.5× longer than width of head, uniformly orange in color. ***Thorax***: Ground punctation on pronotum and elytra dense and very shallowly impressed. Elytra without rows of serial punctures, each with very faint rows (one dorsal and two or three lateral) of scarce and weakly marked systematic punctures. Prosternum nearly flat to only very weakly medially convex. Posterior elevation of mesoventrite elevated as a triangular pyramid, with posterior face somewhat bisinuate and medial longitudinal ridge extending anteriorly (resembles a nose). ***Abdomen***: Apical emargination of fifth ventrite relatively deep, U-shaped. ***Aedeagus***: (Fig. 26G–K) Overall shape pear-like; 3.2× longer than wide; lateral projection on apical region of outer margin of each paramere pointed; at closest point, dorsal inner margins of parameres separated by distance nearly 1/2 as greatest width of a paramere; dorsal plate of median lobe with neck 1/2 as broad as base; arms of dorsal plate of median lobe parallel to slightly converging, nearly 0.35× length of dorsal plate of median lobe; each arm parallel sided, with apex rounded and dorsally pointed; notch between arms at base nearly as broad as base of an arm or slightly narrower; ventral plate of median lobe weakly sclerotized, at widest point nearly as wide as dorsal plate of median lobe, apically acuminate, apex extending beyond base of fork, not reaching apex of arms of dorsal plate; basal piece 0.35× length of a paramere. In lateral view, aedeagus strongly flattened, with ventral outline of parameres 5× longer than greatest width near base.

##### Distribution.

Previously only known from Bolivia, here newly recorded from French Guiana, Guyana, Peru, Suriname, Trinidad and Tobago, and Venezuela (Fig. 29B).

##### Habitat.

This species has been collected in forested pools with abundant detritus, including those associated with the margins of streams or drying creek beds.

##### Remarks.

Though there is a somewhat large gap in the known distribution of this species, with one large group of records in the northeast region of South America, and another in Peru and Bolivia there are no appreciable differences in the aedeagus (Fig. 26G–K) or COI sequence data (Fig. 1). There is one male specimen from Jauja Province (Peru) that was collected at 2300 meters, which represents the highest elevation that *Novochares* has been found.

#### ﻿ Novochares danta sp. nov.

Taxon classificationAnimaliaColeopteraHydrophilidae

D92B238B-679A-56CD-9F4E-24950E292218

https://zoobank.org/743936D3-F4ED-4F50-9CB0-9546AEC2FEBA

Figs 27F–H , 29A

##### Type material.

***Holotype* (male)**: “VENEZUELA: Amazonas State/ 5°20.514'N, 67°45.315'W, 87m/ S. Communidad Porvenir/ 15.i.2009; leg. Miller & Short/ VZ09-0115-03B; small streamlet” (MIZA). ***Paratypes* (9 exs.): Venezuela: Amazonas**: Same data as holotype (7, MIZA, SEMC, including DNA Voucher SLE1399); W. Comunidad La Danta, 15.i.2009, leg. Short, Miller, & Camacho, detrital margin of stream, VZ09-0115-04B (2, SEMC).

##### Differential diagnosis.

The genitalia of this species is spectacular (Fig. 27F) and not even close to any other *Novochares* species except *N.mura* (Fig. 27A). They both share the same very broad form, with a distinct projection along the inner margin of the parameres, which is acute and more distal in *N.mura*, and quadrate and more proximal in *N.danta*.

##### Description.

**Size and form**: Body length 6.6–8.0 mm. ***Coloration***: Dorsal surfaces dark brown, sometimes with slightly to moderately paler (brown or reddish brown) clypeus and margins of pronotum and elytra, sometimes sheeny. ***Head***: Maxillary palps nearly 1.4× longer than width of head, uniformly orange to reddish brown in color. ***Thorax***: Ground punctation on pronotum and elytra dense and very shallowly impressed. Elytra without rows of serial punctures, each with very faint rows (one dorsal and two or three lateral) of scarce and weakly marked systematic punctures. Prosternum nearly flat to only very weakly medially convex. Posterior elevation of mesoventrite elevated as a triangular pyramid, with posterior face somewhat posteriorly concave and medial longitudinal ridge extending anteriorly (resembling a nose). ***Abdomen***: Apical emargination of fifth ventrite relatively deep, U-shaped. ***Aedeagus***: (Fig. 27F–H) Overall shape oval, 1.8× longer than wide; apical region of parameres somewhat triangular, roundly pointed; dorsal inner margin of each paramere with a large mesal projection, apically truncate, proximal to fork of dorsal plate of median lobe; dorsal plate of median lobe with base 4× broader than neck; arms of dorsal plate of median lobe dorsally concave, widened along basal 2/3, nearly 0.28× length of dorsal plate of median lobe, with inner margins parallel; each arm apically truncate, pointed at outer corner; notch between arms at base slightly narrower than base of an arm; ventral plate of median lobe moderately sclerotized, triangular, with curved lateral margins, apically narrowly and sharply acuminate, apex extending slightly beyond base of fork; basal piece 0.28× length of a paramere. In lateral view, aedeagus parallelogram-shaped, with ventral outline of parameres 3× longer than greatest width near base; dorsal outline of aedeagus in lateral view sinuate, medially concave.

##### Etymology.

Named after the indigenous community from where this species was collected.

##### Distribution.

Known only from two closely situated localities in the Guiana Shield region of southern Venezuela (Fig. 29A).

##### Habitat.

This species was collected from the margins of densely forested streams.

#### ﻿ Novochares duo sp. nov.

Taxon classificationAnimaliaColeopteraHydrophilidae

B5865AD9-7552-5814-AE01-8F6E018024BD

https://zoobank.org/4E083291-ECF8-4248-8406-2589BB6DA2B9

Figs 24C , 25A–D , 29A

##### Type material.

***Holotype* (male)**: “GUYANA: Region IX/ 2°05.095'N, 59°14.174'W, 250m/ Parabara, Trail to mines/ detrital pools in forest/ leg. Short, Isaacs, Salisbury/ 2.xi.2013; GY13-1102-01A (CBDG). ***Paratypes* (206 exs.): Brazil: Amazonas**: Apui (ca. 43 km NW), 4.vii.2018, leg. Short, backwater river margin w/detritus and leaf packs, BR18-0704-02C (4, INPA, SEMC, including DNA voucher SLE1906). **Pará**: Rio Xingu Camp, Altamira (60 km S), 12.x.1986, leg. P. Spangler & O. Flint, pond at second palm grove on trail 1, Colln #21 (7, USNM); same data but 15.x.1986, Colln. #24 (4, USNM); same data but 14.x.1986, stream on left branch of trail 1, Colln. #23 (1, USNM). **French Guiana**: Piste de montagne de fer (formerly road Degrad Florian), 5.40697°N, -53.55468°W, 10 m, leg. Short & Neff, forested detrital pools, FG20-0305-01A (5, SCC, SEMC). **Guyana: Region 6**: Upper Berbice Basecamp 1, 4°09.289'N, 58°10.717'W, 96 m, 21.ix.2014, leg, Short, Salisbury and La Cruz, muddy detrital pools in drying creekbed near camp, GY14-0921-02A (9, SEMC); Upper Berbice circa 1 km west of Basecamp 1, 4°09.143'N, 58°11.207'W, 105 m, 21.ix.2014, leg. A. Short, sandy stream, GY14-0921-03A (1, SEMC); same data but detrital side pools, GY14-0921-03G (2, SEMC); Upper Berbice, ca. 1.1 Km W of basecamp 1, 4°09.136'N, 58°11.365'W, 106 m, stream detrital pool, 23.ix.2014, GY14-0923-02A (1, SEMC); Upper Berbice Basecamp 1, 4°09.289'N, 58°10.717'W, 96 m, 24.ix.2014, leg. Short, Salisbury, and La Cruz, margins of basecamp creek, GY14-0924-01A (2, SEMC); same data but 4°09.241'N, 58°10.627'W, 109 m puddles along road, GY14-0924-02A (3, SEMC); Upper Berbice ca. 1 km south of Basecamp 1, 4°09.241'N, 58°10.627'W, 109 m, 25.ix.2014, leg. Short, Salisbury, and La Cruz, detritus pools in dry creekbed, GY14-0925-01D (3, SEMC). **Region 9**: same data as holotype (15, SEMC, including DNA Voucher SLE1209); North of Parabara, Bototo Wau Creek, 2°10.908'N, 59°20.306'W, 289 m, 31.x.2013, leg. Short, Isaacs and Salisbury, stream margins, GY13-1031-01A (20, SEMC); along road to Parabara, 2°09.557'N, 59°17.569'W, 268 m, 1.xi.2013, leg. Short, Isaacs and Salisbury, forest pools near Mushai Wao, GY13-1101-02A (3, SEMC); Parabara, trail to mines, 2°05.095'N, 59°14.174'W, 250 m, 2.xi.2013, leg. Short, Isaacs and Salisbury, detrital pools in forest, GY13-1102-01A (21, SEMC, TTU-Z); Parabara, trail on N. side of river, 3.xi.2013, leg. Short, small detrital pool in forest, GY13-1103-01A (1, SEMC); Parabara north side of river, 2°06.492'N, 59°13.653'W, 274 m, 3.xi.2013, detritus margins and leaf packs, GY13-1103-02A (10, SEMC); Parabara, at N. edge of village, 2°05.733'N, 59°14.390'W, 248 m, leg. Short, Isaacs and Salisbury, small vegetated marsh, GY13-1103-03A (31, SEMC); N. Parabara, basecamp area, 2°10.902'N, 59°20.547'W, 260 m, leg. Short, Isaacs and Salisbury, small sandy stream with root mats and leaf packs, GY13-1105-01B (10, SEMC) same locality but 1–5.xi.2013, light trap in savanna, GY13-1101-LT2 (1, SEMC). **Suriname: Sipaliwini**: Camp 1 on Kutari River, 2°10.521'N, 56°47.244'W, 228 m, 20.viii.2010, leg. Short and Kadosoe, forest stream, CI-RAP Survey, forested swamp, SR10-0819-01A (16, NZCS, SEMC, TTU-Z, including DNA Voucher SLE1209); Iwaana Saamu, forest swamp, 26.viii.2010, leg. Short, SR10-0826-01A (3, SEMC); Camp 2 on Sipaliwini River, 2°10.973'N, 56°47.235'W; 210 m, 30.viii.2010, Short and Kadosoe, forest creek, SR10-0831-01A (3, SEMC); same data except: 31.viii.2010, sandy forest creek with detritus, SR10-0831-01B (1, SEMC); Camp 3, Werehpai, 2°21.776'N, 56°41.861'W, 237 m, 3–7.ix.2010, leg. Short and Kadosoe, pooled up detrital creek, SR10-0903-01A (4, SEMC); except detrital forest pools, SR10-0903-02A (3, SEMC); same data except sandy forest creek, SR10-0904-01A (1, SEMC); Camp 1, Upper Palumeu, 2.47700°N, 55.62941°W, 275 m, 10–12.iii.2012, leg. A. Short, large detrital pools, SR12-0310-01A (1, SEMC, DNA voucher SLE1211); same data except 10.iii.2012, small forest pool, SR12-0310-02A (2, SEMC); same data except 11.iii.2012, large pool by trail, SR12-0311-01A (1, SEMC). **Venezuela: Amazonas**: Cerro de la Neblina 1.5 km S. Basecamp, 0°50'N, 66°10'W, 250 m, 15.ii.1985, leg. P.J. Spangler & P.M. Spangler, R. Faitoute, & W. Steiner, in small ponds full of dead leaves, rainforest ridge (14, USNM); same data but 7.ii.1985 (2, USNM); same locality but 8.ii.1985, leg. Steiner & Halling, small whitewater stream in rainforest (1, USNM).

##### Differential diagnosis.

The genitalia of this species is similar to several other related species in the *tectiformis* species group. It is perhaps most similar to *N.bolivianus*, but in that species the fork of the apex of the dorsal plate of the median lobe is very narrow, with no gap between the two projections (Fig. 25E). Additionally, in that species the ventral plate is extended further, beyond the base of the fork, while it is much shorter and does not reach the fork in *N.duo* (Fig. 25A, B).

##### Description.

Body length 6.5–9.0 mm. ***Coloration***: Dorsal surfaces dark brown, sometimes with slightly to moderately paler (brown or yellowish brown) clypeus and margins of pronotum and elytra, sometimes sheeny. ***Head***: Maxillary palps nearly 1.4–1.6× longer than width of head, uniformly orange in color (Fig. 24C). ***Thorax***: Ground punctation on pronotum and elytra dense and very shallowly impressed. Elytra without rows of serial punctures, each with very faint rows (one dorsal and two or three lateral) of scarce and weakly marked systematic punctures. Prosternum flat. Posterior elevation of mesoventrite elevated as a triangular pyramid, with posterior face somewhat bisinuate and medial longitudinal ridge extending anteriorly (resembling a nose). ***Abdomen***: Apical emargination of fifth ventrite relatively deep, U-shaped. ***Aedeagus***: (Fig. 25A–D) Overall shape pear-like, 3× longer than wide; lateral projection on apical region of outer margin of each paramere pointed; at closest point, dorsal inner margins of parameres separated by distance slightly narrower than greatest width of a paramere; dorsal plate of median lobe with neck 1/2 as broad as base; arms of dorsal plate of median lobe slightly converging, nearly 0.3× length of dorsal plate of median lobe; each arm slightly widened near base, then gradually slightly narrowing towards apex, with apex medially and dorsally pointed; notch between arms at base slightly narrower than base of an arm; ventral plate of median lobe weakly sclerotized, triangular, apically acuminate, apex not reaching base of fork of dorsal plate; basal piece 0.3× length of a paramere. In lateral view, aedeagus with ventral outline of parameres 5× longer than greatest width near base; dorsal outline of aedeagus in lateral view nearly straight along basal 1/3 and evenly convex along second 1/3.

##### Etymology.

*Duo* (L.), meaning two, referring to the prominent two-pronged dorsal plate of the median lobe.

##### Distribution.

Brazil (Amazonas, Pará), French Guiana, Guyana, Suriname, Venezuela (Fig. 29A).

##### Habitat.

This species has been collected from forested detrital pools, especially those associated with riparian habitats.

#### ﻿ Novochares florifer sp. nov.

Taxon classificationAnimaliaColeopteraHydrophilidae

7E1A95C6-19C2-51F4-B177-88C8113A7895

https://zoobank.org/C697FFB9-CC3F-4C56-A1ED-DE615398DDEA

Figs 28D–F , 29D

##### Type material.

***Holotype* (male)**: “BRAZIL: Amazonas: Tapauá/ -5.50298°, -62.12392°; 54 m/ c. 240 km N. Humaita on BR-319/ 12.vii.2018; leg. Short; forest/ detrital pool; BR18-0712-01B”, “DNA VOUCHER/ Extraction #/ SLE-1991” (INPA).

##### Differential diagnosis.

The aedeagus of this species is spectacular (Fig. 28D) and not even close to any other described species of the genus.

##### Description.

**Size and form**: Body length 7.3 mm. ***Coloration***: Dorsal surfaces brown and sheeny. ***Head***: Maxillary palps nearly 1.5× longer than width of head, uniformly brown in color. ***Thorax***: Ground punctation on pronotum and elytra dense and very shallowly impressed. Elytra without rows of serial punctures, each with very faint rows of scarce and weakly marked systematic punctures on lateral surface of elytron. Posterior elevation of mesoventrite elevated as a triangular pyramid, with posterior face somewhat bisinuate and medial longitudinal ridge extending anteriorly (resembling a nose). ***Abdomen***: Apical emargination of fifth ventrite relatively deep, U-shaped. ***Aedeagus***: (Fig. 28D–F) Overall shape house-like, 2.4× longer than wide; lateral projection on apical region of outer margin of each paramere strongly pointed; apical regions of parameres strongly converging; at closest point, dorsal inner margins of parameres separated by distance slightly 0.2× greatest width of a paramere; dorsal plate of median lobe constricted near mid-length; arms of dorsal plate of median lobe diverging, nearly 0.35× length of dorsal plate of median lobe; each arm somewhat sickle-shaped, dorsally concave, with apex dorsally pointed; notch between arms at base slightly narrower than base of an arm; ventral plate of median lobe weakly sclerotized, tongue-like, apically broadly rounded, apex extending beyond apex of arms of dorsal plate; basal piece 0.35× length of a paramere. In lateral view, aedeagus with ventral outline of parameres 0.38× longer than greatest width near base; dorsal outline of aedeagus in lateral view nearly straight along basal 1/2.

##### Etymology.

*Florifer* (L.), meaning flowery, in reference to the intricate and beautiful shape of the aedeagus.

##### Distribution.

Only known from the type locality in the Brazilian Amazon (Fig. 29D).

##### Habitat.

The holotype was collected in a small forested detrital pool that was adjacent to a small sandy creek.

##### Remarks.

The description of *Novocharesflorifer* is based on a single specimen that was extracted for DNA. The specimen is mounted in pieces on a card, and the colors described here may not match freshly collected material.

#### ﻿ Novochares mojenos sp. nov.

Taxon classificationAnimaliaColeopteraHydrophilidae

D2797DF7-E7E4-59CD-870D-3C82B3E2FE75

https://zoobank.org/C559FBF1-523B-425D-AFF8-F0C9C9E7BD81

Figs 26D–F , 29D

##### Type material.

***Holotype* (male)**: “BOLIVIA: Beni Department/ Cercado Province/ 9.5 km N. of Trinidad/ 14°46'34"S, 64°58'00"W/ 17.vi.1999, leg. K.B. Miller” (SEMC). ***Paratype* (1 ex.)**: same data as holotype (1, SEMC).

##### Differential diagnosis.

The aedeagus of this species is most similar to *N.yora*, with which it shares a similar overall form. In *N.yora* the dorsal plate of the median lobe is thinner along its neck, and the arms of the apical fork are narrower and parallel sided (Fig. 26A), while they are wider and more curved in *N.mojenos* (Fig. 26D). The outer margins of the parameres are also more sinuate in *N.mojenos*, while straight in *N.yora*.

##### Description.

Body length 7.2–7.3 mm. ***Coloration***: Dorsal surfaces brown, with slightly paler (orange) clypeus, labrum, and margins of pronotum and elytra. ***Head***: Maxillary palps nearly 1.3× longer than width of head, uniformly orange in color. ***Thorax***: Ground punctation on pronotum and elytra dense and very shallowly impressed. Elytra without rows of serial punctures, each with very faint rows of scarce and weakly marked systematic punctures on lateral surface of elytron. Prosternum flat. Posterior elevation of mesoventrite elevated as a triangular pyramid, with posterior face somewhat bisinuate and medial longitudinal ridge extending anteriorly (resembling a nose). ***Abdomen***: Apical emargination of fifth ventrite relatively deep, U-shaped. ***Aedeagus***: (Fig. 26D–F) Overall shape pear-like, 2.8× longer than wide, with outer lateral margins of parameres slightly sinuate along second 1/3; lateral projection on apical region of outer margin of each paramere pointed and ventrally directed; at closest point, dorsal inner margins of parameres separated by distance slightly narrower than greatest width of a paramere; dorsal plate of median lobe with neck 0.3× as broad as base; arms of dorsal plate of median lobe slightly converging, nearly 0.15× length of dorsal plate of median lobe; each arm widened near apical 1/3, then strongly narrowing, forming medially and dorsally pointed apex; notch between arms at base narrower than base of an arm; ventral plate of median lobe weakly sclerotized, triangular, rounded at apex, apex extending to mid-length of neck of dorsal plate; basal piece 0.3× length of a paramere. In lateral view, aedeagus with ventral outline of parameres 3.8× longer than greatest width near base; dorsal outline of aedeagus in lateral view nearly straight along basal 2/3.

##### Etymology.

Mojenos, in reference to the Mojenos indigenous group.

##### Distribution.

Only known from the type locality in Bolivia (Fig. 29D).

##### Habitat.

Nothing is known about the habitat of this species.

#### ﻿ Novochares mura sp. nov.

Taxon classificationAnimaliaColeopteraHydrophilidae

E45C1F9D-B369-582A-80FF-F7D8E3A6ADBD

https://zoobank.org/709EF56F-5B9A-4B99-8E14-F1041D6C5254

Figs 5C , 27A–C , 29B

##### Type material.

***Holotype* (male)**: “BRAZIL: Amazonas: Tapauá/ -5.50298°, -62.12392°; 54 m/ c. 240 km N. Humaita on BR-319/ 12.vii.2018; leg. Short; forest/ detrital pool; BR18-0712-01B”, “DNA VOUCHER/ Extraction #/ SLE-1973” (INPA).

##### Differential diagnosis.

See differential diagnosis for *N.danta*.

##### Description.

Body length 6.9 mm. ***Coloration***: Dorsal surfaces brown and sheeny. ***Head***: Maxillary palps nearly 1.5× longer than width of head, uniformly brown in color. ***Thorax***: Ground punctation on pronotum and elytra dense and very shallowly impressed. Elytra without rows of serial punctures, each with very faint rows of scarce and weakly marked systematic punctures on lateral surface. Prosternum very weakly and broadly convex. Posterior elevation of mesoventrite elevated as a triangular pyramid, with posterior face somewhat bisinuate and medial longitudinal ridge extending anteriorly (resembling a nose). ***Abdomen***: Apical emargination of fifth ventrite small and shallow, slightly broader than deep. ***Aedeagus***: (Figs 5C, 27A–C) Overall shape oval, 1.9× longer than wide; apical region of parameres strongly sclerotized, somewhat triangular, rounded at apex; dorsal inner margin of each paramere medially pointed at base of fork of dorsal plate of median lobe; dorsal plate of median lobe with base 8× broader than neck; arms of dorsal plate of median lobe weakly dorsally concave, widened at mid-length, nearly 0.23× length of dorsal plate of median lobe, with inner margins slightly converging; each arm apically rounded; notch between arms at base slightly narrower than base of an arm; ventral plate of median lobe moderately sclerotized, bullet-shaped, with nearly parallel lateral margins, roundly converging to apex, apex narrowly and sharply acuminate, extending beyond base of fork of dorsal plate of median lobe, for 1/5 the length of an arm; basal piece 0.34× length of a paramere. In lateral view, aedeagus parallelogram-shaped, with ventral outline of parameres 3.6× longer than greatest width near base; dorsal outline of aedeagus in lateral view sinuate, medially straight.

##### Etymology.

Mura, in reference to the Mura indigenous group.

##### Distribution.

Only known from the type locality in the Brazilian Amazon (Fig. 29B).

##### Habitat.

The holotype was collected in a small forested detrital pool that was adjacent to a small sandy creek.

##### Remarks.

The description of *Novocharesmura* is based on a single specimen that was extracted for DNA. The colors described here may not match freshly collected material.

#### ﻿ Novochares piaroa sp. nov.

Taxon classificationAnimaliaColeopteraHydrophilidae

0476D3EE-AC17-5CC5-8E47-A4D7F288A76A

https://zoobank.org/A129C9A5-CB42-4C2A-BB5A-9921126C30B1

Figs 24D , 28A–C , 29D

##### Type material.

***Holotype* (male)**: “VENEZUELA: Amazonas State/ Communidad Cano Gato/ 04°45.845'N, 67°44.345'W, 100m/ 7.i.2006; stream margin/detritus/ AS-06-016; leg. A.E.Z. Short” (MIZA). ***Paratypes* (12 exs.): Venezuela: Amazonas**: Same data as holotype (6, SEMC, including DNA Voucher SLE1194, TTU-Z); same locality but 16.i.2009, leg. Short, Miller, Camacho, Joly, & García, along stream, VZ09-0116-01X (4, MIZA, SEMC).

##### Differential diagnosis.

This species has an aedeagus that superficially resembles several other *tectiformis* group species, but is fairly distinct from them all. It is perhaps most close to *N.duo* (Fig. 25A) and *N.coya* (Fig. 26G), but the dorsal plate of the median lobe is much shorter and the apical fork much smaller (Fig. 28A) than in either of those species.

##### Description.

Body length 6.8–7.8 mm. ***Coloration***: Dorsal surfaces dark brown, with very slightly paler margins of pronotum and elytra. ***Head***: Maxillary palps nearly 1.6× longer than width of head, uniformly orange to brown in color (Fig. 24D). ***Thorax***: Ground punctation on pronotum and elytra dense and very shallowly impressed. Elytra without rows of serial punctures, each with very faint rows (one dorsal and two or three lateral) of scarce and weakly marked systematic punctures. Prosternum very weakly and broadly convex. Posterior elevation of mesoventrite elevated as a triangular pyramid, with posterior face somewhat concave and medial longitudinal ridge extending anteriorly. ***Abdomen***: Apical emargination of fifth ventrite relatively deep, U-shaped. ***Aedeagus***: (Fig. 28A–C) Overall shape pear-like, 2.4× longer than wide; lateral projection on apical region of outer margin of each paramere pointed; at closest point, dorsal inner margins of parameres separated by distance nearly 1/3 greatest width of a paramere; dorsal plate of median lobe with neck 1/2 as broad as base; arms of dorsal plate of median lobe short, nearly 0.15× length of dorsal plate of median lobe; each arm triangular, dorsally concave, with apex acute and dorsally pointed; notch between arms at base nearly 1/2 as broad as base of an arm; ventral plate of median lobe moderately sclerotized, triangular, apically narrowly rounded, apex extending to beyond mid-length of neck, not reaching base of fork of dorsal plate; basal piece 0.35× length of a paramere. In lateral view, aedeagus flattened, with ventral outline of parameres 3× longer than greatest width near mid-length; dorsal outline weakly and evenly convex along basal 2/3.

##### Etymology.

Piaroa, in reference to the Piaroa indigenous group.

##### Distribution.

Only known from the type locality in the Guiana Shield region of Southern Venezuela (Fig. 29D).

##### Habitat.

The only known series was collected along the margins of a sandy stream with lots of detritus.

#### ﻿ Novochares pume sp. nov.

Taxon classificationAnimaliaColeopteraHydrophilidae

2E1EC6C7-8561-5D04-AA84-048147D3BCB3

https://zoobank.org/CC320A36-1C35-4EF9-94BA-918353C4A27C

Figs 28G–J , 29D

##### Type material.

***Holotype* (male)**: “VENEZUELA: Guárico, Hato/ Masaguaral, 45kmS Calabozo/ 8.57N, 67.58W, Savanna #12/ 75 m, 15July1989, uv light/ M. Epstein & M. Deza” (USNM). ***Paratypes* (4 exs.): Venezuela: Barinas**: Barinas, 23.ii.1969, leg. P. & P. Spangler (1, USNM). **Cojedes**: Galeras del Pao, 175 m, 26.vi.1963, leg. C.J. Rosales & A. Perez (3, MIZA, SEMC).

##### Differential diagnosis.

See differential diagnosis of *N.tectiformis*.

##### Description.

Body length 6.4–7.9 mm. ***Coloration***: Dorsal surfaces dark brown, with very slightly paler margins of pronotum and elytra. ***Head***: Maxillary palps nearly 1.3× longer than width of head, uniformly orange to brown in color. ***Thorax***: Ground punctation on pronotum and elytra dense and very shallowly impressed. Elytra without rows of serial punctures, each with very faint rows of scarce and weakly marked systematic punctures on lateral surface. Prosternum flat. Posterior elevation of mesoventrite with posterior face somewhat bisinuate and medial longitudinal ridge extending anteriorly (resembling a nose). ***Abdomen***: Apical emargination of fifth ventrite relatively deep, U-shaped. ***Aedeagus***: (Fig. 28G–J) Overall shape pear-like, 3× longer than wide; lateral projection on apical region of outer margin of each paramere strongly pointed; at closest point, dorsal inner margins of parameres separated by distance nearly 1/3 greatest width of a paramere; dorsal plate of median lobe with neck 0.4× as broad as base; arms of dorsal plate of median lobe gradually and weakly narrowing towards apex, apically converging, with apex narrowly rounded and dorsally pointed, nearly 0.3× length of dorsal plate of median lobe; notch between arms slightly projected at base, at base nearly as broad as base of an arm; ventral plate of median lobe moderately sclerotized, triangular, apically pointed, apex extending to apex of arms of dorsal plate; basal piece 0.28× length of a paramere. In lateral view, aedeagus flattened, with ventral outline of parameres 4.2× longer than greatest width near base; dorsal outline nearly straight along basal 2/3.

##### Etymology.

Pume, in reference to the Pume indigenous group.

##### Distribution.

This species is known from several localities in the llanos region of Venezuela (Fig. 29D).

##### Habitat.

The holotype was collected at a UV light in a savanna. Nothing else is known about this species.

#### ﻿ Novochares tambopatense sp. nov.

Taxon classificationAnimaliaColeopteraHydrophilidae

C45707F3-4078-5E69-8A8B-C12C38559657

https://zoobank.org/9AA0FE31-8967-4B49-8F26-3FD630A3C793

Figs 28K, L , 29D

##### Type material.

***Holotype* (male)**: “PERU: Madre de Dios: Tambopata/ -12.53550°S, -69.01205°W, 190m/ Kawsay Biological Station/ margin of forested creek; 3.vi.2022/ PE22-0603-02A; leg. Short et al.” (MHNSM). ***Paratypes* (5 exs.): Peru: Madre de Dios**: same data as holotype except large detrital pool nr. Banana area, PE22-0603-02B (3, MHNSM, SEMC); same data as holotype except 2.vi.2022, swamp pools by creek, PE22-0602-02A (2, SEMC).

##### Differential diagnosis.

See differential diagnosis of *N.tectiformis*.

##### Description.

Body length 7.0–7.9 mm. ***Coloration***: Dorsal surfaces dark brown and sheeny, with slightly paler margins of pronotum and elytra. ***Head***: Maxillary palps nearly 1.4× longer than width of head, uniformly orange to brown in color. ***Thorax***: Ground punctation on pronotum and elytra dense and very shallowly impressed. Elytra without rows of serial punctures, each with very faint rows (one dorsal and two or three lateral) of scarce and weakly marked systematic punctures. Prosternum very weakly and broadly convex. Posterior elevation of mesoventrite with posterior face somewhat bisinuate and medial longitudinal ridge extending anteriorly (resembling a nose). ***Abdomen***: Apical emargination of fifth ventrite relatively deep, U-shaped. ***Aedeagus***: (Fig. 28K, L) Overall shape pear-like, 2.4× longer than wide; lateral projection on apical region of outer margin of each paramere strongly pointed; at closest point, dorsal inner margins of parameres separated by distance as wide as a paramere; dorsal plate of median lobe gradually narrowing towards base of fork; arms of dorsal plate of median lobe gradually narrowing towards apex, apically pointed and weakly converging, nearly 0.3× length of dorsal plate of median lobe; notch between arms broadly and roundly projected at base, at base nearly as broad as neck; ventral plate of median lobe strongly sclerotized, triangular, apically pointed, apex extending to mid-length of arms of dorsal plate; basal piece 0.36× length of a paramere. In lateral view, aedeagus flattened, with ventral outline of parameres 4.5× longer than greatest width near base; dorsal outline nearly straight along basal 2/3.

##### Etymology.

Named after the people from the Tambopata region in Peru.

##### Distribution.

Known only from Kawsay Biological Station in Peru (Fig. 29D).

##### Habitat.

This species was collected from several large swampy pools full of detritus in the forest.

#### ﻿ Novochares tectiformis

Taxon classificationAnimaliaColeopteraHydrophilidae

(Fernández, 1982)

8046E357-8A45-564A-B3D5-57D8872FEA3C

Figs 5B , 6A , 24A , 25G–L , 29C

Helochares (s. str.) tectiformis Fernández, 1982b: 88; Fernández 1989: 148 [in key].
Novochares tectiformis (Fernández, 1982); Girón and Short 2021: 206.

##### Type material.

***Holotype*** male from Argentina (Corrientes, Santo Tomé) deposited in MACN (not seen).

##### Material examined

**(310 exs). Bolivia: Beni**: Cercado Province, 9.5 km N of Trinidad, 14°46'34"S, 64°58'00"W, 17.vi.1999, leg. K.B. Miller (1, SEMC), 7 km SW of Trinidad, 14°52'12"S, 64°57'32"W, 163 m, 18.vii.1998, leg. K.B. Miller (9, SEMC). **Santa Cruz**: 3.7 km SSE Buena Vista, Hotel Flora y Fauna, 23-30.iv.2004, leg. A.R. Cline, MV+HG lights (56, SEMC, TTU-Z); same data but 1–12.v.2004 (39, SEMC). **Brazil: Amapá**: Oiapoque (ca. 22 km S) on BR-156, leg. Short, forested detrital pools, BR18-0720-01B (20, INPA, SEMC, TTU-Z); Calcoene (ca. 50 km NW) on BR-156, 2.67956, -51.35353, 46 m, 21.vii.2018, leg. Short, detrital pool in forest by creek, BR18-0721-01B (1, SEMC). **Amazonas**: Apui (ca. 43 km NW), -6.96828, -60.06702, 60 m, 4.vii.2018, leg. Short, backwater margin of river, BR18-0704-02C (1, SEMC, DNA voucher SLE1905); Tapauá, Humaita (ca. 240 km N) on BR-319, -5.50298, -62.12392, 54 m, 11.vii.2018, leg. Short, forested detrital pool, BR18-0712-01A (1, SEMC, DNA Voucher SLE1981); same data except margins of small forested stream, BR18-0712-01B (5, SEMC). **Matto Grosso do Sul**: MS-243, 3 km SW of jct with BR-262, -20.09539, -56.78108, 147 m, 26.vi.2018, leg. Hamada & team, small drying marsh along road by cattle pasture; BR18-0626-02A (19, SEMC, including DNA voucher SLE2095); Aquidauana (ca. 5 km S) on MS-174, -20.53416, -55.76038, 166 m, 27.vi.2018, leg. Hamada & team, small shallow pond with dense vegetation, BR18-0627-02A (1, SEMC, DNA voucher SLE2093). **Paraná**: Curitiba, 28.vi.1969, leg. P. & P. Spangler (1, UNSM). **Rondônia**: Novo Uniao, Vale do Cachoeiras, -10.91764, -62.377, 359 m, 10.vii.2018, leg. Short, small sandy-bottom stream margin, BR18-0710-02A (1, SEMC, DNA voucher SLE2089). **Roraima**: BR-401, ca. 26 km NE of Boa Vista, 2°56.191'N, 60°28.017'W, 92 m, 12.i.2018, leg. Short, pooled up morichal, BR18-0112-06B (2, SEMC); BR-401, ca. 6 km SW of Bonfim, 3°21.615'N, 59°53.361'W, 100 m, 12.i.2018, leg. Short, Benetti & Santana, large marsh with abundant vegetation, BR18-0112-02A (1, SEMC); BR-174, ca. 50 km NW Boa Vista, 3°18.348'N, 60°51.458'W, 100 m, 13.i.2018, leg. Short, marsh, BR18-0113-02A (1, SEMC); Amajari, ca. 16 km W on RR-203, 3°36.874'N, 61°33.470'W, 125 m, leg. Short, Benetti & Santana, marsh, BR18-0113-04A (43, INPA, SEMC). **São Paulo**: Guaratingueta, at light, 17.iv.1960, leg. B. Malkin #1 (7, UNSM); “São Paulo”, “10-57”, V. N. Alin (1, USNM). **Ecuador: Pastaza**: AGIP platform Villano B, along transect 1 & 2, 24.v.2008; leg. A.E.Z. Short, small forest stream, AS-08-008b (1, SEMC). **French Guiana**: Anapaike Village, Lawa River, 22–25.ix1963, leg. B. Malkin (2, USNM); Carbet ONF Montagne de fer, Piste de montagne de fer (formerly road Degrad Florian), Crique Petit Laussat, 5.40697°N, -53.55468°W, 10 m, leg. Short, detrital pools, FG20-0302-01C (6, SCC, SEMC); Carbet communal St-Elie, Route de Saint-Elie, tributary of Crique Toussaint, 5.29653°N, -53.05205°W, 42 m, leg. Short & Neff, margins of clearwater stream, FG20-0305-03B (3, SEMC); Paracou, Station de recherche CIRAD, Crique Verlot, 5.27966°N, -52.92846°W, 8 m, leg. Short & Neff, forested detrital pools, FG20-0306-01A (3, SEMC); same data except margins and detrital snags in stream; FG20-0306-01B (1, SEMC). **Guyana: Region 7**: Takatu Mts, logging site, forest puddle, 8.xii.1983, leg. P.D. Perkins (1, SEMC), same data but without logging site and only “xii.1983” (5, SEMC). **Region 9**: Tributary of the Takatu River, NW of Kusad Mts., 2°50.563'N, 59°59.113'W, 109 m, 24.x.2013, leg. Short, Isaacs, & Salisbury, vegetated creek margins, GY13-1024-02B (11, CBDG, SEMC); Ziida Karisihizi (Lake), nr. Kusad Mts., 2°49.793'N, 59°48.361'W, 123 m, 25.x.2013, leg. Short, Isaacs, & Salisbury, large vegetated marsh, GY13-1025-01A (30, SEMC, including DNA voucher SLE1220); Ziida Wao Creek near Kusad Mountains, 2°49.724'N, 59°48.546'W, 121 m, 25.x.2013, leg. Short, Isaacs, and Salisbury, stagnant vegetated creek, GY13-1025-02A (12, SEMC); nr. Kusad Mts., 2°50.955'N, 59°58.353'W, 115 m, 27.x.2013, leg. Short, Isaacs, & Salisbury, vegetated pond, GY13-1027-02A (1, SEMC); nr. Kusad Mts., 2°51.193'N, 59°55.336'W, 117 m, 28.x.2013, leg. A. Short, muddy margins of vegetated farm ponds, GY13-1028-02A (1, SEMC); N. Parabara, 2°10.902'N, 59°20.547'W, 260 m, basecamp area, 31.x.2013, leg. A. Short marshy puddles & rivulets; GY13-1031-03A (1, SEMC). **Suriname: Para**: Along Martin Luther King Highway, marsh by road, 23.vii.2012, leg. Short & team, SR12-0723-02A (1, SEMC). **Sipaliwini**: Camp 3, Werehpai, 2°21.776'N, 56°41.861'W, 237 m, 3–7.ix.2010, leg. Short and Kadosoe, pooled up detrital creek, SR10-0903-01A (1, SEMC, DNA voucher SLE448); Raleighvallen Nature Reserve, base of Voltzberg, 4°40.432’N, 56°11.079’W, 86 m, 16.iii.2016, leg. Short et al., pooled up stream, SR16-0316-01B (1, SEMC); Upper Palumeu, Camp 1, 2.47700°N, 55.62941°W, 275 m. leg. A. Short, 10–16.iii.2012, Flight Intercept Trap, SR12-0310-TN1 (1, SEMC); same data except small forest pool, SR12-0310-02A (2, SEMC); Kasikasima, Camp 4 (low), 2.97731°N, 55.38500°W, 200 m, 20–25.iii.2012leg. A. Short, detrital pools along trail to METS camp, SR12-0320-03A (4, SEMC); Raleighvallen Nature Reserve Voltzberg Station, 04°40.910'N, 56°11.138'W, 78 m, 29.vii.2012, leg. A. Short and C. McIntosh, detrital side pool, SR12-0729-02B (3, SEMC); same data as previous except: 29.vii.2012, leg. Short, Maier, McIntosh, and Kadosoe, stream margins, SR12-0729-02A (4 NZSC, SEMC). **Suriname**: Krakka-Phedra Road, 25.x.1962, leg. B. Malkin, “tiny pool in forest, much fallen foliage” (92, UNSM). **Venezuela: Bolívar**: Guri, Rio Caroni, 100 m, 16.xi.1966, leg. J. & B. Bechyne & E. Osuna (1, MIZA); Gran Sabana, E. Pauji, 4°36.635'N, 61°26.133'W, 894 m, 17.vii.2010, leg. Short, roadside puddles, VZ10-0717-01B (1, SEMC); Gran Sabana, N. Santa Elena, Rio Guara at Rt. 10, 17.vii.2010, leg. Short, Tellez, & Arias, marshy area, VZ10-0717-02A (3, SEMC).

##### Differential diagnosis.

This common and widespread species has an aedeagal form that is similar to two relatively rare and (so far as we know) localized species: *N.tambopatense* (Fig. 28K) and to a lesser extent *N.pume* (Fig. 28G). The ventral plate of the median lobe is strongly extended into a long spine in both of those species, which projects distally making the median lobe appear trifid. In *N.tectiformis*, the ventral plate of the median lobe does not project distally (e.g., Fig. 25K) or only very little (e.g., Fig. 25L) and is never in the form of a narrow spine. In *N.pume*, the projections of the ventral and dorsal plates are subequal in size, while in *N.tambopatense*, the projection of the ventral plate does not reach the apex of the projections of the dorsal plate.

##### Description.

Body length 6.2–9.5 mm. ***Coloration***: Dorsal surfaces dark brown and sheeny, with slightly to sharply paler margins of pronotum and elytra. ***Head***: Maxillary palps 1.1–1.5× longer than width of head, uniformly orange to brown in color (Fig. 24A). ***Thorax***: Ground punctation on pronotum and elytra dense and very shallowly impressed. Elytra without rows of serial punctures, each with very faint rows (one dorsal and two or three lateral) of scarce and weakly marked systematic punctures. Prosternum flat to very weakly and broadly convex. Posterior elevation of mesoventrite with posterior face somewhat bisinuate and medial longitudinal ridge extending anteriorly (resembling a nose). ***Abdomen***: Apical emargination of fifth ventrite relatively deep, U-shaped. ***Aedeagus***: (Figs 5B, 25G–L) Overall shape pear-like, 2.4–3.0× longer than wide; lateral projection on apical region of outer margin of each paramere strongly pointed; at closest point, dorsal inner margins of parameres separated by distance 0.1–0.5× greatest width of a paramere; dorsal plate of median lobe with neck 0.5–0.6× as broad as base; arms of dorsal plate of median lobe gradually and weakly narrowing towards apex, apically converging or parallel, with apex narrowly and dorsally pointed, nearly 0.35–0.50× length of dorsal plate of median lobe; notch between arms broadly projected at base, at base nearly as broad as 2–3× base of an arm; ventral plate of median lobe moderately sclerotized, triangular, apically rounded, apex extending to basal 1/4 of arms of dorsal plate; basal piece 0.33–0.34× length of a paramere. In lateral view, aedeagus flattened, with ventral outline of parameres 3.9× longer than greatest width near base; dorsal outline nearly straight along basal 2/3.

##### Distribution.

Previously recorded for Argentina, Brazil (Mato Grosso do Sul), Paraguay, and Venezuela. Here newly recorded for Bolivia, Guyana, Ecuador, French Guiana, Suriname, and the Brazilian states of Amapá, Amazonas, Paraná, Rondônia, and São Paulo (Fig. 29C).

##### Habitat.

This species is found in a variety of habitats, with a particular preference for detrital pools. However, it has also been found in marshes and stream margins.

##### Remarks.

This species exhibits a relatively large degree of morphological and genetic variation across its substantial geographic range. The maximum intraspecific pairwise genetic divergence in COI is 5.4% among the individuals sequenced from Suriname, Guyana, and Brazil. While the overall form of the aedeagus is always the same, there is variation in the degree of convergence of the distal arms of the dorsal plate of the median lobe (compare Fig. 25K vs. Fig. 25L) as well as small differences in the relative height and shape of the apex of the ventral plate of the median lobe. However, we did not find these differences to correlate to the genetic data we had, nor to be stable or substantial enough to break the group into multiple species. Therefore, we consider *N.tectiformis* to be a somewhat variable, common, and widespread species.

#### ﻿ Novochares trifurcatus sp. nov.

Taxon classificationAnimaliaColeopteraHydrophilidae

B6224A73-7E74-5A2F-9D1E-EE7D5384A1F4

https://zoobank.org/E3A3003E-AB1D-41F8-8B38-0567164B88C0

Figs 5A , 27I, J , 29B

##### Type material.

***Holotype* (male)**: “PERU: Loreto: Maynas Province/ 3°50.430’S, 73°22.847'W, 116 m/ ca 10km SW Iquitos, nr. Ninarumi/ leg. S.Baca, 18.i.2020; margin of/ large pond; PE20-0118-02A”, “DNA VOUCHER/ Extraction #/ SLE-2147” (MHNSM).

##### Differential diagnosis.

The aedeagal form of this species is most similar to *N.xingu* (Fig. 27D), but that species does not have an elevated ventral plate of the median lobe, so only appears bifid instead of trifid as in this species (Fig. 27I). Additionally, *N.trifurcatus* is unusual among species of the *tectiformis* species group in that the mesoventrite is not strongly elevated.

##### Description.

Body length 7.0 mm. ***Coloration***: Dorsal surfaces dark brown and sheeny. ***Head***: Maxillary palps nearly 1.6× longer than width of head, uniformly reddish brown in color. ***Thorax***: Ground punctation on pronotum and elytra dense and very shallowly impressed. Elytra without rows of serial punctures, each with very faint rows (one dorsal and two or three lateral) of scarce and weakly marked systematic punctures. Prosternum flat. Posterior elevation of mesoventrite somewhat transverse and weakly elevated, with low medial longitudinal ridge extending anteriorly. ***Abdomen***: Apical emargination of fifth ventrite relatively deep, U-shaped. ***Aedeagus***: (Figs 5A, 27I, J) Overall shape pear-like, 2.8× longer than wide; lateral projection on apical region of outer margin of each paramere strongly pointed and ventrally directed; at closest point, dorsal inner margins of parameres separated by distance nearly 1/2 greatest width of a paramere; dorsal plate of median lobe with neck 0.8× as broad as base; arms of dorsal plate of median lobe gradually and weakly narrowing up to mid-length, then uniform in width, slightly curved and apically converging, with apex narrowly rounded and dorsally pointed, nearly 0.24× length of dorsal plate of median lobe; notch between arms at base slightly narrower than base of an arm; ventral plate of median lobe strongly sclerotized, triangular, apically pointed, apex extending nearly to apex of arms of dorsal plate; basal piece 0.29× length of a paramere. In lateral view, aedeagus with ventral outline of parameres 2.8× longer than greatest width near base; dorsal outline nearly straight along basal 1/2, then evenly convex along third 1/4.

##### Etymology.

*Trifurcatus* (L.), in reference to the three-pronged appearance of the median lobe of the aedeagus, formed by the lateral arms of the dorsal plate and the median projection of the ventral plate.

##### Distribution.

Only known from the type locality in Peru (Fig. 29B).

##### Habitat.

The holotype was collected from the margin of a large pond.

##### Remarks.

The description of *Novocharestrifurcatus* is based on a single specimen that was extracted for DNA. The colors described here may not match freshly collected material.

#### ﻿ Novochares xingu sp. nov.

Taxon classificationAnimaliaColeopteraHydrophilidae

1C462888-E1A7-524E-9267-640BD5538662

https://zoobank.org/678985D5-F6F5-4AA1-A07A-DAA5497F4088

Figs 27D, E , 29B

##### Type material.

***Holotype* (male)**: “BRAZIL: Pará: Rio Xingu/ Camp (3°39'S, 52°22'W)/ Altamira (ca. 60 km S.)/ 10 Oct 1986/ P. Spangler & O. Flint”, “Colln. #19, 1^st^ jungle/ stream on trail 4” (USNM). ***Paratypes* (3 exs.): Brazil: Pará**: Same data as type except 12.x.1986, pond at 2^nd^ palm grove on trail 1, Colln. #21 (1, SEMC); same data as type except 15.x.1986, pond at 2^nd^ palm grove on trail 1, Colln. #24 (2, USNM).

##### Differential diagnosis.

See differential diagnosis for *N.trifurcatus*.

##### Description.

Body length 7.6–8.0 mm. ***Coloration***: Dorsal surfaces dark brown and sheeny, with slightly paler margins of pronotum and elytra. ***Head***: Maxillary palps nearly 1.4× longer than width of head, uniformly orange to brown in color. ***Thorax***: Ground punctation on pronotum and elytra dense and very shallowly impressed. Elytra without rows of serial punctures, each with very faint rows (one dorsal and two or three lateral) of scarce and weakly marked systematic punctures. Prosternum flat. Posterior elevation of mesoventrite with posterior face somewhat bisinuate and medial longitudinal ridge extending anteriorly (resembling a nose). ***Abdomen***: Apical emargination of fifth ventrite relatively deep, U-shaped. ***Aedeagus***: (Fig. 27D, E) Overall shape pear-like, 2.9× longer than wide; lateral projection on apical region of outer margin of each paramere pointed; at closest point, dorsal inner margins of parameres separated by distance 0.5× greatest width of a paramere; dorsal plate of median lobe with neck 0.6× as broad as base; arms of dorsal plate of median lobe gradually and weakly narrowing towards apex, parallel, with apex narrowly and dorsally pointed, nearly 0.25× length of dorsal plate of median lobe; notch between arms at base slightly narrower than base of an arm; ventral plate of median lobe weakly sclerotized, triangular, apically acute, apex extending to mid-length of dorsal plate; basal piece 0.33× length of a paramere. In lateral view, aedeagus flattened, somewhat triangular, with ventral outline of parameres 5.8× longer than greatest width near base; dorsal outline nearly straight.

##### Etymology.

Xingu, in reference to the Xingu peoples of the region where this species is found.

##### Distribution.

Known only from several collections at the type locality in Brazil (Pará) (Fig. 29B).

##### Habitat.

The label data indicate specimens were taken from a “jungle stream” and a “pond”.

#### ﻿ Novochares yora sp. nov.

Taxon classificationAnimaliaColeopteraHydrophilidae

E1A410FA-5F48-58EC-B71B-F77D83E9978B

https://zoobank.org/A1D0C105-0058-4277-A035-5176C1EBF3C3

Figs 26A–C , 29B

##### Type material.

***Holotype* (male)**: “PERU: Cuzco/ Pilcopata, 600m.; 8-10.xii.1979/ premontane moist forest/ leg. J. B. Heppner” (USNM). ***Paratypes* (6 exs.): Peru: Cuzco**: same data as holotype (3, USNM, SEMC). **Madre de Dios**: Manu, Pakitza, 12°7'S, 70°58'W, 250 m, 18.viii.1988, UV light, leg. O. Flint & N. Adams (1, USNM); same locality but 23.ix.1989, leg. R.W. Bouchard, stream (2, USNM); same locality but 19.ix.1989, leg. R.A. Faitoute, Berlese leaf litter, colln 47a (1, USNM).

##### Differential diagnosis.

See differential diagnosis of *N.mojenos*.

##### Description.

Body length 7.3–8.6 mm. ***Coloration***: Dorsal surfaces dark brown and sheeny, often with slightly paler margins of pronotum and elytra. ***Head***: Maxillary palps nearly 1.5× longer than width of head, uniformly brown in color. ***Thorax***: Ground punctation on pronotum and elytra dense and very shallowly impressed. Elytra without rows of serial punctures, each with very faint rows (one dorsal and two or three lateral) of scarce and weakly marked systematic punctures. Prosternum flat to very weakly and broadly convex. Posterior elevation of mesoventrite with posterior face somewhat bisinuate and weak medial longitudinal ridge extending anteriorly (resembling a nose). ***Abdomen***: Apical emargination of fifth ventrite relatively narrow, U-shaped. ***Aedeagus***: (Fig. 26A–C) Overall shape pear-like, 2.9× longer than wide; lateral projection on apical region of outer margin of each paramere pointed, ventrally directed; at closest point, dorsal inner margins of parameres separated by distance 0.7× greatest width of a paramere; dorsal plate of median lobe with neck 0.3× as broad as base; arms of dorsal plate of median lobe broadening at mid-length, diverging, with apex angulate and dorsally pointed, nearly 0.19× length of dorsal plate of median lobe; notch between arms at base very narrow; ventral plate of median lobe weakly sclerotized, triangular, apically rounded, apex extending to second 1/3 of dorsal plate; basal piece 0.32× length of a paramere. In lateral view, aedeagus flattened, with ventral outline of parameres 3.5× longer than greatest width near base; dorsal outline nearly straight along second 1/3.

##### Etymology.

Yora, in reference to the Yora peoples of the region where this species is found.

##### Distribution.

Known from several relatively closely situated localities in southern Peru (Fig. 29B).

##### Habitat.

Known specimens were collected at UV lights and at a “stream”.

###### ﻿Incertae sedis

#### ﻿ Novochares inornatus

Taxon classificationAnimaliaColeopteraHydrophilidae

(d’Orchymont, 1926)

6C9DA2A8-29CB-580C-8C4B-D1DCC621AEA9

Helochares (s. str.) inornatus d’Orchymont, 1926: 235.
Novochares inornatus (d’Orchymont, 1926); Girón and Short 2021: 205.

##### Type material.

***Holotype***: female from French Guiana (“Passoura”) deposited in IRNSB (not seen).

##### Other material examined that has been previously identified as this species

**(3 exs.). Brazil**: Rio Sapio, Rio Solimoes, from water in canoe, 21.ii.1874, [identified and labeled as inornatus by J. Balfour-Brown] (1, USNM). **Paraguay**: Sapucay, “WT Foster Collector”, “Helochares (s.str.) sp., prob. inornatus, wait for males” [identification label by d’Orchymont] (1 female, USNM). **Trinidad And Tobago**: Trinidad, “Aug. Busck Collector” “Helochares (s.str.) prob. inornatus, wait for males” [identification label by d’Orchymont] (1 female, USNM).

##### Remarks.

d’Orchymont (1926) described *N.inornatus* from four female specimens (the holotype from French Guiana, and three others from São Paulo, Brazil). The species is described as large (9 mm) and dull black in color. At the time of its description, there were no other large, black species known from South America and it would have been obviously distinct. However, now that we know there are several large and dark species in South America that are essentially externally indistinguishable from each other, the fact that the type series is entirely female presents a problem. Indeed, d’Orchymont seems to have subsequently realized this issue: several female specimens in the USNM collection (from Trinidad and Paraguay) labeled by him as “prob. inornatus, wait for males”.

We note that Balfour-Browne (1939) identified specimens from Guyana and Brazil as *N.inornatus*. From Guyana (Upper Kutari River), he examined one “immature male”, which he dissected and illustrated the aedeagus (fig. 4 in Balfour-Browne 1939). From Brazil (Amazonas: Rio Sappo, Rio Solimoes) he examined one male and two females. Based on the drawing, it seems likely that the specimens that Balfour-Browne examined are of what we have named here as *N.duo* sp. nov. However, there is no way to know if males identified by Balfour-Browne from Guyana and Brazil are conspecific with the type female from French Guiana.

There are several species in French Guiana that match the size and color of *N.inornatus*: *N.duo* sp. nov., *N.tectiformis*, *N.coya*, and *N.orchis* sp. nov. Yet, there is no way at present (morphologically) to be sure which one the female type represents. Therefore, to better stabilize the nomenclature of the genus, we place the name *N.inornatus* as incertae sedis.

## ﻿Discussion

With 52 described species, *Novochares* is now the third largest genus of acidocerine water scavenger beetles, following *Agraphydrus* Klug, 1855 (205 spp.; Girón and Short 2021; Yang et al. 2021a) and *Helochares* Mulsant, 1844 (161 spp.; Girón and Short 2021; Yang et al. 2021b). It is the largest and most widespread genus across the Americas and the Caribbean. Revising this genus took accumulating specimens for more than 50 years, accounting for the efforts of Paul Spangler and more recent series of expeditions across Venezuela, Brazil, the Guianas, Peru, and Suriname.

The fact that most *Novochares* are so uniform externally makes them even more challenging and easily overlooked when looking for traditionally exciting projects in taxonomy and systematics, especially given that most females cannot be accurately identified without molecular data. The aid of molecular data was fundamental for both, making decisions on species limits, and recognizing relationships among taxa. For future studies, we recommend using the same genes and primers used here (see Short and Fikáček 2013) to take advantage of the existing framework to make identifications.

The kinds of habitats where *Novochares* species are found are some of the most typically sampled with standard methods for aquatic macroinvertebrates, which may be the reason why they are relatively abundant in collections, compared to other acidocerines. This revision clearly demonstrates that there is much more to learn about this genus (and acidocerines in general), including learning more about their ecological habits and distributions, and documenting their hidden morphological variation. Exploring their biology, including life cycles and larval morphology, would not only improve our knowledge on the group, but also potentially provide support for the current phylogenetic hypothesis.

There are eight species known from only one specimen and a few more known from two or three specimens, while others are known from various series of specimens from a broad range of localities, which evidences the need for international collaboration across the Americas, especially across the Neotropical region, or at least a strong communication network among water beetle researchers to combine efforts to describe new taxa. Fragmentation of the data due to a lack of communication may result in describing the same species under multiple names, although, we hope that this revision brings enough information to make better decisions when reporting on new species or new localities for these taxa.

## Supplementary Material

XML Treatment for Novochares

XML Treatment for Novochares abbreviatus

XML Treatment for Novochares baca

XML Treatment for Novochares latus

XML Treatment for Novochares oculatus

XML Treatment for Novochares pallipes

XML Treatment for Novochares pilatus

XML Treatment for Novochares aperito

XML Treatment for Novochares bidens

XML Treatment for Novochares furcatus

XML Treatment for Novochares garfo

XML Treatment for Novochares tenedor

XML Treatment for Novochares minor

XML Treatment for Novochares orchis

XML Treatment for Novochares dentatus

XML Treatment for Novochares geminus

XML Treatment for Novochares pertusus

XML Treatment for Novochares punctatostriatus

XML Treatment for Novochares spangleri

XML Treatment for Novochares triangularis

XML Treatment for Novochares atratus

XML Treatment for Novochares bisinuatus

XML Treatment for Novochares chaquensis

XML Treatment for Novochares clavieri

XML Treatment for Novochares cochlearis

XML Treatment for Novochares dicranospathus

XML Treatment for Novochares fernandezae

XML Treatment for Novochares garciai

XML Treatment for Novochares guadelupensis

XML Treatment for Novochares kawsay

XML Treatment for Novochares pastinum

XML Treatment for Novochares pichilingue

XML Treatment for Novochares quadrispinus

XML Treatment for Novochares sallaei

XML Treatment for Novochares tridentis

XML Treatment for Novochares unguis

XML Treatment for Novochares yanomami

XML Treatment for Novochares atlanticus

XML Treatment for Novochares bolivianus

XML Treatment for Novochares coya

XML Treatment for Novochares danta

XML Treatment for Novochares duo

XML Treatment for Novochares florifer

XML Treatment for Novochares mojenos

XML Treatment for Novochares mura

XML Treatment for Novochares piaroa

XML Treatment for Novochares pume

XML Treatment for Novochares tambopatense

XML Treatment for Novochares tectiformis

XML Treatment for Novochares trifurcatus

XML Treatment for Novochares xingu

XML Treatment for Novochares yora

XML Treatment for Novochares inornatus

## ﻿Additional information

### Author ORCIDs

Andrew Edward Z. Short https://orcid.org/0000-0002-7467-7116

Jennifer C. Girón https://orcid.org/0000-0002-0851-6883

### Data availability

All of the data that support the findings of this study are available in the main text.
